# An interpretable predictive deep learning platform for pediatric metabolic diseases

**DOI:** 10.1093/jamia/ocae049

**Published:** 2024-03-18

**Authors:** Hamed Javidi, Arshiya Mariam, Lina Alkhaled, Kevin M Pantalone, Daniel M Rotroff

**Affiliations:** Department of Quantitative Health Sciences, Lerner Research Institute, Cleveland Clinic, Cleveland, OH 44195, United States; Department of Electrical Engineering and Computer Science, Cleveland State University, Cleveland, OH 44115, United States; Center for Quantitative Metabolic Research, Cleveland Clinic, Cleveland, OH 44195, United States; Department of Quantitative Health Sciences, Lerner Research Institute, Cleveland Clinic, Cleveland, OH 44195, United States; Center for Quantitative Metabolic Research, Cleveland Clinic, Cleveland, OH 44195, United States; Center for Quantitative Metabolic Research, Cleveland Clinic, Cleveland, OH 44195, United States; Endocrinology and Metabolism Institute, Cleveland Clinic, Cleveland, OH 44195, United States; Center for Quantitative Metabolic Research, Cleveland Clinic, Cleveland, OH 44195, United States; Endocrinology and Metabolism Institute, Cleveland Clinic, Cleveland, OH 44195, United States; Department of Quantitative Health Sciences, Lerner Research Institute, Cleveland Clinic, Cleveland, OH 44195, United States; Department of Electrical Engineering and Computer Science, Cleveland State University, Cleveland, OH 44115, United States; Center for Quantitative Metabolic Research, Cleveland Clinic, Cleveland, OH 44195, United States; Endocrinology and Metabolism Institute, Cleveland Clinic, Cleveland, OH 44195, United States

**Keywords:** interpretable machine learning, deep learning, pediatric disease prediction, type 2 diabetes, longitudinal data, electronic health record (EHR)

## Abstract

**Objectives:**

Metabolic disease in children is increasing worldwide and predisposes a wide array of chronic comorbid conditions with severe impacts on quality of life. Tools for early detection are needed to promptly intervene to prevent or slow the development of these long-term complications.

**Materials and Methods:**

No clinically available tools are currently in widespread use that can predict the onset of metabolic diseases in pediatric patients. Here, we use interpretable deep learning, leveraging longitudinal clinical measurements, demographical data, and diagnosis codes from electronic health record data from a large integrated health system to predict the onset of prediabetes, type 2 diabetes (T2D), and metabolic syndrome in pediatric cohorts.

**Results:**

The cohort included 49 517 children with overweight or obesity aged 2-18 (54.9% male, 73% Caucasian), with a median follow-up time of 7.5 years and mean body mass index (BMI) percentile of 88.6%. Our model demonstrated area under receiver operating characteristic curve (AUC) accuracies up to 0.87, 0.79, and 0.79 for predicting T2D, metabolic syndrome, and prediabetes, respectively. Whereas most risk calculators use only recently available data, incorporating longitudinal data improved AUCs by 13.04%, 11.48%, and 11.67% for T2D, syndrome, and prediabetes, respectively, versus models using the most recent BMI (*P *<* *2.2 × 10^–16^).

**Discussion:**

Despite most risk calculators using only the most recent data, incorporating longitudinal data improved the model accuracies because utilizing trajectories provides a more comprehensive characterization of the patient’s health history. Our interpretable model indicated that BMI trajectories were consistently identified as one of the most influential features for prediction, highlighting the advantages of incorporating longitudinal data when available.

## Introduction

The prevalence of childhood obesity has more than doubled since 1980, with an estimated global incidence of obesity among children and adolescents exceeding 124 million individuals. This trend is a growing concern for public health experts and policymakers, as these diseases have serious long-term consequences on health and well-being.^1–3^ Like adults, pediatric obesity increases the risk for developing a range of serious comorbidities including cancer, chronic liver disease, musculoskeletal, psychological, and sleep disorders.^4–8^ Depending on the definition applied, up to 39% of children with obesity present with metabolic syndrome.^1^ Metabolic syndrome is characterized by a combination of metabolic abnormalities related to glucose intolerance, insulin resistance, obesity, dyslipidemia, and hypertension. In addition to the risk posed by genetic predisposition, metabolic diseases are becoming more common in children due to combination of factors, including changes in diet, lifestyle, and environment.^9^^,^^10^ Early identification and intervention for metabolic diseases are essential for improving the long-term quality of life of affected children and mitigating the risk of additional comorbid conditions, such as type 2 diabetes (T2D). The current prevalence of metabolic syndrome, T2D, and prediabetes for adults is estimated to be 41.8%, 11.3%, and 38.0% for prediabetes in the United States.^11–13^ Despite the magnitude of this public health problem, no clinical tools are in widespread use to predict the onset of metabolic diseases in children.^14^^,^^15^

Electronic health records (EHRs) contain structured information about patients' medical history, treatments, and outcomes and have emerged a valuable resource with the potential to predict and prevent disease. However, the best way to utilize EHR data for disease prediction is still not well understood. Many methods for analyzing EHR data, including machine learning and other predictive modeling techniques continue to be explored.^16–18^ However, EHR data present with many challenges and potential for bias due to irregular time sampling, nonrandom missingness, among other challenges, requiring thoughtful model implementation.^19^^,^^20^ Notably, the majority of models in the literature do not make use of available longitudinal data in EHRs, instead relying on the most recent data,^21–23^ which may miss opportunities to improve predictions. Utilizing historical clinical data can provide a more comprehensive picture of a patient's health by capturing the fluctuation of physiological factors over time rather than relying solely on the most recent data. Deep learning (DL)^24^ is a type of machine learning that uses artificial neural networks^25^ as its underlying algorithm, and has gained popularity in recent years due to its ability to work with longitudinal or time series data for prediction purposes.^16^^,^^18^^,^^26^^,^^27^ In recent years, many DL architectures have shown promise for predicting disease risk utilizing data from EHRs.^16^^,^^18^^,^^28^^,^^29^ In addition to model accuracy, the earlier a model can identify the disease risk, the better the opportunities for intervention will be. Previous studies demonstrated that longitudinal data can improve risk estimation, such as blood glucose variability to predict the risk of microvascular complications in patients with type 2 diabetes (T2D).^30^^,^^31^ Furthermore, we recently performed a large-scale simulation study to identify which DL models perform best when utilizing longitudinal clinical lab data from the EHR, revealing time series forest-convolutional neural networks (TSF-CNN) as a highly robust architecture for this purpose.^16^

Though previous machine learning approaches have provided good prediction accuracies, their application in clinical settings has been limited in part because the decisions driving the predictions are opaque, creating concerns for implicit bias and an overall lack of trust.^32^ This lack of interpretability has thus far contributed to the limited use of these powerful and emerging methods into the provision of patient care.^32^ Interpretable methods can explain why a certain prediction was made for a patient, reducing this barrier.

This study aims to utilize interpretable deep learning models, incorporating longitudinal clinical measurements, demographical data, and diagnosis codes from EHR data to help address a growing health concern for pediatric onset of T2D, metabolic syndrome, and prediabetes. The study hypothesizes that the inclusion of longitudinal data will improve disease prediction accuracy compared to models using only the most recent data. The wide-and-deep framework employed combines discrete data (ICD codes, demographics) and longitudinal data (body mass index (BMI) trajectories) to enhance prediction accuracy, while the interpretable nature of the approach allows for investigating the reasons behind the model's predictions. The study's contributions lie in introducing interpretable deep learning models for disease prediction, utilizing an innovative model architecture that integrates longitudinal data from EHRs with discrete data, and addressing the lack of trust and interpretability associated with previous machine learning approaches.

## Methods

### Pediatric patient cohort

The cohort consisted of 49 517 pediatric patients (54.9% male and 45.1% female) seen at the Cleveland Clinic between January 2000 and August 2023 and had at least 2 height and weight records from different months. Structured data, including height, weight, demographic information, encounter diagnosis International Classification of Disease (ICD) 9/10 codes, and medication records, were extracted from the enterprise-wide EHR (Supplementary Tables S1-S3). Patients who had at least 1 BMI measurement above the threshold for overweight or obesity based on the Centers for Disease Control growth curves were included.^33^ To ensure patients included in the analysis were still active patients in the Cleveland Clinic EHR after the average age of disease onset, patients must have at least 1 visit every 3 years after the age of 12.

Patients were considered to have T2D based on the eMERGE criteria.^34^ Individuals were considered to have metabolic syndrome if: (1) a physician diagnosed the patient with either ICD-9 disease code 277.7 or ICD-10 disease code E88.81 between age of 2 and 18. (2) or based on the International Diabetes Federation criteria for metabolic syndrome (Supplementary Table S4).^35^ For the diagnosis of prediabetes, patients were identified using either (1) ICD-9 disease code 790.21, 790.22, 790.29 or ICD-10 disease code R73.01, R73.02, R73.03, R73.09, and R73.9 between age of 2 and 18. (2) or based on the standard glucose criteria for prediabetes.^36^

**Figure 1.**
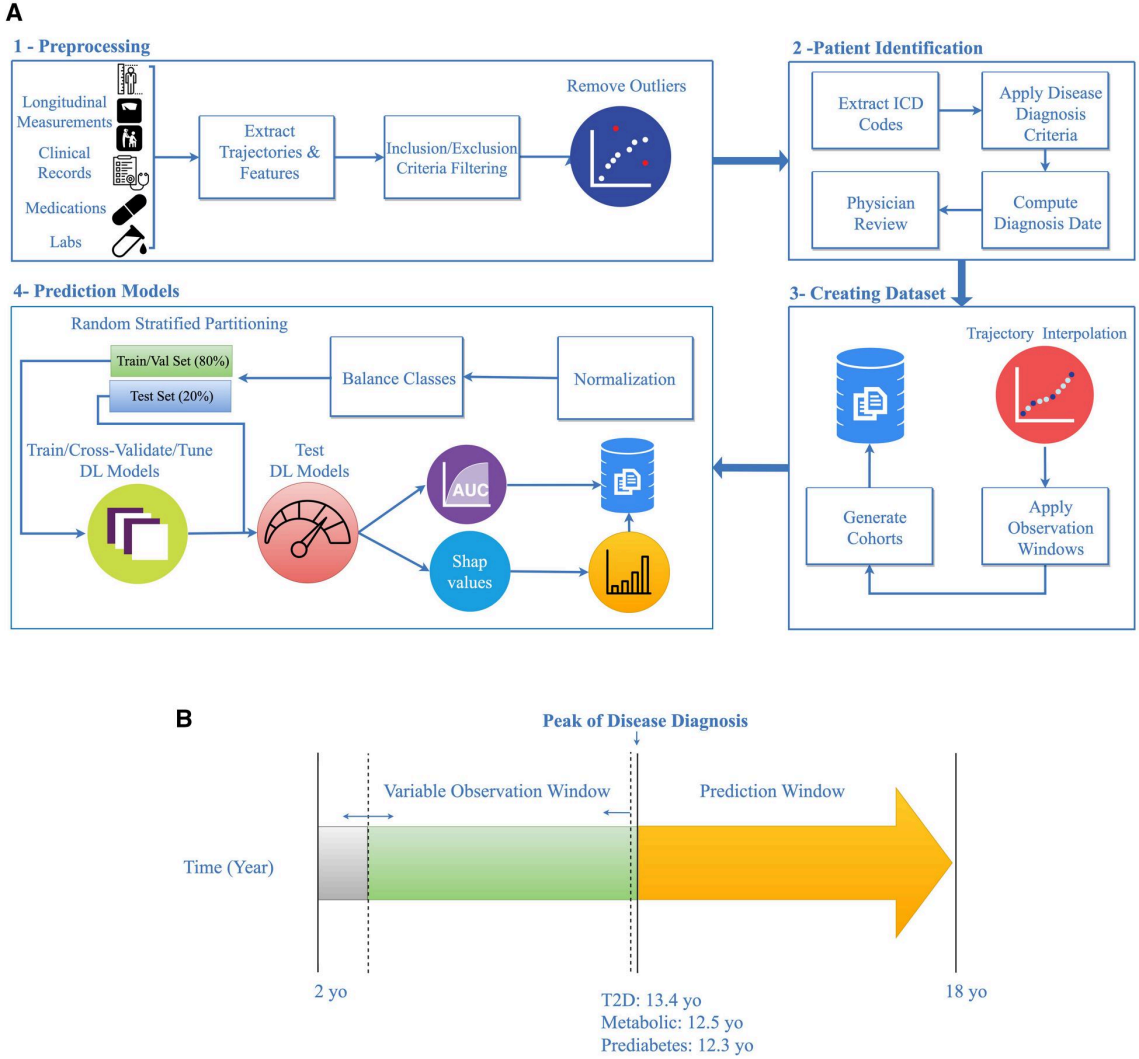
Workflow for modeling pediatric metabolic cohorts. (A) Data such as weight, height, clinical records, medications, and lab records were extracted from the EHR. Height and weight outliers were removed, and patients with <2 BMI records were excluded. Next, patients were labeled as underweight, normal weight, having overweight, having obesity, and having severe obesity according to Centers for Disease Control criteria, and were classified either developing one of targeted outcomes or not. Trajectories were then harmonized to consist of a single BMI record per year. Trajectories were truncated to exclude any records at or after the average age of disease onset or any records after a diagnosis of the outcome. Because deep learning models require uniform trajectory lengths, multiple datasets were created across a variety of different age ranges. For each dataset, a DW-TSF-CNN model was tuned, trained on 80% of data, evaluated by 3-fold cross-validation and tested on 20% of the withheld dataset. The impact of each feature on the model decision was calculated using SHapley Additive exPlanations (SHAP). (B) The observation and prediction window definitions. The observation window varied from 2 years old to the average age of each disease in pediatric patients (13.4 years old for T2D, 12.5 years old for metabolic syndrome, and 12.3 for prediabetes), and the prediction window was fixed from average of disease diagnosis to 18 years old.

For each patient, an observation and a prediction window were established. The observation window is where the data were obtained for prediction and the prediction window is the time frame that the model was evaluating for the risk of disease. The observation window for controls was defined as the period from 2 years old to the average age of disease diagnosis in the cohort. The observation window for cases was defined from 2 years old to the date of disease diagnosis or the average age of disease diagnosis, whichever came first. The prediction window for both case and control cohorts was defined from the average age of disease diagnosis to 18 years old (Figure 1). Patients have clinical histories of different lengths and have visits at different intervals. We addressed the limitation of different lengths of trajectories by partitioning the data into multiple cohorts with different observation windows (Figure 1). The max age for the observation windows used for model training was based on the average age of onset of the target diagnosis—13.4 years for T2D, 12.5 years for metabolic syndrome, and 12.3 for prediabetes.

We trained models on data collected prior to the average age of onset to ensure that the models would be useful for age ranges prior to when most pediatric patients are diagnosed, providing insight into real-world clinical utility. For each cohort, data extracted during the observation window were used to train a classifier to predict whether the individual would be diagnosed with the disorder in the subsequent prediction window. We defined cases as individuals with ≥1 target disease diagnosis anytime between 2 years old and 18 years old. Controls were individuals that did not meet the criteria for the target disorder from the age of 2 to 18 years old. Both cases and controls must also meet the active patient criterion, as described above, to ensure they were still being seen by providers in the Cleveland Clinic health system. Additional selection criteria were implemented based on data availability and are described in the Data Processing and Quality Control section below. This study was approved by Cleveland Clinic Institutional Review Board (IRB #20-1035) and complied with current ethical regulations. The workflow for cohort selection and data processing is depicted in Figure 1.

### Feature extraction and data processing of longitudinal features

Feature extraction is the process of choosing variables that are useful for predicting the outcome, and we considered both longitudinal and scalar features. Longitudinal features included BMI trajectories and mean number of visits per year. The process for extracting and processing BMI trajectories has been previously described.^16^ Briefly, because this model architecture requires a uniform matrix (ie, same number of longitudinal data points across all patients), we partitioned the BMI trajectories into disjoint 1-year segments, and each segment was assigned a BMI based on the mean of all BMIs recorded within the segment. For BMI trajectories, linear interpolation was used to impute missing records between the first record and the last available record in the trajectory. Patients without a BMI trajectory covering the observation window were excluded. Visit trajectories were calculated based on the number of unique days each patient had an encounter visit in the EHR. We then truncated trajectories to exclude any records at or after the average age of the target outcome. We also incorporated additional features based on the results from association tests using logistic regression association tests. Features associated with outcomes (FDR *P* < .05) that remained after least absolute shrinkage and selection operator (LASSO) with lambda of 1 SD (Supplementary Tables S5-S8) were included.

### Model training

Cohorts were randomly partitioned into training/validation (80%) and test sets (20%), respectively. Next, the training and test sets were Z-score normalized prior to model training. We randomly selected 20% of the training set to perform hyperparameter tuning for each model. We considered 4 performance metrics to evaluate model performance: (1) mean cross-validation area under receiver operating characteristic curve (AUC) in the training set, (2) AUC in the test set, (3) sensitivity, and (4) specificity in the test set.

**Figure 2.**
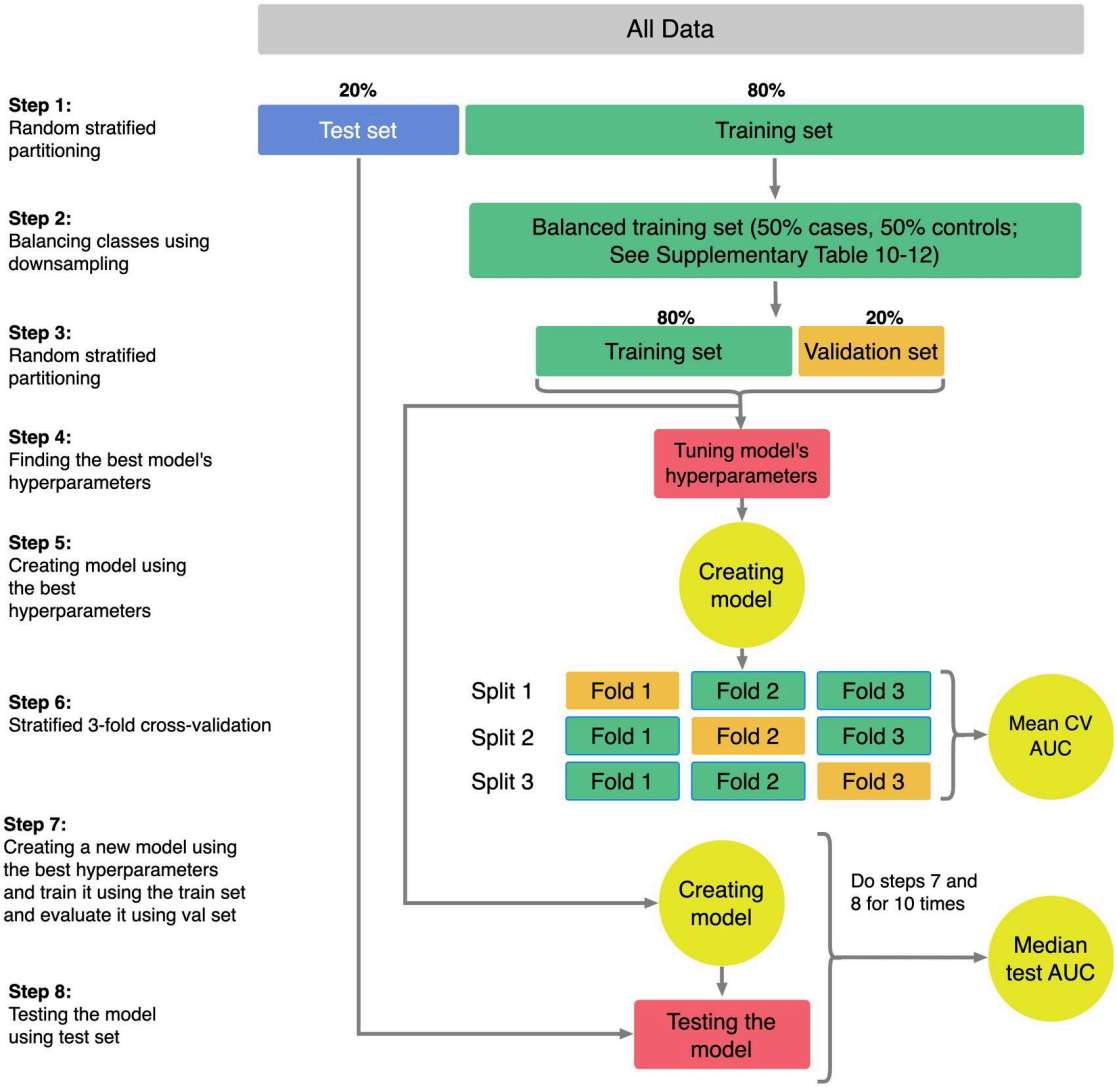
Model training strategy using training, testing, and validation data sets. An 8 step process was performed to develop and validate models that were robust to model overfitting.

The number of cases (individuals who have the target outcome) is a relatively small fraction of the cohort, and we maintained the proportion of cases and controls in the training and test sets using a balanced (stratified split) strategy. Cohorts with fewer than 20 patients were removed due to insufficient sample size for model training.^37^ To encourage accurate and realistic validation, we performed stratified 3-fold cross-validation, ensuring that each fold had approximately the same proportion of target cases as the full input, and we reported the mean cross-validation AUC. Models were created based on optimized hyperparameters, trained using the training set, and evaluated using the test set. Similarly to Zhao et al.,^18^ the process of training and testing was completed in 10 iterations. For each iteration, we calculated the AUC, sensitivity, and specificity after applying the model on the withheld test set. We used the median value for each metric in our results to have robust performance metrics. Figure 2 shows the details of how the training and validation process were performed.

### Prediction of metabolic diseases using longitudinal trajectories and clinical features (DW-TSF-CNN)

We adopted a wide-and-deep approach to jointly train wide linear models and deep neural networks, combining the benefits of memorization and generalization for recommender systems.^38^ The originally proposed wide-and-deep model consisted of a multilayer perceptron in the deep component and a logistic regression model in the wide component. Here, we extended this approach to incorporate a deep TSF-CNN model that accommodates multivariate longitudinal trajectories and discrete values (wide) to make a deep-and-wide TSF-CNN (DW-TSF-CNN) architecture. We initialized the learning rate, batch size, and number of epochs of 0.001, 32, and 500, respectively. For the DW-TSF-CNN model, the Adam optimization approach was used with the categorical cross-entropy cost function. The learning rate was reduced by an arbitrary factor of 0.92 each time the model’s training loss was unimproved for 3 consecutive epochs. Although the learning process for each classifier was permitted to take up to 500 iterations, a stopping criterion was implemented across all models, and training was stopped if no improvement in model accuracy was gained after 15 consecutive iterations. Details regarding system specifications are provided in the Supplementary material.

We incorporated SHAP (SHapley Additive exPlanations) values^39^ into our model to enhance interpretability. SHAP values provide valuable insight into the contribution of each feature toward the model's predictions by evaluating the effect of including/excluding each specific feature on the prediction. This is put into context by considering all possible combinations of features and calculating the average contribution of each feature across different permutations. The highest absolute SHAP value from each patient’s trajectory was used as a measure of feature importance for the patient trajectory.

We compared the DW-TSF-CNN approach with Random Forest, a traditional machine learning method, as well as convolutional recurrent neural network (CRNN) and Transformer models. CRNN (Supplementary Figure S1) ranked best among RNN models in our comprehensive study of finding a robust DL model for processing longitudinal EHR data,^16^ while Transformer (Supplementary Figure S2) is a widely recognized large language model known for its exceptional capabilities in analyzing and processing complex textual data. The Transformer is a multihead attention model, and the CRNN is a feed-forward network. A residual connection is attached to each sublayers followed by layer normalization and a dropout to reduce the output tensor size. The number of attention heads, head size, dropout amount, and the number of neurons of the feed-forward model are determined in the hyperparameter tuning process (eg, number of heads, ff size, head size, drop out). The model architectures for these are shown in Supplementary Figures S1 and S2, and the hyperparameter tuning parameters are shown in Supplementary Table S9.

### Prediction of metabolic diseases using BMI trajectories only (TSF-CNN) compared to the most recent BMI (logistic regression)

To compare whether models utilizing longitudinal BMI trajectories outperform those using only the most recent BMI, we implemented a TSF-CNN for predicting using BMI trajectories and logistic regression for the most recent BMI (a deep learning model with a single predictor reduces to logistic regression).

## Results

### Type 2 diabetes

**Figure 3.**
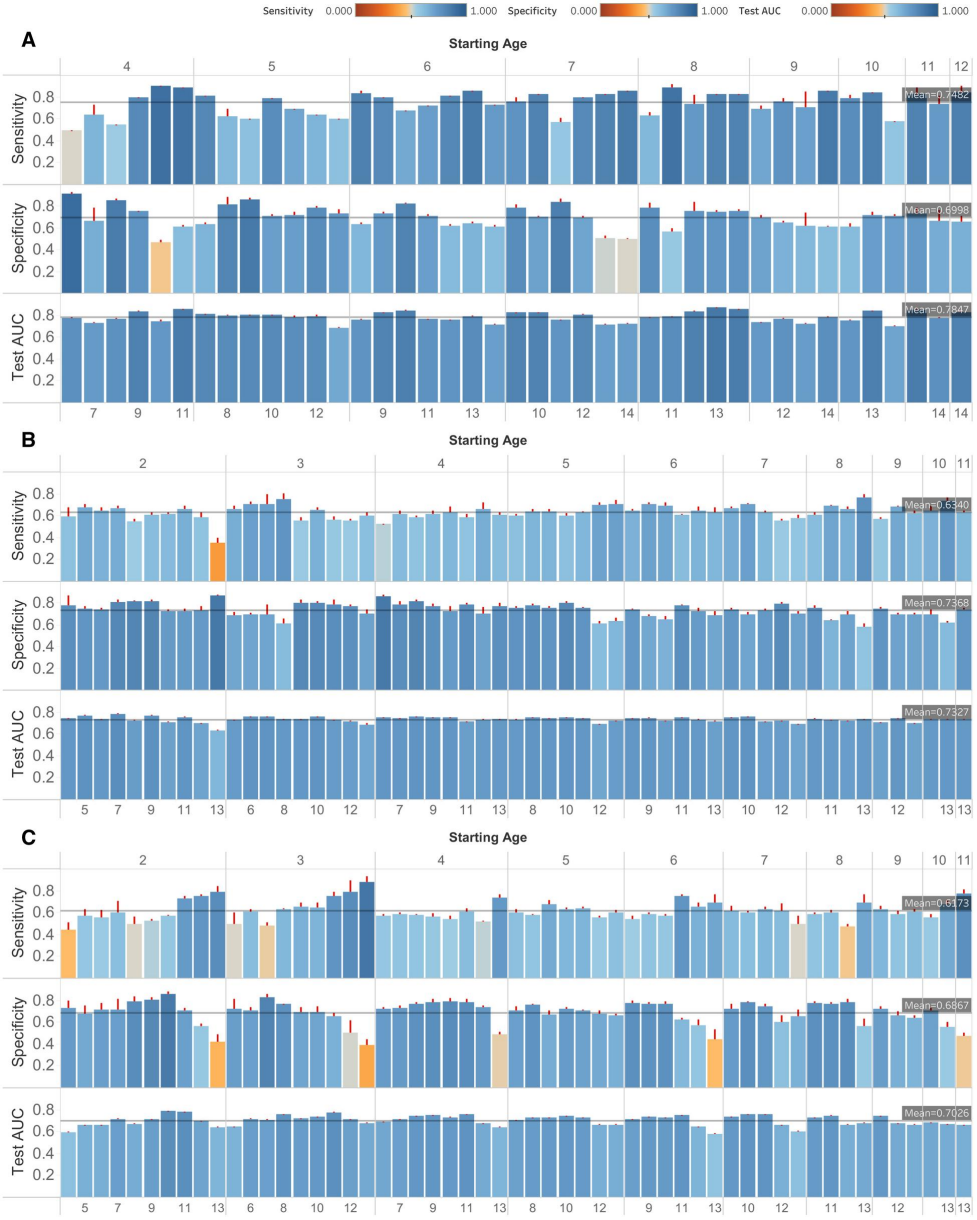
DW-TSF-CNN model performance across various age ranges and length of data histories. Test area under the receiver operating characteristic curve (AUC), specificity, and sensitivity of DW-TSF-CNN across different (A) type 2 diabetes (T2D); (B) prediabetes; and (C) metabolic syndrome cohorts. Starting age groups of 2 and 3 years were excluded in the T2D cohort because they did not meet the criteria of having a minimum sample size of 20 patients.

A total of 28 143 pediatric patients met the inclusion criteria for the cohort to train the T2D predictive model (Supplementary Figure S3). The final cohort was 45.82% female, with an average age of 13.40 (SD = 3.22) years at the T2D diagnosis. A total of 356 (1.26%) patients met the criteria for T2D according to the eMERGE criteria.^34^ We investigated 66 observation windows for predicting T2D depending on the extent of patient data available (Supplementary Table S1). The average sensitivity, specificity, and test AUC across all cohorts were 0.75, 0.70, and 0.78, respectively (Figure 3A).

### Prediabetes

A total of 29 198 pediatric patients met the inclusion criteria for the cohort to train the prediabetes predictive model (Supplementary Figure S3). The cohort was 45.8% female, with an average age of 12.32 (SD = 3.65) years at diagnosis. A total of 3120 out of 29 198 patients (10.7%) met the criteria for prediabetes. We investigated 55 different observation windows for predicting prediabetes depending on the extent of patient data available (Supplementary Table S2). The average sensitivity, specificity, and test AUC across all cohorts were 0.63, 0.74, and 0.73, respectively (Figure 3B).

### Metabolic syndrome

A total of 29 139 pediatric patients met the inclusion criteria for the cohort to train the metabolic syndrome predictive model based on their available EHR data (Supplementary Figure S3). The final cohort was 45.9% female, with an average age of 12.54 (SD = 2.65) years at the time of diagnosis. A total of 5462 out of 29 139 patients (18.7%) met the metabolic syndrome criteria and were labeled as positive cases. We investigated 55 age-based observation windows depending on the extent of patient data available (Supplementary Table S3). The average sensitivity, specificity, and test AUC across all cohorts were 0.62, 0.69, and 0.70, respectively (Figure 3C).

### Clinical features driving predictions of metabolic diseases

**Figure 4.**
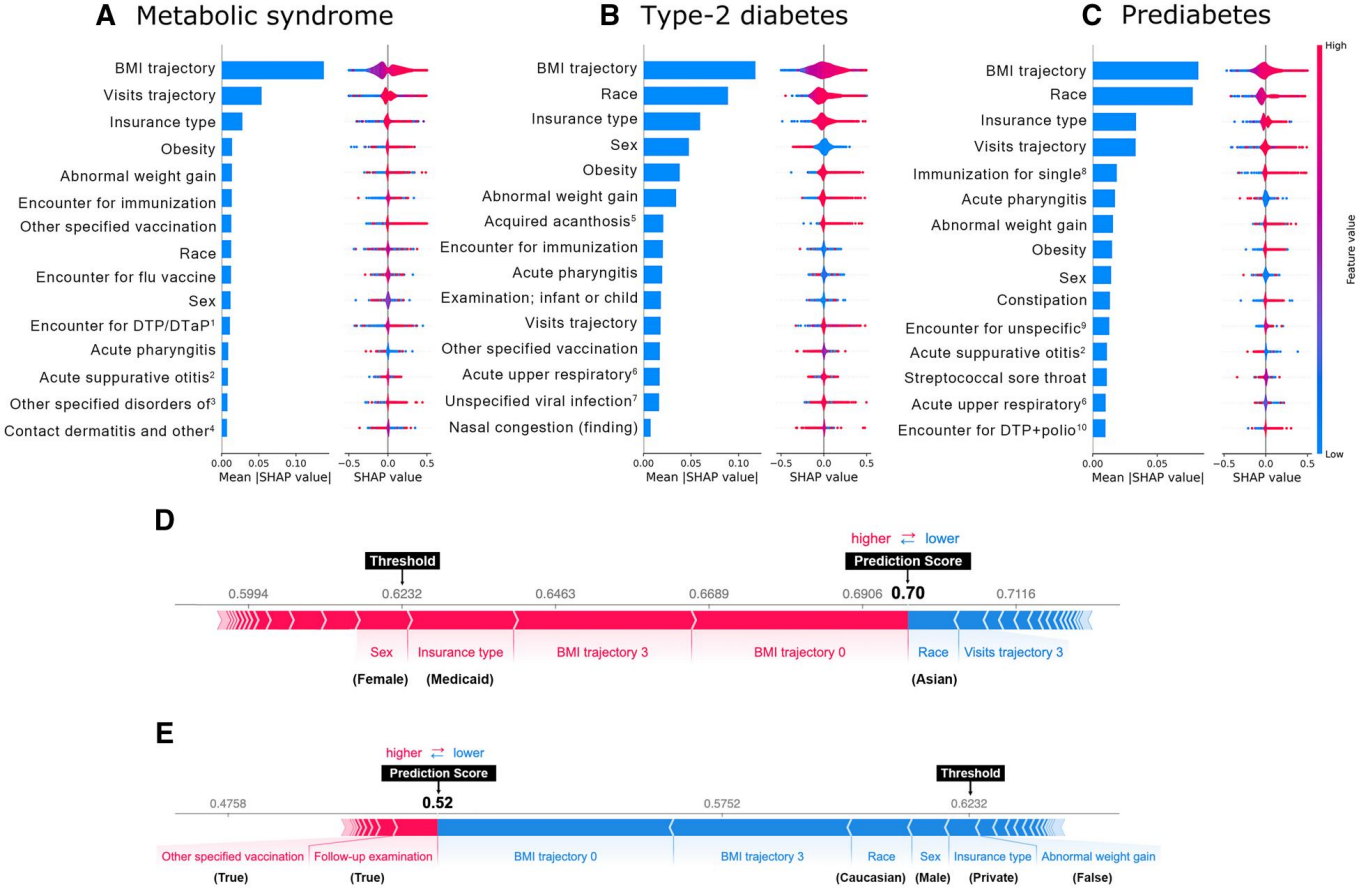
Interpretations of clinical inputs driving model decisions. (A) Fifteen most important features, based on SHAP values from the DW-TSF-CNN model for predicting metabolic syndrome. (B) Fifteen most important features, based on SHAP values from the DW-TSF-CNN model for type 2 diabetes (T2D). (C) Fifteen most important features, based on SHAP values from the DW-TSF-CNN model for predicting prediabetes. In each subplot (A-C), the left side of the plot represents global feature importance, calculated as the mean absolute value for that feature over all the given samples over all cohorts which cover different age ranges. BMI values were normalized using Z-scores, resulting in BMI trajectories that range from a negative to a positive number. As a result, patients with low BMI are represented by blue dots, while high BMI are represented by red dots, and average BMI values are represented by purple dots. The right side of the plot shows dots with 2 characteristics: (i) Color shows whether that feature was high or low (for noncategorical features), and (ii) horizontal location shows whether the effect of that value caused an increased or decreased likelihood of a positive prediction. The plot for the BMI trajectory indicated that the individuals with lower or average BMI trajectory (blue and purple dots) always had a lower SHAP value which means they always decreased the likelihood of getting a positive prediction for the outcome while the abnormal BMI trajectory (red dots), in the most cases, increased the likelihood of getting a positive prediction for the outcome. Two correctly classified metabolic syndrome cases, with data from age 8-13 years old, are shown with the reason the model made its prediction. (D) A positive prediction was made because the patient has high risk features in their BMI trajectory (positions 0 and 3 representing age of 8 and 11), being female, and socioeconomic risk factors (eg, Medicaid insurance). This patient had a 0.70 probability of developing metabolic syndrome, which was higher than the model threshold of 0.62, resulting a true positive prediction. (E) A negative prediction was made because the patient has reduced risk due to being male, features in their BMI trajectory, using a private insurance, and no history of abnormal weight gain. Despite the increased risk due to socioeconomic factors, this patient had a 0.52 probability of developing metabolic syndrome, less than the optimized model threshold of 0.6232, resulting in true negative prediction.

The 15 most important features for predicting pediatric T2D, prediabetes, and metabolic syndrome are shown in Figure 4A-C. For each feature and outcome, SHAP values, representing feature importance, were calculated for age-based observation windows.

BMI trajectories were identified as the most important feature for predicting whether a patient will develop metabolic syndrome, T2D, or prediabetes. Longitudinal trends in visits were considered the second most important predictor for metabolic syndrome, and the one of most important features for T2D and prediabetes. Additionally, race, insurance type, sex, obesity diagnosis, and diagnosis of unusual weight gain were also ranked as important predictors. The same approach was applied to extract top features for random forest, which are shown in Supplementary Figure S4. Again, BMI trajectories were identified as the most significant predictor of an individual’s susceptibility to developing metabolic syndrome, T2D, or prediabetes using random forest. Other features that impacted the prediction of these outcomes in both modeling approaches included race, sex, insurance type, diagnosis of obesity, and vaccination status (Supplementary Figure S4).

SHapley Additive exPlanations values provide an opportunity to deconstruct a prediction into the sum of effects for each feature. We subsequently demonstrated the utility of this approach by showing the weight of features that contributed to a positive prediction and a negative prediction for whether a patient will develop metabolic syndrome (Figure 4D and E). In these examples, the model correctly predicted a female patient would develop metabolic syndrome with a model score of 0.70, which exceeded the model threshold required for a positive prediction of 0.62. This decision was made based on the first and the fourth time point of the BMI trajectory, sex, and insurance type, while race and fourth time point of the visit trajectory were the major features that pushed the model score higher (Figure 4D). In a second example, a male patient was correctly predicted that they would not develop metabolic syndrome based on a model score of 0.52, which was under the required threshold of 0.62. This decision was driven by the first and fourth time point of the BMI trajectory, race, sex, insurance type, and no diagnosis of abnormal weight gain, which reduced the model score, while diagnosis for follow-up examination and other specified vaccination were the primary features that increased the model score (Figure 4E).

### Comparison of DL with other modeling approaches

#### Comparing the use of longitudinal BMI histories in deep learning compared to other machine learning approaches

**Figure 5.**
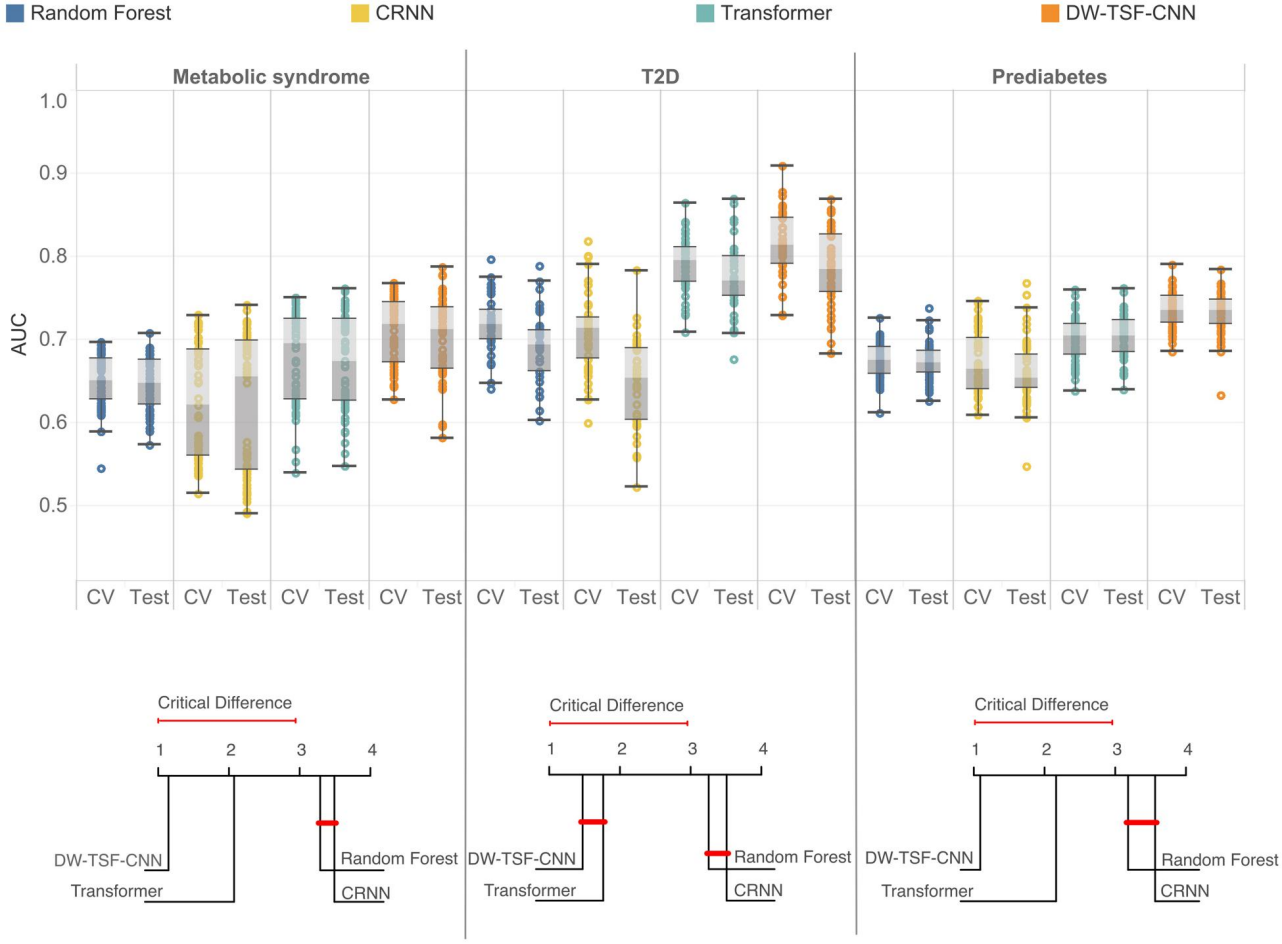
DW-TSF-CNN (A) Cross-validation and test AUCs from the random forest, CRNN, Transformer, and DW-TSF-CNN, models across all age ranges for each disease. (B) Critical difference plot to compare model's performance based on their test AUC (lower value is better).

We tested whether the DW-TSF-CNN approach outperformed all other models including random forest, CRNN, and Transformer based on the AUC in the withheld test set and the mean cross-validation AUC, sensitivity, and specificity for each observation window. Overall, a substantial difference in model performance was observed. DW-TSF-CNN had median test AUCs of 0.71 (range: 0.58-0.79), 0.78 (0.68-0.87), and 0.74 (0.69-0.78) for metabolic syndrome, T2D, and prediabetes, respectively (Figure 5). The second-best performing model was Transformer with median test AUCs of 0.67 (0.55-0.76), 0.77 (0.71-0.87), and 0.71 (0.64-0.76) for metabolic syndrome, T2D, and prediabetes, respectively. Random forest had median test AUCs of 0.65 (0.57-0.71), 0.69 (0.60-0.77), and 0.67 (0.63-0.72) for metabolic syndrome, T2D, and prediabetes, respectively. Similarly, CRNN had median test AUCs of 0.66 (0.50-0.74), 0.65 (0.52-0.78), and 0.65 (0.60-0.74) for metabolic syndrome, T2D, and prediabetes, respectively.

Overall model accuracy was determined using a Friedman test to evaluate model test AUCs across all age ranges for each disease and was depicted as a critical difference plot (Figure 5). DW-TSF-CNN was ranked as the best model for all diseases.

#### Comparing the use of longitudinal BMI histories to using only the most recent BMI

**Figure 6.**
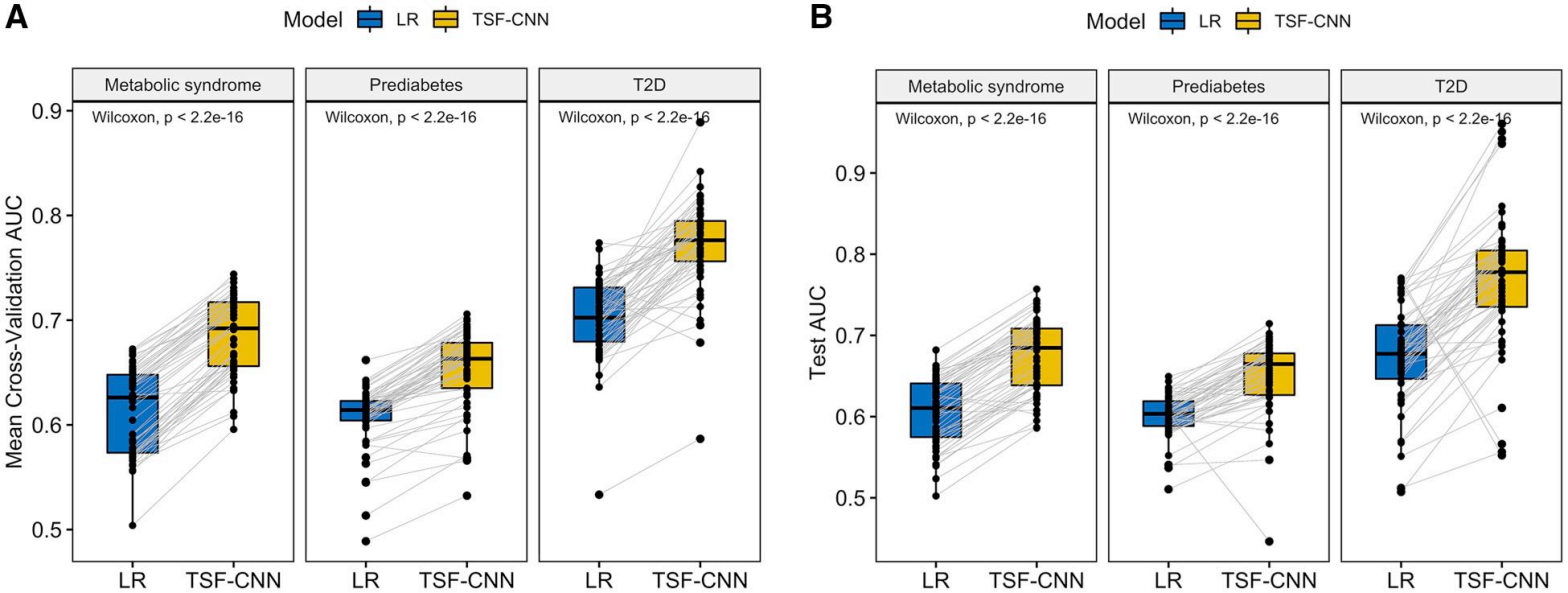
Evaluation of whether use of longitudinal BMI data (TSF-CNN model) improves prediction over utilizing only the most recent BMI (LR: logistic regression model) based on (A) mean cross-validation AUC and (B) the test AUC.

We investigated the impact of leveraging longitudinal BMI data compared to the common practice of only utilizing the most recent BMI. The AUC from the withheld test set and the cross-validation AUC were used to evaluate the predictive performance of each model. Overall, a substantial difference in model performance was observed. TSF-CNN, used to predict using only the longitudinal BMI history, had median test AUC of 0.68 (ranging from 0.58 to 0.77), 0.67 (0.56 to 0.71), and 0.78 (0.68 to 0.86) for metabolic syndrome, prediabetes, and T2D, respectively (Figure 6). The logistic regression model, used to predict using only the most recent BMI, had test AUC of 0.61 (ranging from 0.50-0.69), 0.60 (0.55-0.66), and 0.69 (0.55-0.78) for metabolic syndrome, prediabetes, and T2D, respectively. The increased predictive performance of the longitudinal BMI trajectories was significantly better than using only the most recent BMI (*P *<* *2.2 × 10^−16^) of 13.04%, 11.48%, and 11.67% for T2D, metabolic syndrome, and prediabetes, respectively.

## Discussion

The increasing incidence of childhood obesity in the US and worldwide is dramatic and persists as one of the most challenging health problems.^1^ This trend is expected to result in a substantial increase in obesity-related commodities such as T2D, metabolic syndrome, among many others.^9^

To this end, EHRs are leading to a rapid increase in diverse longitudinal clinical data, which is a valuable tool for population-level analyses to identify trends within patient records that can predict disease. As more detailed longitudinal data (eg, wearables) becomes available, the opportunities for early detection of disease, reducing avoidable care, and providing clinical decision support increase. Although substantial research has been done to predict metabolic disease in adult patients, studies to predict pediatric metabolic diseases are much more limited. A previous study in 1080 participants demonstrated that physical measurements (eg, waist-to-height) from a single time point, T2D family history and acanthosis nigricans were correlated with the occurrence of metabolic syndrome or prediabetes in a group of participants including children and adolescents with obesity.^40^ A recent study highlighted the importance of early intervention in pediatric obesity, as it can prevent the progression of the disease into adulthood, and has emphasized the need for more research in the field of precision medicine in obesity management, including the development of biomarkers for predicting treatment response and the implementation of effective prevention strategies.^14^ The authors argued that precision medicine approaches may be able to help identify subgroups of patients with different underlying mechanisms of obesity that may have a modified response to certain treatment options. Here, we significantly extend these studies by utilizing an AI platform that leverages BMI and mean number of visits per year as longitudinal clinical data from an EHR to predict key metabolic diseases in pediatric patients. Although height and weight are typically recorded separately in the clinic to monitor the health status of growing children and adolescents, tracking the trajectory of BMI values has proven to be useful for assessing children's health.^41^ Many studies have shown that BMI in early childhood is an important predictor of T2D and prediabetes,^40^^,^^42–45^ and our previous work demonstrated that BMI trajectories alone can predict pediatric T2D onset with an AUC of up to 0.72.^16^ However, by incorporating additional data such as ICD codes, sex, race, insurance type, and measures of healthcare utilization (eg, mean number of visits per year), we were able to increase the AUC for predicting pediatric T2D up to 0.87 (Figure 5).

Our proposed model (DW-TSF-CNN) outperformed all models on the same data for T2D, prediabetes, and metabolic syndrome (Figure 5).

Furthermore, incorporating longitudinal trajectories added value compared to using only the most recent BMI, which is commonly used in risk calculators^18^^,^^43^ (Figure 6). Utilizing the whole trajectory provides a more comprehensive characterization of the patient’s health history. The wide-and-deep architecture has the benefit of jointly training models using both deep (longitudinal) and wide (scalar) features. Information extracted from the longitudinal trajectory can provide important risk factors such as weight variability, which has been identified as a risk factor for cardiovascular disease.^46–50^ Here, we also observed an increase in performance using the longitudinal features, based on the models using longitudinal BMI trajectories alone outperforming models using only the most recent BMI (Figure 6). For example, the visits trajectory demonstrated that pediatric patients that had more frequent contact with the healthcare system were less likely to develop these disorders. Importantly, model accuracies were reasonably stable across different time windows, indicating that accurate predictions may be possible for patients across a wide range of available data (Figure 3).

Our explainable AI approach allows for improved interpretation of what patient features led the model to make its prediction, improving transparency (Figure 4). This provides an important advantage over previous approaches where the decisions driving the prediction were a “black box.” This inhibits trust in the model and creates a barrier to clinical adoption. Here, the ability to see what features are behind the predictions not only creates trust but also gives key insight into potentially modifiable risk factors for these metabolic diseases. BMI trajectories were consistently considered the first important feature, highlighting the value of the longitudinal information but also the potential importance of identifying and managing obesity to prevent the onset of these pediatric disorders that will have significant impacts on quality of life for many years. Notably, encounter for flu vaccine and other immunizations were considered important predictors and were negatively associated with risk of metabolic disease. These features are thought to represent patients who have increased access to the healthcare system for preventative measures, which would subsequently enable earlier detection, intervention, and monitoring of health conditions.

Race contributed significantly to model predictions, with the largest impact on T2D and prediabetes predictions (Figure 4). We investigated the influence of race on disease development in the DW-TSF-CNN model in more detail (Supplementary Figure S6) and found that Caucasian individuals displayed a decreased likelihood of a positive prediction and individuals that identified as Black were an increased likelihood of receiving a diagnosis. We also investigated model performance by race in Supplementary Figure S7.

As with any study, there were limitations with our design that present opportunities for future studies. The proposed model predicts whether patients will develop the disorder prior to the age of 18, but cannot predict the likely age of onset. This is difficult due to the need for uniform trajectory lengths, but this serves as an opportunity for enhancement. We emphasize the practical value of predicting outcomes at any point in time. However, due to data constraints, our analysis is focused on predictions prior to the age of 18. While the American Academy of Pediatric recommends universal screening for dyslipidemia for all children between 9 and 11 years and again between 17 and 21 years, other clinical measurements that may serve as excellent predictors for identifying metabolic syndrome (eg, fasting plasma glucose) are not routinely collected and are often only tested when the provider already suspects an underlying condition is present,^51^ and therefore were not included here. Furthermore, medication usage is relatively sparse in this age range, so its utility was limited in this context. Although not used as predictors, medications, and laboratory results, in addition to ICD codes, were used to identify patients as having the metabolic conditions in this study. Additional work may be necessary to identify appropriate methods that can overcome the limitations of incorporating laboratory test results and medications in this age range to improve predictions. Other data, such as insurance type, were recorded at baseline, and potential changes to insurance type were not accounted for. Recent research has indicated that including the temporal patterns of diagnosis codes can reveal the identification of recurring patterns, seasonal variations, or long-term changes in disease prevalence.^52^ Although the best way to incorporate these temporal data is an open area of research, this information may provide predictors beyond the presence or absence of an ICD code. In the future, we plan to investigate whether the inclusion of the temporal pattern of ICD codes can improve predictive performance. In addition, the data used in this study originated from a single, large integrated health system. Future evaluations may benefit from using data across multiple health systems to ensure the results are broadly applicable. In our analysis, we did not exclude individuals with any of the other 2 diseases from the control groups. While we acknowledge the potential shared features among these conditions, our decision was motivated by the aim of assessing the model performance in a realistic clinical setting, where individuals may have comorbid conditions. Future studies should consider the impact of the prediction accuracies in control groups without any history of related metabolic conditions.

In conclusion, we established a cutting-edge AI platform that yields more precise prediction of important metabolic disorders that are rapidly increasingly in pediatric populations. Furthermore, it highlights the value of incorporating longitudinal data when available to obtain a more comprehensive assessment of the patient’s health. This approach provides personalized risk prediction that could help physicians identify which patients are at greatest risk, and the explainable AI approach here could identify opportunities for interventions and increase trust in its implementation.

## Supplementary Material

ocae049_Supplementary_Data

## Data Availability

Data may be made available upon reasonable request and with appropriate review by the Cleveland Clinic Law Department.
