# Supplementary material for: An interpretable predictive deep learning platform for pediatric metabolic diseases
Source: J Am Med Inform Assoc. 2024 Mar 18;31(6):1227–38. doi: 10.1093/jamia/ocae049 (PMC11105121; doi:10.1093/jamia/ocae049)
Supplement: ocae049_Supplementary_Data [file ocae049_supplementary_data.docx]

Supplementary Information

An Interpretable Predictive Deep Learning Platform for Pediatric Metabolic Diseases

Hamed Javidi^1,2,3^, MS, Arshiya Mariam^1,3^, BS, Lina Alkhaled^3,4^, MD, Kevin M. Pantalone^3,4^, DO, Daniel M. Rotroff^1,2,3,4,#^, PhD

1. Department of Quantitative Health Sciences, Lerner Research Institute, Cleveland Clinic, Cleveland, OH, USA

2. Department of Electrical Engineering and Computer Science, Cleveland State University, Cleveland, OH, USA

3. Center for Quantitative Metabolic Research, Cleveland Clinic, Cleveland, OH, USA

4. Department of Endocrinology, Diabetes and Metabolism, Cleveland Clinic, Cleveland, OH, USA

**# Corresponding author**

Daniel M. Rotroff, PhD, MSPH

Department of Quantitative Health Sciences

Lerner Research Institute

Endocrinology and Metabolism Institute

Cleveland Clinic

9500 Euclid Avenue,

JJN3-01,

Cleveland, OH 44195, USA

Email: rotrofd@ccf.org

Phone: 216-444-3399

Keywords: Interpretable machine learning, Deep learning, Pediatric disease prediction, Type 2 diabetes, Longitudinal data, Electronic Health Record (EHR)

# Supplementary Methods

## Deep Learning Approach

We adopted a wide-and-deep approach to jointly train wide linear models and deep neural networks, combining the benefits of memorization and generalization for recommender systems.[1] The originally proposed wide-and-deep model consisted of a multi-layer perceptron (MLP) in the deep component and a logistic regression model in the wide component. Here, we extended this approach to incorporate a deep TSF-CNN model (DW-TSF-CNN). TSF-CNN was identified as a highly robust architecture for prediction using longitudinal data in our previous study (Supplementary Figure 5).[2] TSF-CNN leverages features engineered by the TSF algorithm and provided to a CNN with three, one dimensional convolution layers with 32 filters and with filter lengths of three linked to a ReLU activation function as the default hyperparameters. A max pooling layer with a stride of two was implemented after the second layer, which takes the maximum from each set of two values of the input. Then a GAP layer that receives the output of the sixth layer, performs averaging over the entire time dimension, feeding into a flat layer (Supplementary Figure 5). The wide component was a generalized linear model of the form *y = wT x + b*. y was the prediction variable, *x* = [*x1, x2, …, xd*] was a vector of d features, *w* = [*w1, w2, …, wd*] were the model parameters and b was the bias.

In the last layer, the wide and deep components were combined using a weighted sum of their output log odds as the prediction, which was then provided to a common logistic loss function for joint training. Joint training of a wide-and-deep model is done by backpropagating the gradients from the output to both the wide and deep part of the model simultaneously using mini-batch stochastic optimization. DW-TSF-CNN was then evaluated in a real-world pediatric (ages 2-18 years) cohort to predict onsets of pediatric T2D, prediabetes, and metabolic syndrome.


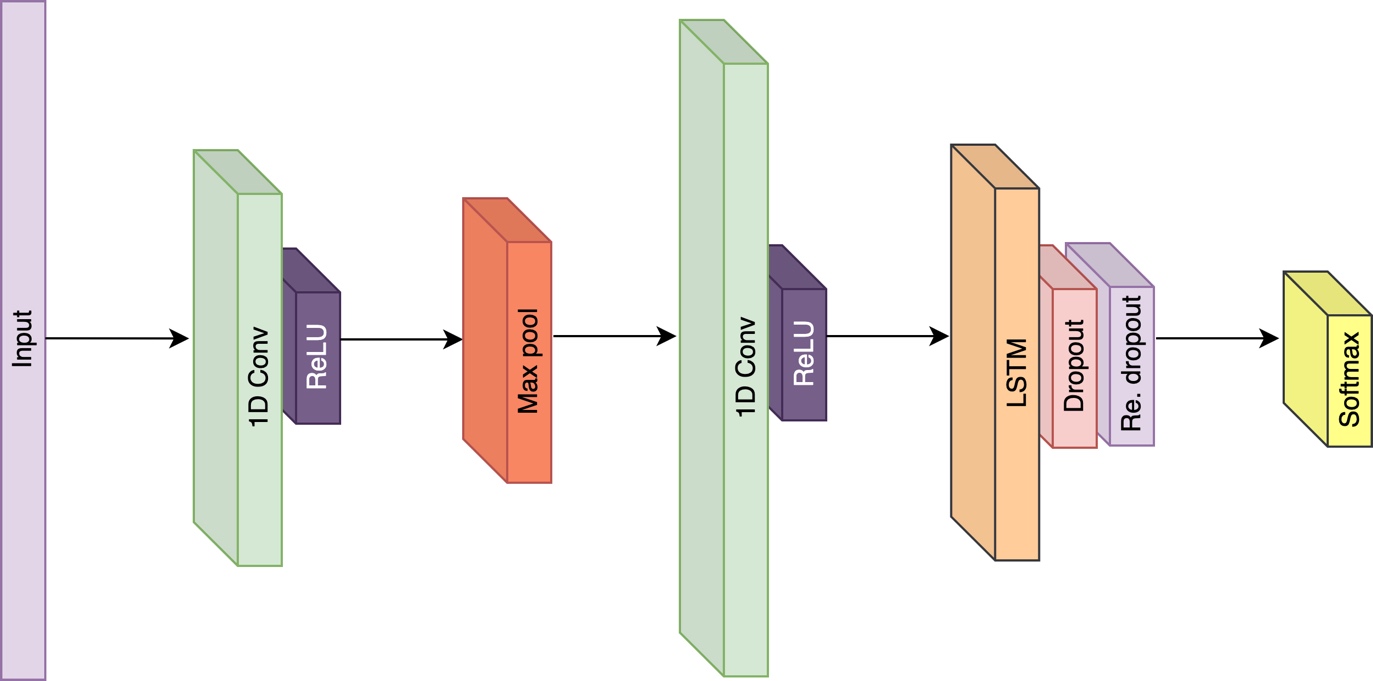


**Supplementary Figure 1.** Convolutional-Recurrent Neural Network architecture (CRNN)


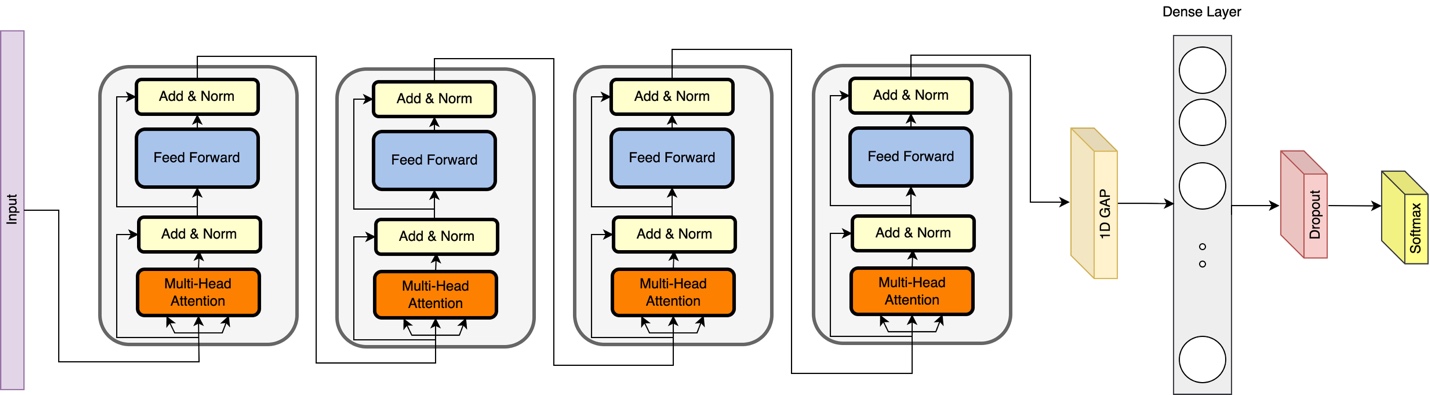


**Supplementary Figure 2.** Modified Transformer architecture for time series classification


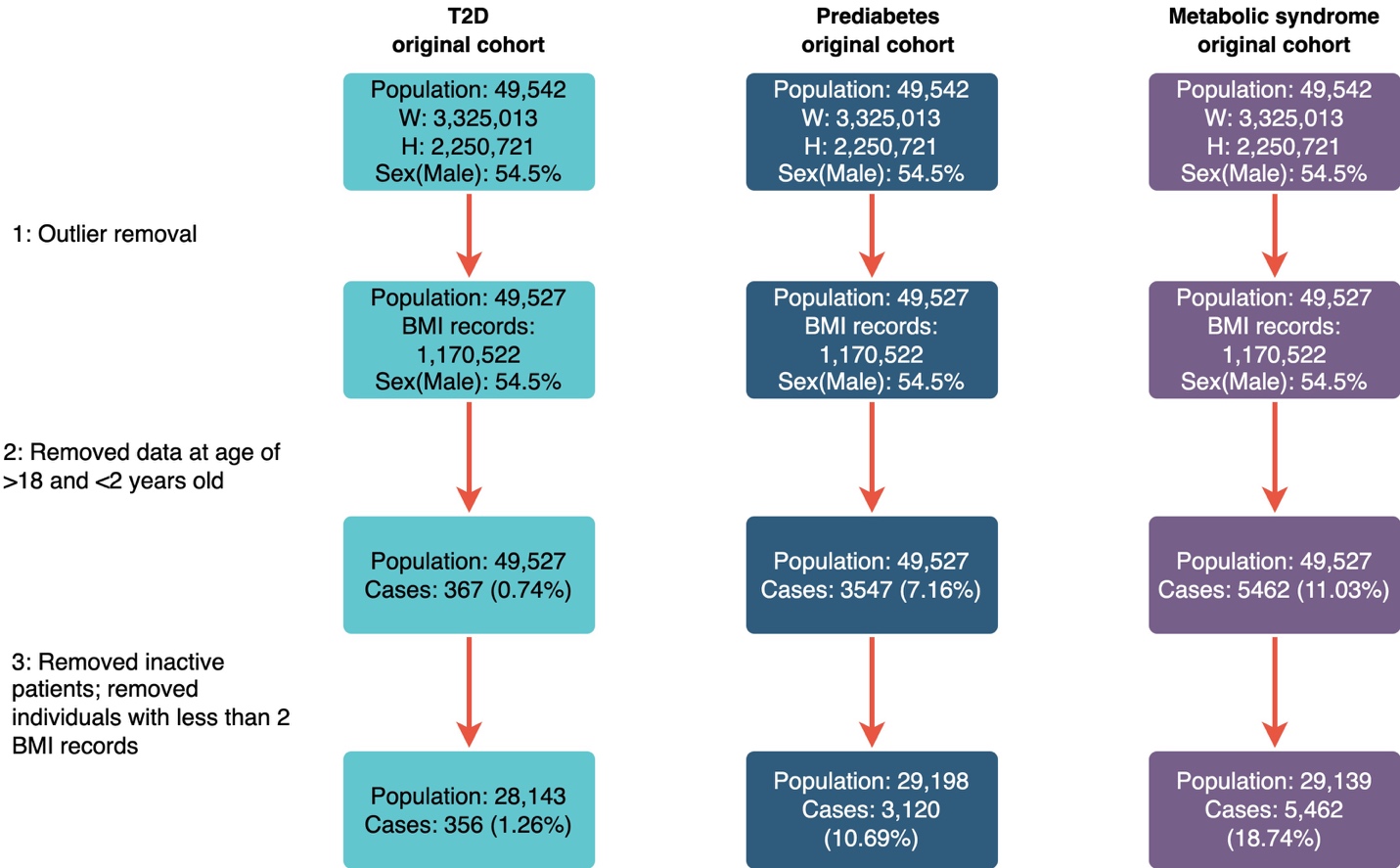


**Supplementary Figure 3.** Detailed STROBE diagram of data processing steps for T2D, prediabetes and metabolic syndrome cohorts.

**Supplementary Table 1.** Type 2 diabetes cohort statistics

| Feature | Controls | Cases | Total |
| --- | --- | --- | --- |
| Participants (n) | 27,787 (98.74 %) | 356 (1.26%) | 28,143 |
| Age range (years) | 2-18 | 2-18 | 2-18 |
| Mean age at T2D diagnosis years (SD) | NA | 13.4 (3.22) | NA |
| Sex | | | |
| Males | 15132 (54.5%) | 117 (32.9%) | 15249 (54.19%) |
| Females | 12655 (45.5%) | 239(67.1%) | 12894 (45.8%) |
| Ethnicity | | | |
| Not Hispanic or Latino | 25372 (91.3%) | 317 (89.0%) | 25689 (91.3%) |
| Hispanic or Latino | 1695 (6.1%) | 32 (9%) | 1727 (6.1%) |
| Unknown | 720 (2.6%) | 7 (2%) | 727 (2.6%) |
| Race | | | |
| Caucasian | 20654, (74.3%) | 187 (52.5%) | 20841 (74.1%) |
| Black | 4248 (15.3%) | 111 (31.2%) | 4359 (15.5%) |
| Asian | 333 (1.2%) | 12 (3.4%) | 345 (1.2%) |
| Multiracial | 1499 (5.4%) | 36 (10.1%) | 1535 (5.5%) |
| Other | 1053 (3.8%) | 10 (2.8%) | 1063 (3.8%) |
| BMI Classification^1^ | | | |
| Underweight | 150 (0.5%) | 1 (0.3%) | 151 (0.5%) |
| Normal weight | 6234 (22.4%) | 23 (6.5%) | 6257 (22.2%) |
| Over-weight | 7155 (25.7%) | 36 (10.1%) | 7191 (25.6%) |
| Obese | 11320 (40.7%) | 139 (39.0%) | 11459 (40.7%) |
| Severe-obese | 2928 (10.5%) | 157 (44.1%) | 3085 (11.0%) |
| Labs | | | |
| HbA1c: Mean (SD) | 6.33 (1.78) | 6.57 (2.15) | 6.58 (2.17) |
| FPG: Mean (SD) | 91.53 (23.05) | 86.24 (17.12) | 86.02 (16.8) |
| Random Glucose: Mean (SD) | 116.24 (61.71) | 109.44 (65.07) | 109.09 (65.22) |
| Health insurance | | | |
| Private health insurance | 14827 (53.4%) | 127 (35.7%) | 14954 (53.1%) |
| Employee health insurance | 2459 (8.8%) | 22 (6.2%) | 2481 (8.8%) |
| Self-pay | 1566 (5.6%) | 15 (4.2%) | 1581 (5.6%) |
| Medicare | 184 (0.7%) | 16 (4.5%) | 200 (0.7%) |
| Medicaid | 6828 (24.6%) | 159 (44.7%) | 6987 (24.8%) |
| Unknown | 1923 (6.9%) | 17 (4.8%) | 1940 (6.9%) |
| Visits | | | |
| Mean patient visits per year | 8.15 | 12.09 | 8.18 |

^1^ BMI was converted to age and sex-specific BMI z-scores and percentiles using the CDC 2000 growth curves [3], then classified into underweight (<5th percentile), normal weight ( 5th, <85th), overweight ( 85th, <95th), obesity ( 95th to <20% higher than the 95th percentile), and severe obesity ( 20% higher than the 95th percentile) [4] .

**Supplementary Table 2.** Prediabetes cohort statistics

| **Feature** | **Controls** | **Cases** | **Total** |
| --- | --- | --- | --- |
| Participants (n) | 26,078 (%89.3) | 3,120 (10.7%) | 29,198 |
| Age range (years) | 2-18 | 2-18 | 2-18 |
| Mean age at T2D diagnosis years (SD) | NA | 12.32 (3.45) | NA |
| Sex | | | |
| Females | 11797 (45.2%) | 1589 (50.9%) | 13386 (45.8%) |
| Ethnicity | | | |
| Not Hispanic or Latino | 23872 (91.5%) | 2746 (88.0%) | 26618 (91.2%) |
| Hispanic or Latino | 1515 (5.8%) | 303 (9.7%) | 1818 (6.2%) |
| Unknown | 691 (2.6%) | 71 (2.3%) | 762 (2.6%) |
| Race | | | |
| Caucasian | 19702 (75.6%) | 1724 (55.3%) | 21426 (73.4%) |
| Black | 3745 (14.4%) | 893 (28.6%) | 4638 (15.9%) |
| Asian | 322 (1.2%) | 41 (1.3%) | 363 (1.2%) |
| Multiracial | 1332 (5.1%) | 313 (10.0%) | 1645 (5.6%) |
| Other | 977 (3.7%) | 149 (4.8%) | 1126 (3.9%) |
| BMI Classification | | | |
| Underweight | 7 (0.0%) | 0 (0.0%) | 7 (0.0%) |
| Normal weight | 699 (2.7%) | 34 (1.1%) | 733 (2.5%) |
| Over-weight | 776 (3.0%) | 52 (1.7%) | 828 (2.8%) |
| Obese | 22167 (85.0%) | 2195 (70.4%) | 24362 (83.4%) |
| Severe-obese | 2429 (9.3%) | 839 (26.9%) | 3268 (11.2%) |
| Labs | | | |
| HbA1c: Mean (SD) | 6.19 (1.84) | 7.30 (2.37) | 6.62 (2.13) |
| FPG: Mean (SD) | 85.03 (12.75) | 90.63 (23.51) | 86.05 (15.45) |
| Random Glucose: Mean (SD) | 98.95 (45.82) | 132.96 (93.70) | 105.65 (59.98) |
| Health insurance | | | |
| Private health insurance | 14071 (54.0%) | 1324 (42.4%) | 15395 (52.7%) |
| Employee health insurance | 2279 (8.7%) | 279 (8.9%) | 2558 (8.8%) |
| Self-pay | 1496 (5.7%) | 115 (3.7%) | 1611 (5.5%) |
| Medicare | 174 (0.7%) | 38 (1.2%) | 212 (0.7%) |
| Medicaid | 6189 (23.7%) | 1195 (38.3%) | 7384 (25.3%) |
| Unknown | 1869 (7.2%) | 169 (5.4%) | 2038 (7.0%) |
| Visits | | | |
| Mean patient visits per year | 7.80 | 11.71 | 8.09 |

**Supplementary Table 3.** Metabolic syndrome cohort statistics

| Feature | Controls | Cases | Total |
| --- | --- | --- | --- |
| Participants (n) | 23,677 (81.3%) | 5,462 (18.7%) | 29,139 |
| Age range (years) | 2-18 | 2-18 | 2-18 |
| Mean age at disease diagnosis years (SD) | NA | 12.54 (2.65) | NA |
| Sex | | | |
| Males | 12979 (54.8%) | 2799, (51.2%) | 15778 (54.1%) |
| Females | 10698 (45.2%) | 2663 (48.8%) | 13361 (45.9%) |
| Ethnicity | | | |
| Not Hispanic or Latino | 21734 (91.8%) | 4820 (88.2%) | 26554 (91.1%) |
| Hispanic or Latino | 1308 (5.5%) | 515 (9.4%) | 1823 (6.3%) |
| Unknown | 635 (2.7%) | 127 (2.3%) | 762 (2.6%) |
| Race | | | |
| Caucasian | 17671 (74.6%) | 3835 (70.2%) | 21506 (73.8%) |
| Black | 3665 (15.5%) | 850 (15.6%) | 4515 (15.5%) |
| Asian | 300 (1.3%) | 64 (1.2%) | 364 (1.2%) |
| Multiracial | 1190 (5.0%) | 437 (8.0%) | 1627 (5.6%) |
| Other | 851 (3.6%) | 276 (5.1%) | 1127 (3.9%) |
| BMI Classification | | | |
| Underweight | 144 (0.6%) | 12 (0.2%) | 156 (0.5%) |
| Normal weight | (24.8%) | 563 (10.3%) | 6434 (22.1%) |
| Over-weight | 6538 (27.6%) | 832 (15.2%) | 7370 (25.3%) |
| Obese | (39.4%) | 2571 (47.1%) | 11892 (40.8%) |
| Severe-obese | 1803 (7.6%) | 1484 (27.2%) | 3287 (11.3%) |
| Labs | | | |
| HbA1c: Mean (SD) | 6.73 (2.17) | 6.10 (1.85) | 6.61 (2.13) |
| FPG: Mean (SD) | 85.97 (16.01) | 85.56 (12.67) | 85.91 (15.59) |
| Random Glucose: Mean (SD) | 104.87 (57.36) | 130.74 (96.67) | 106.88 (61.74) |
| Health insurance | | | |
| Private health insurance | 12854 (54.3%) | 2495 (45.7%) | 15349 (52.7%) |
| Employee health insurance | 2018 (8.5%) | 539 (9.9%) | 2557 (8.8%) |
| Self-pay | 1373 (5.8%) | 237 (4.3%) | 1610 (5.5%) |
| Medicare | 148 (0.6%) | 66 (1.2%) | 214 (0.7%) |
| Medicaid | 5586 (23.6%) | 1785 (32.7%) | 7371 (25.3%) |
| Unknown | 1698 (7.2%) | 340 (6.2%) | 2038 (7.0%) |
| Visits | | | |
| Mean patient visits per year | 7.66 | 9.36 | 7.88 |


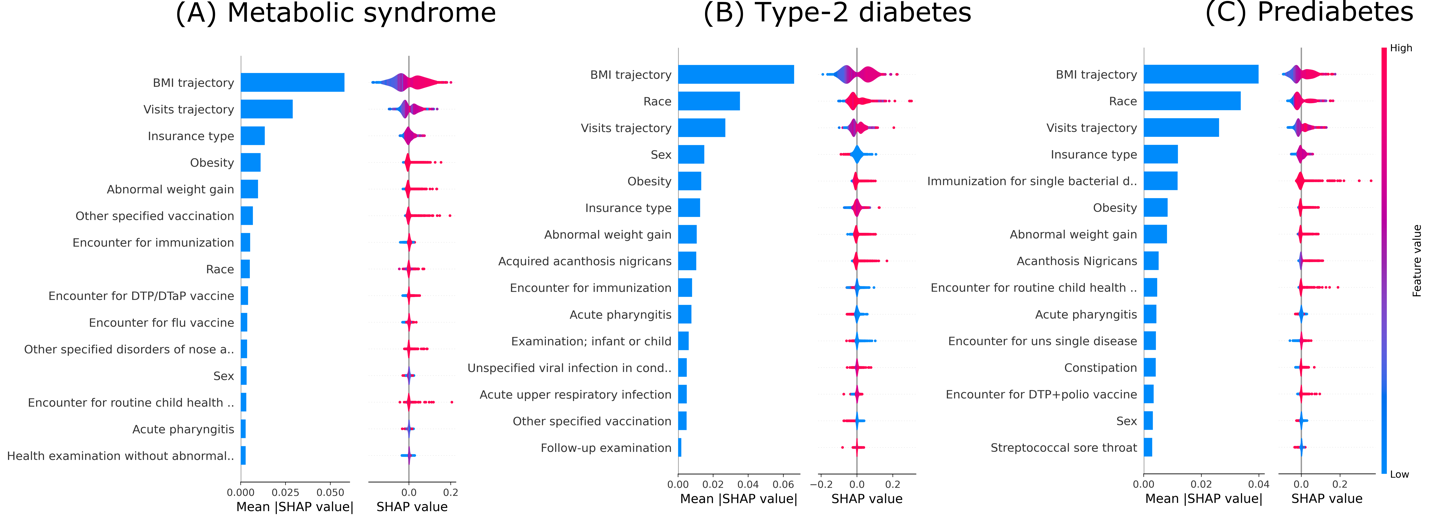


**Supplementary Figure 4.** SHAP values of Random Forest for (A) metabolic syndrome cohorts (B) Type-2 diabetes cohorts (C) prediabetes cohorts. In each subplot, the left plot represents a global feature importance, where the global importance of each feature is taken to be the mean absolute value for that feature over all the given samples. BMI values were normalized using Z-scores, resulting in BMI trajectories that range from a negative to a positive number. As a result, patients with low BMI are represented by light blue color while high BMI values are represented by red dots, and average BMI values are represented by purple color. The right plot, each dot has three characteristics: (i) Vertical location shows what feature it is depicting (ii) Color shows whether that feature was high or low for that row of the dataset, and (iii) Horizontal location shows whether the effect of that value caused a higher or lower prediction. The violin boxplot of BMI trajectory indicated that the majority of individuals with normal BMI trajectory (blue and purple dots) had a lower SHAP value which means they had no or small impact on the model decision making while the abnormal BMI trajectory (red dots) in the most cases increased the likelihood of getting a disease in the prediction window.


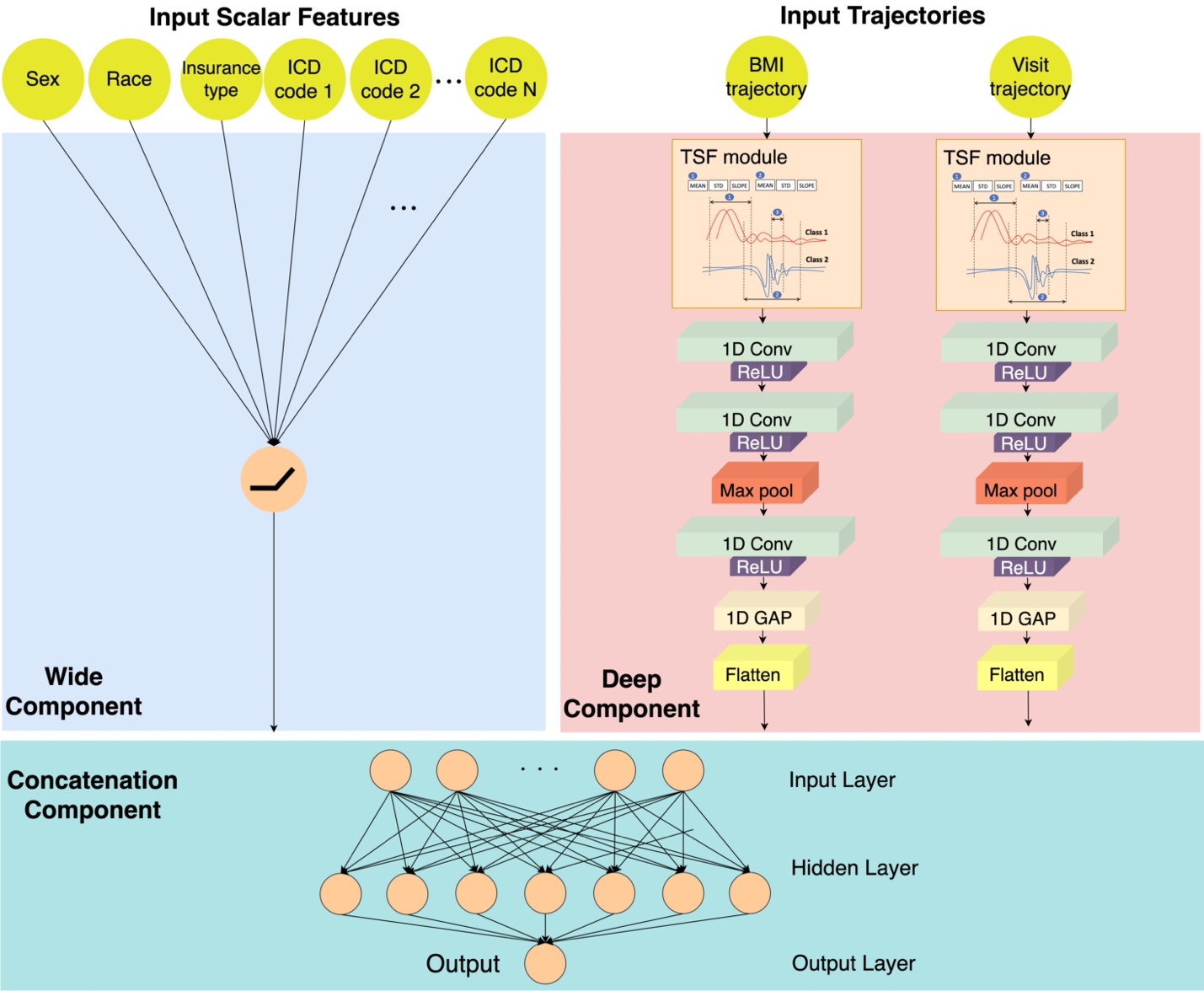


**Supplementary Figure 5.** Architecture of Deep Wide Time series forest with multilayer perceptron Convolutional Neural Network (DW-TSF-CNN)

**Supplementary Table 4.** International Diabetes Federation (IDF) criteria for pediatric metabolic syndrome[5]

| **Age group(years)** | **Obesity** | **Triglycerides** | **HDL-C** | **Blood pressure** | **Glucose** |
| --- | --- | --- | --- | --- | --- |
| 10–<16 | BMI ≥95^th^ percentile^1^ | ≥1.7 mmol/L  (≥150 mg/dL) | <1.03 mmol/L  (<40 mg/dL) | Systolic BP≥130 or  diastolic BP≥85  mm Hg | ≥FPG5.6 mmol/L  (100 mg/dL) or  known T2DM |
| 16+(Adult  criteria) | BMI ≥95^th^ percentile^1^ | ≥1.7 mmol/L  (≥150 mg/dL) or specific treatment for high triglycerides^2^ | <1.03mmol/L  (<40 mg/dL) in males  and <1.29mmol/L  (<50 mg/dL) in females | Systolic BP≥130 or  diastolic BP≥85  mm Hg | ≥FPG5.6 mmol/L  (100 mg/dL) or  known T2DM |

^1^ In this study, BMI≥95^th^ was used instead of waist circumference≥90^th^.  IDF requires the presence of obesity to be defined by increased waist circumference. However, since this is not a common practice in pediatric clinics, we used CDC definition of obesity based on BMI.[3]

^2^ Consuming of Fenofibrate and Gemfibrozil were considered as treatment for triglycerides.

**Supplementary Table 5.** Common selected features used for all prediction models

| Feature name | Data type | Data range |
| --- | --- | --- |
| BMI trajectory | Numerical | Double |
| Visits trajectory | Numerical | Double |
| Sex | Categorical | Male (1), Female (0) |
| Insurance type | Categorical | Private health insurance (0), Employee health insurance (1), Other (2), Military Personnel (3), Self-pay (4), Unknown (5), International Agencies (6), Blue Cross (7), Managed Care (8), Medicare (9), Medicaid (10) |
| Race | Categorical | Caucasian (0), Native Hawaiian or Other Pacific Islander (1), American Indian or Alaska Native (2), Multiracial (3), Other (4), Asian (5), Unknown (6), Black (7) |

**Supplementary Table 6.** Selected ICD codes for metabolic syndrome prediction

| Description | ICD 9 | ICD 10 |
| --- | --- | --- |
| Bacterial infection, unspecified, in conditions classified elsewhere and of unspecified site | 41.9 | B96.89 |
| Hypothyroidism | 244.9 | E03.9 |
| Other adrenocortical overactivity | 255.3 | E27.0 |
| Precocious Puberty | 259.1 | E30.1 |
| Obesity | 278 | E66.9 |
| Other specified disorders of nose and nasal sinuses | 478.19 | J34.89 |
| Other chronic nonalcoholic liver disease | 571.8 | K76.0 |
| Urinary tract infection | 599 | N39.0 |
| Dysmenorrhea | 625.3 | N94.6 |
| Acquired acanthosis nigricans | 701.2 | L83 |
| Other acne | 706.1 | L70.8 |
| Pain in joint involving shoulder region | 719.41 | M25.511 |
| Pain in joint involving forearm | 719.43 | M25.532 |
| Pain in joint involving lower leg | 719.46 | M25.561 |
| Pain in limb | 729.5 | M79.609 |
| Other soft tissue disorders | 729.99 | M70-M79 |
| Syncope | 780.2 | R55 |
| Dizziness and giddiness symptoms | 780.4 | R42 |
| Generalized aches and pains | 780.96 | R52 |
| Abnormal weight gain | 783.1 | R63.5 |
| Abnormal weight loss (finding) | 783.21 | R63.4 |
| Polydipsia | 783.5 | R63.1 |
| Headache | 784 | R51.9 |
| Tachycardia | 785 | R00.0 |
| Chest pain on breathing | 786.52 | R07.1 |
| Other chest pain | 786.59 | R07.89 |
| Other abnormal clinical findings | 796.4 | R68.89 |
| Ankle Sprains | 845 | S93.40 |
| Concussion without loss of consciousness, initial encounter | 850 | S06.0X0A |
| Hand, except finger injury | 959.4 | S69.91XA |
| Finger Injuries | 959.5 |  |
| Allergy, unspecified, NEC in ICD9CM | 995.3 |  |
| Other specified vaccination | V03.89 |  |
| Encounter for immunization | V05.9 | Z23 |
| Encounter due to family history of diabetes mellitus | V18.0 | Z83.3 |
| Health examination without abnormal findings | V20.2 | Z00.129 |
| Other specified conditions influencing health status | V49.89 |  |
| Aftercare for healing traumatic fracture of other bone | V54.19 |  |
| Encounter for other specified aftercare | V58.89 | Z51.89 |
| Exercise counseling | V65.41 | Z71.82 |
| Routine general medical examination at a health care facility | V70.0 | Z00.00 |
| Encounter for other administrative examinations | V70.3 |  |
| Health examination of defined subpopulations | V70.5 |  |
| Pre-operative examination, unspecified | V72.84 | Z01.818 |
| Screening examination for venereal disease in ICD9CM_2008 | V74.5 | Z11.3 |
| Body mass index (BMI) pediatric, 85th percentile to less than 95th percentile for age | V85.53 | Z68.53 |
| Myopia, bilateral | 367.1 | H52.13 |
| Impacted cerumen, bilateral | H61.23 |  |
| Acute suppurative otitis media without spontaneous rupture of ear drum, right ear | H66.001 |  |
| Acute pharyngitis | 462 | J02.9 |
| Acne Vulgaris | L70.0 |  |
| Nasal congestion (finding) | R09.81 |  |
| Lower abdominal pain | R10.30 |  |
| Brain Concussion | S06.0X9 |  |
| Encounter for routine child health examination with abnormal findings | Z00.121 |  |
| Encounter for examination for participation in sport | Z02.5 |  |
| Encounter for screening for depression | Z13.31 |  |
| Other long term (current) drug therapy | Z79.899 |  |
| Other specified postprocedural states | Z98.89 |  |
| Other specified viral infection | 79.89 |  |
| Single major depressive episode | 296.2 | F32.9 |
| Moderate recurrent major depression | 296.32 | F33.1 |
| Generalized Anxiety Disorder | 300.02 |  |
| Unspecified nonpsychotic mental disorder | 300.9 | F48.9 |
| Visual disturbance | 368.9 |  |
| Acute suppurative otitis media without spontaneous rupture of ear drum | 382 |  |
| Genital Diseases, Male | 608.9 |  |
| Other specified noninflammatory disorders of vagina | 623.8 |  |
| Onychia and paronychia of toe | 681.11 |  |
| Contact dermatitis and other eczema due to plants [except food] | 692.6 | L25 |
| Hirsutism | 704.1 | L68.0 |
| Scar conditions and fibrosis of skin | 709.2 | L90.5 |
| Low Back Pain | 724.2 | M54.50 |
| Idiopathic scoliosis and kyphoscoliosis | 737.3 | M41.20 |
| Other malaise and fatigue | 780.79 | R53.81 |
| Palpitations | 785.1 | R00.2 |
| Polyuria | 788.42 | R35.89 |
| Epigastric pain | 789.06 | R10.13 |
| Sprains and strains of unspecified site of shoulder and upper arm | 840.9 |  |
| Sprains and strains of unspecified site of knee and leg | 844.9 |  |
| Encounter due to contact with or exposure to other viral disease | V01.79 |  |
| Need for prophylactic vaccination and inoculation against influenza | V04.81 |  |
| Need for prophylactic vaccination and inoculation against other viral diseases | V04.89 |  |
| Need for prophylactic vaccination and inoculation against diphtheria-tetanus-pertussis, combined [DTP] [DTaP] | V06.1 |  |
| Other development of adolescence | V21.2 | Z00.3 |
| General counseling on prescription of oral contraceptives | V25.01 | Z30.011 |
| Encounter for surveillance of contraceptive pills | V25.41 | Z30.41 |
| Encounter due to problems with sight | V41.0 | Z97.3 |
| Feeling suicidal (finding) | V62.84 | R45.851 |
| Routine gynecological examination | V72.31 | Z01.419 |
| Encounter due to screening for depression | V79.0 |  |
| Melanocytic nevus | D22 |  |
| Morbid obesity | 278.01 | E66.01 |
| Other chronic allergic conjunctivitis | 372.14 | H10.45 |
| Nails, Ingrown | 703 | L60.0 |
| Pain in right hand only | M79.641 |  |
| Snoring | 786.09 | R06.83 |
| Chest Pain | 786.5 | R07.9 |
| Sprain of unspecified ligament of right ankle, initial encounter | S93.401A |  |
| Other injuries of unspecified body region | T14.8 |  |
| Encounter for examination of eyes and vision with abnormal findings | V72.0 | Z01.01 |
| Encounter for screening for COVID-19 | Z11.52 |  |
| Encounter for screening for lipoid disorders | V77.91 | Z13.220 |
| Contact with and (suspected) exposure to COVID-19 | Z20.822 |  |
| Encounter for other general counseling and advice on contraception | V25.09 | Z30.09 |

**Supplementary Table 7.** Selected ICD codes for T2D prediction

| Description | ICD 9 | ICD 10 |
| --- | --- | --- |
| Obesity | 278 | E66.9 |
| Acute pharyngitis | 462 | J02.9 |
| Acquired acanthosis nigricans | 701.2 | L83 |
| Other Acne | 706.1 | L70.8 |
| Hypersomnia with sleep apnea, unspecified | 780.53 |  |
| Abnormal weight gain | 783.1 | R63.5 |
| Nonspecific abnormal results of other endocrine function study | 794.6 | R94.7 |
| Encounter for immunization | V05.9 | Z23 |
| Other specified vaccination | V03.89 |  |
| Examination; infant or child | V20.2 | Z00.129 |
| Unspecified viral infection in conditions classified elsewhere and of unspecified site | 79.99 | B97.89 |
| Polycystic Ovary Syndrome | 256.4 | E28.2 |
| Disorder of lipoprotein metabolism, unspecified | 272.9 | E78.9 |
| Abscess of vulva | 616.4 | N76.4 |
| Nasal congestion (finding) | R09.81 |  |
| Encounter for routine child health examination with abnormal findings | Z00.121 |  |
| Encounter for other specified aftercare | V58.89 | Z51.89 |
| Encounter due to family history of diabetes mellitus | V18.0 | Z83.3 |
| Essential Hypertension | 401.9 | I10 |
| Acute upper respiratory infection | 465.9 | J00-J06 |
| Body mass index (BMI) pediatric, 85th percentile to less than 95th percentile for age | V85.53 | Z68.53 |
| Acne vulgaris | L70.0 |  |
| Generalized aches and pains | 780.96 | R52 |
| Other general symptoms | 780.99 | R68.89 |
| Routine general medical examination at a health care facility | V70.0 | Z00.00 |
| Contact with and (suspected) exposure to other viral communicable diseases | Z20.828 |  |
| Nausea and vomiting | 787.01 | R11.2 |
| Other hyperalimentation | 278.8 | E67.8 |
| Borderline Personality Disorder | 301.83 | F60.3 |
| Postprocedural acute respiratory failure | 518.51 | J95.821 |
| Syndrome of infant of diabetic mother | 775 | P70.1 |
| Follow-up examination | V67.9 | Z09 |
| Coagulation defects, other and unspecified | 286.7 | D68 |
| Disintegrative psychosis, current or active state | 299.1 |  |
| Knee, leg, ankle, and foot injury | 959.7 |  |
| Other chronic pain | G89.29 |  |
| Newborn affected by maternal factors and by complications of pregnancy, labor, and delivery | P00-P04 |  |
| Nausea | R11.0 |  |
| Encounter for examination for adolescent development state | Z00.3 |  |
| Contact with and (suspected) exposure to COVID-19 | Z20.822 |  |
| Dependence on respirator [ventilator] status | Z99.11 |  |

**Supplementary Table 8.** Selected ICD codes for prediabetes prediction

| Description | ICD 9 | ICD 10 |
| --- | --- | --- |
| Abnormal auditory function study | R94.120 | 794.15 |
| Hand, Foot and Mouth Disease | 74.3 | B08.4 |
| Viral exanthem | 57.9 | B09 |
| Infectious Mononucleosis | 75 | B27 |
| Other specified viral infection | 79.89 | B33.8 |
| Candidiasis of skin and nails | 112.3 | B37.2 |
| Bacterial infection, unspecified, in conditions classified elsewhere and of unspecified site | 41.9 | B96.89 |
| Immunologic Deficiency Syndromes | 279.3 | D84.9 |
| Unspecified disorder of immune mechanism | 279.9 | D89.9 |
| Activity, american tackle football | Y93.61 | E007.0 |
| Other adrenocortical overactivity | 255.3 | E27.0 |
| Precocious Puberty | 259.1 | E30.1 |
| Localized adiposity | 278.1 | E65 |
| Obesity | 278 | E66.9 |
| Dehydration | 276.51 | E86.0 |
| Hypovolemia | 276.5 | E86.9 |
| Hypokalemia | 276.8 | E87.6 |
| Cannabis abuse, unspecified use | 305.2 | F12.10 |
| Mild major depression, single episode | 296.21 | F32.0 |
| Single major depressive episode | 296.2 | F32.9 |
| Moderate recurrent major depression | 296.32 | F33.1 |
| Phobia, Social | 300.23 | F40.10 |
| Generalized Anxiety Disorder | 300.02 | F41.1 |
| Other eating disorders | 307.59 | F50.8 |
| Intermittent Explosive Disorder | 312.34 | F63.81 |
| Developmental expressive language disorder | 315.31 | F80.9 |
| Attention-Deficit/Hyperactivity Disorder, Predominantly Inattentive Type | 314 | F90.0 |
| Conduct Disorder | 312.9 | F91.9 |
| Migraine without aura, not intractable, without status migrainosus | 346.1 | G43.009 |
| Migraine, unspecified, without mention of intractable migraine without mention of status migrainosus | 346.9 | G43.909 |
| Tension Headache | 339.1 | G44.20 |
| Anisometropia | 367.31 | H52.31 |
| Acute suppurative otitis media without spontaneous rupture of ear drum | 382 | H66.009 |
| Hypotension, Orthostatic | 458 | I95.1 |
| Acute upper respiratory infection | 465.9 | J00-J06 |
| Other acute sinusitis | 461.8 | J01.80 |
| Streptococcal sore throat | 34 | J02.0 |
| Acute pharyngitis | 462 | J02.9 |
| Acute bronchiolitis due to respiratory syncytial virus | 466.11 | J21.0 |
| Chronic sinusitis | 473.9 | J32.9 |
| Hypertrophy of tonsils alone | 474.11 | J35.1 |
| Teething syndrome | 520.7 | K00.7 |
| Constipation | 564 | K59.00 |
| Diaper Rash | 691 | L22 |
| Nails, Ingrown | 703 | L60.0 |
| Other acne | 706.1 | L70.8 |
| Acanthosis Nigricans | 701.2 | L83 |
| Scar conditions and fibrosis of skin | 709.2 | L90.5 |
| Pain in joint involving lower leg | 719.46 | M25.569 |
| Low Back Pain | 724.2 | M54.5 |
| Back Pain | 724.5 | M54.9 |
| Pain in limb | 729.5 | M79.609 |
| Hypertrophy of Breast | 611.1 | N62 |
| Irregular periods | 626.4 | N92.6 |
| Dysmenorrhea | 625.3 | N94.6 |
| Feeding problems in newborn | 779.31 | P92 |
| Coughing | 786.2 | R05 |
| Dyspnea | 786.05 | R06.00 |
| Apnea | 786.03 | R06.81 |
| Tachypnea | 786.06 | R06.82 |
| Other chest pain | 786.59 | R07.89 |
| Chest Pain | 786.5 | R07.9 |
| Vomiting alone | 787.03 | R11.10 |
| Urinary Incontinence | 788.3 | R32 |
| Polyuria | 788.42 | R35.89 |
| Irritable Mood | 799.22 | R45.4 |
| fever presenting with condition classified elsewhere | 780.61 | R50.81 |
| Generalized aches and pains | 780.96 | R52 |
| Other malaise and fatigue | 780.79 | R53.81 |
| Syncope | 780.2 | R55 |
| Delayed developmental milestones | 783.42 | R62.0 |
| Polydipsia | 783.5 | R63.1 |
| Feeding difficulties and mismanagement | 783.3 | R63.3 |
| Abnormal weight gain | 783.1 | R63.5 |
| Other nonspecific abnormal finding of lung field | 793.19 | R91.8 |
| Concussion with no loss of consciousness | 850 | S06.0X0A |
| Brain Concussion | 850.9 | S06.0X9 |
| Ankle Sprains | 845 | S93.40 |
| Health supervision for newborn 8 to 28 days old | V20.32 | Z00.111 |
| Encounter for routine child health examination with abnormal findings | Z00.121 |  |
| Encounter for screening for respiratory tuberculosis | V74.1 | Z11.1 |
| Encounter for screening for lipoid disorders | V77.91 | Z13.220 |
| Encounter due to screening for depression | V79.0 | Z13.31 |
| Encounter for surveillance of contraceptive pills | V25.41 | Z30.41 |
| Encounter due to contraceptive management | V25.9 | Z30.9 |
| Single liveborn infant, delivered vaginally | V30.00 | Z38.00 |
| single liveborn born in hospital and delivered by cesarean section | V30.01 | Z38.01 |
| Weeks pregnant | 765.2 | Z3A |
| Encounter for other specified aftercare | V58.89 | Z51.89 |
| Body mass index (BMI) pediatric, 5th percentile to less than 85th percentile for age | V85.52 | Z68.52 |
| Body mass index (BMI) pediatric, 85th percentile to less than 95th percentile for age | V85.53 | Z68.53 |
| Exercise counseling | V65.41 | Z71.82 |
| Encounter for other specified counseling | V65.49 | Z71.89 |
| Encounter due to high-risk sexual behavior | V69.2 | Z72.5 |
| Encounter due to family history of diabetes mellitus | V18.0 | Z83.3 |
| Personal history of other infectious and parasitic disease | V12.09 | Z86.19 |
| Personal history of diseases of the nervous system and sense organs | V12.40 | Z86.69 |
| Other specified viral warts | 78.19 |  |
| Benign neoplasm of skin of trunk, excluding scrotum | 216.5 |  |
| Benign neoplasm of skin | 216.9 |  |
| Other hyperlipidemia | 272.4 | E78.5 |
| Hyposmolality and/or hyponatremia | 276.1 | E87.1 |
| Anxiety state | 300 |  |
| Tobacco Use Disorder | 305.1 |  |
| Adjustment disorder with mixed disturbance of emotions AND conduct | 309.4 |  |
| Other developmental speech or language disorder | 315.39 |  |
| Other acute pain | 338.19 |  |
| Hyperopia | 367 |  |
| Conjunctivitis | 372.3 |  |
| Infective otitis externa | 380.1 |  |
| Other acute otitis externa | 380.22 |  |
| Acute bronchiolitis due to other infectious organisms | 466.19 |  |
| Deviated nasal septum | 470 |  |
| Enlargement of tonsil or adenoid | 474.1 |  |
| Other chronic nonalcoholic liver disease | 571.8 |  |
| Mass in breast | 611.72 |  |
| Unspecified symptom associated with female genital organs | 625.9 |  |
| Onychia and paronychia of toe | 681.11 |  |
| Contact dermatitis and other eczema due to plants [except food] | 692.6 |  |
| Other specified hypertrophic and atrophic conditions of skin | 701.8 |  |
| Epithelial cyst | 706.2 |  |
| Joint effusion of ankle AND/OR foot | 719.07 |  |
| Pain in joint involving shoulder region | 719.41 |  |
| Pain in joint involving forearm | 719.43 |  |
| Idiopathic scoliosis and kyphoscoliosis | 737.3 |  |
| Dizziness and giddiness symptoms | 780.4 |  |
| Sleep Apnea Syndromes | 780.57 |  |
| SLEEP DISTURBANCES NEC in ICD9CM | 780.59 |  |
| Fever and other physiologic disturbances of temperature regulation | 780.6 |  |
| Gait abnormality | 781.2 |  |
| Skin sensation disturbance | 782 |  |
| Jaundice (not of newborn) | 782.4 |  |
| Unspecified lack of expected normal physiological development in childhood | 783.4 |  |
| Hyperphagia | 783.6 |  |
| Headache | 784 |  |
| Other respiratory abnormalities | 786.09 | R06.83 |
| Other abnormal blood chemistry | 790.6 | R79.89 |
| Closed fracture of clavicle | 810 |  |
| Closed fracture carpal bone | 814 |  |
| Closed fracture of metacarpal bone | 815 |  |
| Closed fracture of one or more phalanges of hand | 816 |  |
| Closed traumatic dislocation of patellofemoral joint | 836.3 |  |
| Sprains and strains of unspecified site of shoulder and upper arm | 840.9 |  |
| Sprain of wrist | 842 |  |
| Sprains and strains of other specified sites of hip and thigh | 843.8 |  |
| Sprain of cruciate ligament of knee | 844.2 |  |
| Other sprains and strains of ankle | 845.09 |  |
| Lumbar sprain | 847.2 |  |
| Open wound of hand except fingers alone, without mention of complication | 882 |  |
| Open wound of finger without complication | 883 |  |
| Shoulder and upper arm injury | 959.2 |  |
| Elbow, forearm, and wrist injury | 959.3 |  |
| Hand, except finger injury | 959.4 |  |
| Knee, leg, ankle, and foot injury | 959.7 |  |
| Unspecified site injury | 959.9 |  |
| Drug toxicity | 977.9 |  |
| Other viral warts | B07.8 |  |
| Viral warts, unspecified | B07.9 |  |
| Coronavirus Infections | B34.2 |  |
| Melanocytic nevus | D22 |  |
| Neoplasm of uncertain or unknown behavior of skin | D48.5 |  |
| Iron deficiency | E61.1 |  |
| Motor vehicle traffic accident of unspecified nature injuring unspecified person | E819.9 |  |
| Cannabis use, unspecified, uncomplicated | F12.90 |  |
| Autistic Disorder | F84.0 |  |
| Other specified behavioral and emotional disorders with onset usually occurring in childhood and adolescence | F98.8 |  |
| Hypermetropia, bilateral | H52.03 |  |
| Myopia, bilateral | H52.13 |  |
| Otitis Externa | H60.90 |  |
| Otitis media, unspecified, bilateral | H66.93 |  |
| Acute bronchiolitis | J21.9 |  |
| Other allergic rhinitis | J30.89 |  |
| Bronchospasm, Exercise-Induced | J45.990 |  |
| Cellulitis of right toe | L03.031 |  |
| Cellulitis of left toe | L03.032 |  |
| Allergic contact dermatitis due to plants, except food | L23.7 |  |
| Acne Vulgaris | L70.0 |  |
| right shoulder joint pain | M25.511 |  |
| Shoulder Pain | M25.519 |  |
| Pain in a joint, ankle and foot | 719.47 | M25.572 |
| Other idiopathic scoliosis, site unspecified | M41.20 |  |
| Scoliosis, unspecified | M41.9 |  |
| Low back pain, unspecified | M54.50 |  |
| Other enthesopathies, not elsewhere classified in ICD10CM | M77.8 |  |
| Pain in right hand only | M79.641 |  |
| Pain in left toe(s) | M79.675 |  |
| Other specified disorders of bone density and structure | M85.80 |  |
| Newborn affected by maternal factors and by complications of pregnancy, labor, and delivery (P00-P04) | P00-P04 |  |
| Neonatal Jaundice | P59.9 |  |
| Acute onset cough | R05.1 |  |
| Other abnormalities of breathing | R06.89 |  |
| Icterus | R17 |  |
| Anesthesia of skin | R20.0 |  |
| Other symptoms and signs involving appearance and behavior | R46.89 |  |
| Other speech disturbances | R47.89 |  |
| Other fatigue | R53.83 |  |
| Other feeding difficulties | R63.39 |  |
| Fussy infant (baby) | R68.12 |  |
| Unspecified abnormal findings in urine | R82.90 |  |
| Concussion with loss of consciousness of unspecified duration, initial encounter | S06.0X9A |  |
| Strain of muscle, fascia and tendon of lower back, initial encounter | S39.012A |  |
| Unspecified fracture of unspecified metacarpal bone, initial encounter for closed fracture | S62.309A |  |
| Fracture of unspecified phalanx of unspecified finger, initial encounter for closed fracture | S62.609A |  |
| Unspecified sprain of unspecified wrist, initial encounter | S63.509A |  |
| Unspecified injury of unspecified wrist, hand and finger(s), initial encounter | S69.90XA |  |
| Other injuries of unspecified body region | T14.8 |  |
| Injury, unspecified, initial encounter | T14.90XA |  |
| Encounter due to contact with or exposure to other viral disease | V01.79 |  |
| Other specified vaccinations against hemophilus influenza, type B [Hib] | V03.81 |  |
| Other specified vaccinations against streptococcus pneumoniae [pneumococcus] | V03.82 |  |
| Need for immunization against other single bacterial diseases | V03.89 |  |
| Need for prophylactic vaccination and inoculation against unspecified single bacterial disease | V03.9 |  |
| Need for prophylactic vaccination and inoculation against other viral diseases | V04.89 |  |
| Need for prophylactic vaccination and inoculation against unspecified single disease | V05.9 | Z23 |
| Need for prophylactic vaccination and inoculation against diphtheria-tetanus-pertussis, combined [DTP] [DTaP] | V06.1 |  |
| Need for prophylactic vaccination and inoculation against diptheria-tetanus- pertussis with poliomyelitis [DTP + polio] | V06.3 |  |
| Need for immunization against measles-mumps-rubella [MMR] | V06.4 |  |
| Need for prophylactic vaccination and inoculation against tetanus-diphtheria [Td] (DT) | V06.5 |  |
| examination; infant or child | V20.2 | Z00.129 |
| Encounter for newborn health supervision | V20.3 |  |
| Health supervision for newborn under 8 days old | V20.31 | Z00.110 |
| Other development of adolescence | V21.2 |  |
| General counseling on prescription of oral contraceptives | V25.01 | Z30.011 |
| Observation for suspected infectious condition | V29.0 |  |
| Encounter due to mental and behavioral problems | V40.9 |  |
| Aftercare for healing traumatic fracture of other bone | V54.19 |  |
| Vaccination not carried out because of patient refusal | V64.06 |  |
| Follow-up examination, following surgery, unspecified | V67.00 |  |
| Routine general medical examination at a health care facility | V70.0 | Z00.00 |
| Other general medical examination for administrative purposes | V70.3 | Z02.89 |
| Health examination of defined subpopulations | V70.5 |  |
| Encounter due to Unspecified general medical examination | V70.9 |  |
| Encounter for examination of eyes and vision in ICD9CM_2008 | V72.0 |  |
| Routine gynecological examination | V72.31 | Z01.419 |
| Special screening examination for other specified viral diseases | V73.89 |  |
| Screening examination for venereal disease in ICD9CM_2008 | V74.5 | Z11.3 |
| Screening for developmental handicaps in early childhood | V79.3 |  |
| Screening for unspecified mental disorder and developmental handicap | V79.9 |  |
| Screening for chemical poisoning and other contamination | V82.5 |  |
| Person injured in unspecified motor-vehicle accident, traffic, initial encounter | V89.2XXA |  |
| Encounter for examination for adolescent development state | Z00.3 |  |
| Encounter for examination for participation in sport | Z02.5 |  |
| Encounter for follow-up examination after completed treatment for conditions other than malignant neoplasm | Z09 |  |
| Encounter for screening for COVID-19 | Z11.52 |  |
| Encounter for screening for global developmental delays (milestones) | Z13.42 |  |
| Encounter for screening for disorder due to exposure to contaminants | Z13.88 |  |
| Contact with and (suspected) exposure to infections with a predominantly sexual mode of transmission | Z20.2 |  |
| Contact with and (suspected) exposure to COVID-19 | Z20.822 |  |
| Procedure and treatment not carried out due to patient leaving prior to being seen by health care provider | V64.2 | Z53.21 |
| Encounter for immunization safety counseling | Z71.85 |  |
| Other long term (current) drug therapy | Z79.899 |  |
| Encounter due to acquired absence of other organs | Z90.89 |  |
| Encounter due to allergy to milk products | Z91.011 |  |
| Other specified postprocedural states | Z98.89 |  |

# Hyperparameter Tuning

Hyperparameter tuning was conducted for the DW-TSF-CNN model, considering dropout, batch size, convolution kernel size, convolution kernel width, and dense size. In the case of the Random Forest model, tuning was performed to optimize parameters such as the number of trees, number of features considered at each split, maximum depth, minimum number of samples required to split a node, minimum number of samples required at each leaf node, and the method of selecting samples for training each tree. Convolution kernel size, convolution kernel width, LSTM width, and batch size was considered for CRNN model. For the Transformer model, number of heads, head size, FF size, and drop out for its multi-head attention block, and dense size, dropout, batch size, and number of encoder blocks were explored.

Hyperparameter tuning was conducted for the DW-TSF-CNN model, taking into account dropout, batch size, convolution kernel size, convolution kernel width, and dense size. Similarly, for the Random Forest model, parameters such as the number of trees, number of features at each split, maximum depth, minimum number of samples to split a node, minimum number of samples at each leaf node, and the method of sample selection were optimized.

In the CRNN model, tuning was performed on convolution kernel size, convolution kernel width, LSTM width, and batch size. As for the Transformer model, the number of heads, head size, FF size, and dropout within the multi-head attention block were explored. Additionally, dense size, dropout, batch size, and the number of encoder blocks were considered.

Each model underwent thorough exploration and tuning of the respective hyperparameters to optimize their performance. The bandit-based approach [6] was employed for DW-TSF-CNN, CRNN, and Transformer models, while random search [7] was used for the Random Forest model. The hyperparameter space was explored for up to 300 iterations, as outlined in (Supplementary Table 9).

**Supplementary Table 9.** Hyperparameter tuning variables and ranges

| Category | Name | Type | Range | Optimizer method |
| --- | --- | --- | --- | --- |
| DW-TSF-CNN | Convolution kernel size | Int | [8, 32, 64, 128, 512] | Hyperband |
|  | Convolution kernel width | Int | [1, 3] | Hyperband |
|  | Dense size | Int | [1, 10, 100] | Hyperband |
|  | Drop out | Float | [0.1 - 0.8] | Hyperband |
|  | Batch size | Int | [32, 64, 128, 256] | Hyperband |
| Random Forest | # of trees | Int | [200 - 2000], step =10 | Random grid search |
|  | # of features at every split | Categorical | [auto, sqrt] | Random grid search |
|  | Min # of samples required to split a node | Int | [2, 5, 10] | Random grid search |
|  | Min # of samples required at each leaf node | Int | [1, 2, 4] | Random grid search |
|  | Bootstrap | Bool | [True, False ] | Random grid search |
|  | Max depth | Int | [10, 110], step = 11 | Random grid search |
| Transformer | Multihead attention: # of Head | Int | [1, 2, 4, 8] | Hyperband |
|  | Multihead attention: Head size | Int | [1, 2, 4, 8] | Hyperband |
|  | Multihead attention: feed forward | Int | [1, 2, 4, 8] | Hyperband |
|  | Multihead attention: drop out | Float | [0-0.8], step 0.1 | Hyperband |
|  | Dense size | Int | [32,64,128,256] | Hyperband |
|  | Drop out | Float | [0-0.8], step 0.1 | Hyperband |
|  | # Encoder blocks | Int | [1,2,3,4] | Hyperband |
|  | Batch size | Int | [32, 64, 128, 256] | Hyperband |
| CRNN | Convolution kernel size | Int | [32, 64, 128, 256, 512] | Hyperband |
|  | Convolution kernel width | Int | [3,5,8] | Hyperband |
|  | LSTM Width |  | [4,8,16,32] |  |
|  | Batch size | Int | [32, 64, 128, 256] | Hyperband |

# System specification

All data processing was performed using R 4.1.1,[8] including packages *pROC,*[9] ggpubr, and *caret*[10]. Python 3.7[11] was used for the implementation of models which is based on TensorFlow backend and Keras API. Python packages that were used for model preparation and testing were *shap*,[12] *sklearn*,[13] and *keras_tuner.*[14] Models were generated using the Cleveland Clinic’s High Performance Computing Cluster. We issued multiple jobs, each including the process of training, tuning, validation, testing of a deep learning model. For each job, we allocated 48 cores (96 threads) run using Intel® Xeon® Platinum 8468 Processor (105M Cache, 2.10 GHz), with dedicated memory allocation of 3 TB RAM with CentOS Linux v8 operating system.

**Supplementary Table 10.** Type-2 Diabetes cohorts statistics

| Ages (years) | Cohort size  (N) | Cases | Sex %  Female | | | Race %  White | Median Test AUC(SD) | | | | | | | Mean Cross Validation AUC | | | |
| --- | --- | --- | --- | --- | --- | --- | --- | --- | --- | --- | --- | --- | --- | --- | --- | --- | --- |
|  |  |  |  |  |  |  | DW-TSF-CNN | | Random Forest | | CRNN | Transformer | | DW-TSF-CNN | CRNN | Transformer | Random Forest |
| 2 to 4 | 3691 | 4(0.1%) | | 42.80% | 83.90% | | | -^*^ | | - | - | | - |  |  |  |  |
| 2 to 5 | 3691 | 4(0.1%) | | 42.80% | 83.90% | | | - | | - | - | | - |  |  |  |  |
| 2 to 6 | 3691 | 4(0.1%) | | 42.80% | 83.90% | | | - | | - | - | | - |  |  |  |  |
| 2 to 7 | 3687 | 2(0.1%) | | 42.70% | 83.90% | | | - | | - | - | | - |  |  |  |  |
| 2 to 8 | 3681 | 2(0.1%) | | 42.70% | 83.90% | | | - | | - | - | | - |  |  |  |  |
| 2 to 9 | 3673 | 1(0.0%) | | 42.70% | 83.90% | | | - | | - | - | | - |  |  |  |  |
| 2 to 10 | 3659 | 1(0.0%) | | 42.70% | 83.90% | | | - | | - | - | | - |  |  |  |  |
| 2 to 11 | 3650 | 0(0.0%) | | 42.70% | 83.90% | | | - | | - | - | | - |  |  |  |  |
| 2 to 12 | 3638 | 0(0.0%) | | 42.80% | 83.80% | | | - | | - | - | | - |  |  |  |  |
| 2 to 13 | 3617 | 0(0.0%) | | 42.70% | 83.80% | | | - | | - | - | | - |  |  |  |  |
| 2 to 14 | 3561 | 0(0.0%) | | 42.90% | 83.80% | | | - | | - | - | | - |  |  |  |  |
| 3 to 5 | 7774 | 32(0.4%) | | 43.30% | 79.60% | | | 0.75(0.01) | | 0.52(0.0) | 0.65(0.04) | | 0.72(0.07) | 0.9 | 0.83 | 0.83 | 0.7 |
| 3 to 6 | 7774 | 32(0.4%) | | 43.30% | 79.60% | | | 0.68(0.03) | | 0.44(0.01) | 0.7(0.02) | | 0.72(0.08) | 0.86 | 0.83 | 0.75 | 0.6 |
| 3 to 7 | 7769 | 30(0.4%) | | 43.40% | 79.60% | | | 0.81(0.01) | | 0.64(0.03) | 0.69(0.15) | | 0.7(0.11) | 0.88 | 0.72 | 0.7 | 0.71 |
| 3 to 8 | 7755 | 29(0.4%) | | 43.40% | 79.60% | | | 0.88(0.01) | | 0.72(0.04) | 0.7(0.2) | | 0.81(0.07) | 0.66 | 0.67 | 0.68 | 0.65 |
| 3 to 9 | 7738 | 27(0.3%) | | 43.30% | 79.60% | | | 0.95(0.01) | | 0.76(0.01) | 0.82(0.11) | | 0.85(0.07) | 0.88 | 0.65 | 0.58 | 0.7 |
| 3 to 10 | 7716 | 27(0.3%) | | 43.40% | 79.60% | | | 0.59(0.14) | | 0.75(0.0) | 0.71(0.06) | | 0.82(0.05) | 0.45 | 0.74 | 0.64 | 0.7 |
| 3 to 11 | 7694 | 25(0.3%) | | 43.40% | 79.60% | | | 0.89(0.01) | | 0.75(0.0) | 0.79(0.07) | | 0.85(0.03) | 0.96 | 0.63 | 0.86 | 0.88 |
| 3 to 12 | 7659 | 18(0.2%) | | 43.50% | 79.60% | | | 0.69(0.03) | | 0.62(0.04) | 0.59(0.09) | | 0.61(0.1) | 0.72 | 0.76 | 0.61 | 0.39 |
| 3 to 13 | 7589 | 17(0.2%) | | 43.50% | 79.60% | | | 0.84(0.21) | | 0.79(0.0) | 0.61(0.24) | | 0.85(0.1) | 0.75 | 0.51 | 0.69 | 0.54 |
| 3 to 14 | 7451 | 14(0.2%) | | 43.50% | 79.60% | | | 0.95(0.01) | | 0.78(0.0) | 0.61(0.34) | | 0.88(0.05) | 0.78 | 0.78 | 0.72 | 0.61 |
| 4 to 6 | 10573 | 58(0.5%) | | 43.80% | 78.40% | | | 0.78(0.01) | | 0.61(0.02) | 0.58(0.03) | | 0.78(0.03) | 0.82 | 0.82 | 0.78 | 0.73 |
| 4 to 7 | 10568 | 56(0.5%) | | 43.80% | 78.40% | | | 0.73(0.01) | | 0.63(0.0) | 0.61(0.06) | | 0.67(0.05) | 0.81 | 0.76 | 0.75 | 0.71 |
| 4 to 8 | 10550 | 54(0.5%) | | 43.90% | 78.40% | | | 0.77(0.01) | | 0.69(0.0) | 0.65(0.05) | | 0.74(0.03) | 0.87 | 0.79 | 0.8 | 0.73 |
| 4 to 9 | 10529 | 52(0.5%) | | 43.80% | 78.40% | | | 0.83(0.01) | | 0.63(0.0) | 0.7(0.02) | | 0.66(0.13) | 0.75 | 0.74 | 0.71 | 0.71 |
| 4 to 10 | 10499 | 51(0.5%) | | 43.90% | 78.30% | | | 0.75(0.02) | | 0.7(0.0) | 0.67(0.08) | | 0.76(0.03) | 0.88 | 0.72 | 0.79 | 0.67 |
| 4 to 11 | 10471 | 46(0.4%) | | 43.90% | 78.30% | | | 0.79(0.15) | | 0.79(0.0) | 0.57(0.09) | | 0.8(0.03) | 0.75 | 0.73 | 0.74 | 0.65 |
| 4 to 12 | 10429 | 38(0.4%) | | 43.90% | 78.30% | | | 0.53(0.18) | | 0.77(0.0) | 0.69(0.05) | | 0.67(0.08) | 0.78 | 0.82 | 0.8 | 0.73 |
| 4 to 13 | 10337 | 36(0.3%) | | 44.00% | 78.40% | | | 0.78(0.12) | | 0.7(0.0) | 0.66(0.04) | | 0.8(0.05) | 0.69 | 0.64 | 0.54 | 0.62 |
| 4 to 14 | 10144 | 29(0.3%) | | 44.10% | 78.40% | | | 0.92(0.01) | | 0.76(0.04) | 0.85(0.04) | | 0.85(0.06) | 0.83 | 0.69 | 0.77 | 0.73 |
| 5 to 7 | 13046 | 80(0.6%) | | 44.30% | 77.60% | | | 0.81(0.01) | | 0.74(0.0) | 0.78(0.04) | | 0.8(0.01) | 0.91 | 0.74 | 0.84 | 0.8 |
| 5 to 8 | 13028 | 78(0.6%) | | 44.40% | 77.60% | | | 0.79(0.01) | | 0.67(0.0) | 0.71(0.03) | | 0.81(0.01) | 0.82 | 0.8 | 0.84 | 0.76 |
| 5 to 9 | 13005 | 75(0.6%) | | 44.30% | 77.60% | | | 0.8(0.0) | | 0.69(0.0) | 0.65(0.04) | | 0.78(0.02) | 0.83 | 0.72 | 0.73 | 0.72 |
| 5 to 10 | 12966 | 72(0.6%) | | 44.30% | 77.60% | | | 0.8(0.01) | | 0.69(0.0) | 0.7(0.03) | | 0.76(0.02) | 0.85 | 0.7 | 0.77 | 0.77 |
| 5 to 11 | 12933 | 66(0.5%) | | 44.40% | 77.60% | | | 0.78(0.01) | | 0.67(0.02) | 0.57(0.03) | | 0.76(0.02) | 0.86 | 0.72 | 0.86 | 0.78 |
| 5 to 12 | 12883 | 56(0.4%) | | 44.40% | 77.60% | | | 0.78(0.03) | | 0.63(0.02) | 0.69(0.07) | | 0.76(0.05) | 0.78 | 0.63 | 0.81 | 0.76 |
| 5 to 13 | 12767 | 49(0.4%) | | 44.50% | 77.70% | | | 0.69(0.01) | | 0.69(0.03) | 0.6(0.08) | | 0.72(0.08) | 0.8 | 0.77 | 0.73 | 0.64 |
| 5 to 14 | 12528 | 41(0.3%) | | 44.50% | 77.60% | | | 0.75(0.01) | | 0.72(0.02) | 0.58(0.11) | | 0.7(0.07) | 0.88 | 0.73 | 0.73 | 0.71 |
| 6 to 8 | 14989 | 106(0.7%) | | 44.30% | 77.10% | | | 0.76(0.01) | | 0.65(0.02) | 0.65(0.02) | | 0.72(0.02) | 0.83 | 0.7 | 0.79 | 0.74 |
| 6 to 9 | 14964 | 102(0.7%) | | 44.20% | 77.10% | | | 0.82(0.01) | | 0.66(0.0) | 0.71(0.02) | | 0.78(0.03) | 0.81 | 0.68 | 0.81 | 0.72 |
| 6 to 10 | 14922 | 97(0.7%) | | 44.30% | 77.10% | | | 0.84(0.01) | | 0.76(0.01) | 0.71(0.08) | | 0.84(0.01) | 0.82 | 0.77 | 0.81 | 0.72 |
| 6 to 11 | 14883 | 89(0.6%) | | 44.30% | 77.10% | | | 0.77(0.01) | | 0.74(0.0) | 0.56(0.02) | | 0.76(0.02) | 0.86 | 0.72 | 0.8 | 0.73 |
| 6 to 12 | 14829 | 79(0.5%) | | 44.30% | 77.10% | | | 0.76(0.0) | | 0.71(0.0) | 0.65(0.05) | | 0.77(0.02) | 0.73 | 0.6 | 0.76 | 0.7 |
| 6 to 13 | 14695 | 70(0.5%) | | 44.40% | 77.20% | | | 0.79(0.0) | | 0.69(0.0) | 0.62(0.05) | | 0.78(0.01) | 0.86 | 0.71 | 0.82 | 0.78 |
| 6 to 14 | 14416 | 53(0.4%) | | 44.40% | | 77.10% | | 0.71(0.01) | | 0.7(0.03) | 0.6(0.04) | | 0.73(0.03) | 0.85 | 0.8 | 0.81 | 0.7 |
| 7 to 9 | 16761 | 126(0.8%) | | 44.20% | | 76.50% | | 0.82(0.0) | | 0.69(0.0) | 0.67(0.04) | | 0.76(0.03) | 0.79 | 0.72 | 0.78 | 0.72 |
| 7 to 10 | 16718 | 121(0.7%) | | 44.20% | | 76.40% | | 0.82(0.01) | | 0.62(0.0) | 0.68(0.03) | | 0.8(0.01) | 0.83 | 0.67 | 0.78 | 0.76 |
| 7 to 11 | 16675 | 113(0.7%) | | 44.20% | | 76.40% | | 0.76(0.01) | | 0.66(0.01) | 0.68(0.04) | | 0.75(0.02) | 0.8 | 0.66 | 0.76 | 0.72 |
| 7 to 12 | 16615 | 102(0.6%) | | 44.30% | | 76.40% | | 0.81(0.01) | | 0.65(0.01) | 0.7(0.03) | | 0.8(0.01) | 0.81 | 0.72 | 0.79 | 0.72 |
| 7 to 13 | 16470 | 92(0.6%) | | 44.30% | | 76.50% | | 0.72(0.01) | | 0.72(0.0) | 0.61(0.06) | | 0.77(0.02) | 0.8 | 0.72 | 0.74 | 0.74 |
| 7 to 14 | 16158 | 71(0.4%) | | 44.40% | | 76.50% | | 0.73(0.01) | | 0.69(0.0) | 0.68(0.07) | | 0.71(0.03) | 0.81 | 0.73 | 0.8 | 0.72 |
| 8 to 10 | 18553 | 149(0.8%) | | 44.30% | | 75.80% | | 0.78(0.01) | | 0.7(0.0) | 0.69(0.04) | | 0.81(0.02) | 0.73 | 0.66 | 0.77 | 0.72 |
| 8 to 11 | 18509 | 141(0.8%) | | 44.30% | | 75.80% | | 0.79(0.0) | | 0.67(0.0) | 0.65(0.01) | | 0.79(0.02) | 0.82 | 0.66 | 0.81 | 0.69 |
| 8 to 12 | 18445 | 127(0.7%) | | 44.40% | | 75.90% | | 0.84(0.01) | | 0.74(0.0) | 0.63(0.03) | | 0.79(0.02) | 0.77 | 0.69 | 0.79 | 0.68 |
| 8 to 13 | 18291 | 115(0.6%) | | 44.40% | | 75.90% | | 0.87(0.01) | | 0.71(0.01) | 0.69(0.03) | | 0.87(0.01) | 0.8 | 0.63 | 0.82 | 0.72 |
| 8 to 14 | 17956 | 91(0.5%) | | 44.50% | | 75.90% | | 0.85(0.01) | | 0.71(0.02) | 0.65(0.02) | | 0.84(0.03) | 0.85 | 0.71 | 0.8 | 0.7 |
| 9 to 11 | 20482 | 160(0.8%) | | 44.60% | | 75.40% | | 0.74(0.0) | | 0.64(0.01) | 0.67(0.03) | | 0.71(0.01) | 0.85 | 0.69 | 0.82 | 0.72 |
| 9 to 12 | 20415 | 144(0.7%) | | 44.60% | | 75.40% | | 0.77(0.01) | | 0.71(0.01) | 0.59(0.01) | | 0.77(0.02) | 0.81 | 0.71 | 0.81 | 0.71 |
| 9 to 13 | 20250 | 131(0.6%) | | 44.60% | | 75.50% | | 0.72(0.01) | | 0.68(0.01) | 0.72(0.03) | | 0.75(0.02) | 0.82 | 0.7 | 0.81 | 0.7 |
| 9 to 14 | 19888 | 103(0.5%) | | 44.70% | | 75.50% | | 0.78(0.01) | | 0.71(0.0) | 0.61(0.05) | | 0.75(0.02) | 0.78 | 0.65 | 0.77 | 0.72 |
| 10 to 12 | 22328 | 167(0.7%) | | 44.80% | | 75.10% | | 0.75(0.01) | | 0.72(0.01) | 0.63(0.03) | | 0.76(0.01) | 0.84 | 0.67 | 0.83 | 0.72 |
| 10 to 13 | 22162 | 153(0.7%) | | 44.80% | | 75.10% | | 0.84(0.01) | | 0.7(0.01) | 0.66(0.04) | | 0.81(0.01) | 0.81 | 0.68 | 0.83 | 0.69 |
| 10 to 14 | 21780 | 121(0.6%) | | 44.80% | | 75.10% | | 0.7(0.01) | | 0.7(0.01) | 0.52(0.02) | | 0.72(0.01) | 0.85 | 0.72 | 0.84 | 0.76 |
| 11 to 13 | 24076 | 178(0.7%) | | 45.00% | | 74.80% | | 0.85(0.01) | | 0.77(0.01) | 0.72(0.02) | | 0.83(0.01) | 0.79 | 0.7 | 0.79 | 0.67 |
| 11 to 14 | 23688 | 141(0.6%) | | 45.10% | | 74.80% | | 0.77(0.01) | | 0.69(0.02) | 0.61(0.06) | | 0.79(0.02) | 0.79 | 0.64 | 0.77 | 0.74 |
| 12 to 14 | 25421 | 157(0.6%) | | 45.30% | | 74.50% | | 0.82(0.02) | | 0.73(0.01) | 0.59(0.06) | | 0.87(0.01) | 0.78 | 0.69 | 0.77 | 0.67 |

* It was not reported because the corresponding cohort was removed due to lack of sample size.

**Supplementary Table 11.** Prediabetes cohorts statistics

| Ages (years) | Cohort size  (N) | | Cases | Sex %  Female | | Race %  White | Median Test AUC(SD) | | | | | Mean Cross Validation AUC | | | | | | | | |
| --- | --- | --- | --- | --- | --- | --- | --- | --- | --- | --- | --- | --- | --- | --- | --- | --- | --- | --- | --- | --- |
|  |  |  |  |  |  |  | DW-TSF-CNN | Random Forest | CRNN | Transformer | | DW-TSF-CNN | | | CRNN | | Transformer | | Random Forest | |
| 2 to 4 | 3921 | 438(11.2%) | | 43.00% | 0.00% | | 0.74(0.0) | 0.69(0.0) | 0.75(0.0) | | 0.7(0.02) | | 0.74 | 0.72 | | 0.71 | | 0.69 | |  |
| 2 to 5 | 3901 | 418(10.7%) | | 43.00% | 0.00% | | 0.77(0.01) | 0.69(0.0) | 0.71(0.02) | | 0.76(0.02) | | 0.75 | 0.74 | | 0.72 | | 0.7 | |  |
| 2 to 6 | 3880 | 397(10.2%) | | 43.00% | 0.00% | | 0.74(0.0) | 0.66(0.01) | 0.72(0.02) | | 0.69(0.02) | | 0.79 | 0.75 | | 0.76 | | 0.73 | |  |
| 2 to 7 | 3858 | 377(9.8%) | | 42.90% | 0.00% | | 0.78(0.01) | 0.74(0.01) | 0.77(0.01) | | 0.76(0.02) | | 0.73 | 0.7 | | 0.69 | | 0.7 | |  |
| 2 to 8 | 3824 | 349(9.1%) | | 43.00% | 0.00% | | 0.72(0.01) | 0.69(0.01) | 0.73(0.01) | | 0.72(0.01) | | 0.74 | 0.73 | | 0.72 | | 0.69 | |  |
| 2 to 9 | 3783 | 315(8.3%) | | 42.80% | 0.00% | | 0.76(0.01) | 0.72(0.01) | 0.74(0.01) | | 0.71(0.02) | | 0.75 | 0.74 | | 0.68 | | 0.68 | |  |
| 2 to 10 | 3740 | 286(7.6%) | | 42.90% | 0.00% | | 0.71(0.0) | 0.71(0.01) | 0.67(0.01) | | 0.66(0.02) | | 0.74 | 0.71 | | 0.67 | | 0.67 | |  |
| 2 to 11 | 3707 | 261(7.0%) | | 42.90% | 0.00% | | 0.75(0.01) | 0.68(0.01) | 0.68(0.01) | | 0.73(0.02) | | 0.7 | 0.69 | | 0.66 | | 0.7 | |  |
| 2 to 12 | 3651 | 216(5.9%) | | 43.00% | 0.00% | | 0.7(0.01) | 0.63(0.01) | 0.65(0.02) | | 0.68(0.03) | | 0.71 | 0.65 | | 0.7 | | 0.61 | |  |
| 2 to 13 | 3589 | 174(4.8%) | | 43.00% | 0.00% | | 0.63(0.01) | 0.64(0.01) | 0.55(0.01) | | 0.66(0.03) | | 0.7 | 0.69 | | 0.67 | | 0.67 | |  |
| 3 to 5 | 8194 | 933(11.4%) | | 43.60% | 0.00% | | 0.73(0.0) | 0.67(0.01) | 0.7(0.02) | | 0.7(0.01) | | 0.76 | 0.71 | | 0.73 | | 0.68 | |  |
| 3 to 6 | 8159 | 898(11.0%) | | 43.60% | 0.00% | | 0.76(0.0) | 0.7(0.0) | 0.69(0.02) | | 0.74(0.01) | | 0.76 | 0.7 | | 0.71 | | 0.69 | |  |
| 3 to 7 | 8123 | 865(10.6%) | | 43.60% | 0.00% | | 0.76(0.0) | 0.71(0.0) | 0.69(0.02) | | 0.72(0.01) | | 0.76 | 0.7 | | 0.72 | | 0.7 | |  |
| 3 to 8 | 8052 | 806(10.0%) | | 43.50% | 0.00% | | 0.74(0.0) | 0.68(0.01) | 0.68(0.02) | | 0.71(0.01) | | 0.77 | 0.72 | | 0.74 | | 0.7 | |  |
| 3 to 9 | 7971 | 740(9.3%) | | 43.40% | 0.00% | | 0.74(0.0) | 0.67(0.0) | 0.66(0.01) | | 0.7(0.01) | | 0.75 | 0.72 | | 0.74 | | 0.7 | |  |
| 3 to 10 | 7880 | 671(8.5%) | | 43.50% | 0.00% | | 0.76(0.0) | 0.69(0.0) | 0.7(0.02) | | 0.74(0.01) | | 0.73 | 0.66 | | 0.68 | | 0.65 | |  |
| 3 to 11 | 7790 | 601(7.7%) | | 43.50% | 0.00% | | 0.73(0.0) | 0.68(0.01) | 0.63(0.0) | | 0.72(0.01) | | 0.74 | 0.7 | | 0.7 | | 0.68 | |  |
| 3 to 12 | 7671 | 508(6.6%) | | 43.60% | 0.00% | | 0.72(0.01) | 0.64(0.01) | 0.66(0.01) | | 0.66(0.02) | | 0.76 | 0.66 | | 0.71 | | 0.67 | |  |
| 3 to 13 | 7499 | 402(5.4%) | | 43.50% | 0.00% | | 0.69(0.01) | 0.63(0.02) | 0.66(0.02) | | 0.64(0.02) | | 0.69 | 0.63 | | 0.64 | | 0.66 | |  |
| 4 to 6 | 11038 | 1218(11.0%) | | 44.00% | 0.00% | | 0.75(0.0) | 0.68(0.0) | 0.7(0.02) | | 0.73(0.01) | | 0.75 | 0.69 | | 0.73 | | 0.69 | |  |
| 4 to 7 | 10994 | 1177(10.7%) | | 44.00% | 0.00% | | 0.74(0.0) | 0.69(0.01) | 0.69(0.01) | | 0.74(0.01) | | 0.77 | 0.71 | | 0.75 | | 0.71 | |  |
| 4 to 8 | 10912 | 1109(10.2%) | | 44.00% | 0.00% | | 0.76(0.01) | 0.69(0.0) | 0.69(0.04) | | 0.73(0.01) | | 0.77 | 0.72 | | 0.74 | | 0.71 | |  |
| 4 to 9 | 10810 | 1026(9.5%) | | 43.90% | 0.00% | | 0.75(0.0) | 0.69(0.01) | 0.68(0.02) | | 0.73(0.01) | | 0.75 | 0.67 | | 0.72 | | 0.69 | |  |
| 4 to 10 | 10696 | 941(8.8%) | | 44.00% | 0.00% | | 0.75(0.0) | 0.66(0.01) | 0.66(0.01) | | 0.74(0.01) | | 0.75 | 0.67 | | 0.72 | | 0.69 | |  |
| 4 to 11 | 10573 | 841(8.0%) | | 44.00% | 0.00% | | 0.72(0.0) | 0.67(0.0) | 0.63(0.0) | | 0.68(0.01) | | 0.75 | 0.69 | | 0.72 | | 0.69 | |  |
| 4 to 12 | 10417 | 716(6.9%) | | 44.00% | 0.00% | | 0.73(0.0) | 0.66(0.0) | 0.64(0.0) | | 0.71(0.01) | | 0.76 | 0.67 | | 0.71 | | 0.7 | |  |
| 4 to 13 | 10194 | 580(5.7%) | | 44.00% | 0.00% | | 0.73(0.01) | 0.67(0.0) | 0.66(0.01) | | 0.69(0.02) | | 0.71 | 0.64 | | 0.66 | | 0.66 | |  |
| 5 to 7 | 13541 | 1432(10.6%) | | 44.50% | 0.00% | | 0.73(0.0) | 0.67(0.0) | 0.64(0.01) | | 0.71(0.01) | | 0.74 | 0.64 | | 0.72 | | 0.68 | |  |
| 5 to 8 | 13450 | 1355(10.1%) | | 44.40% | 0.00% | | 0.75(0.0) | 0.69(0.0) | 0.65(0.01) | | 0.72(0.01) | | 0.75 | 0.66 | | 0.71 | | 0.67 | |  |
| 5 to 9 | 13333 | 1258(9.4%) | | 44.40% | 0.00% | | 0.74(0.0) | 0.68(0.0) | 0.65(0.01) | | 0.72(0.01) | | 0.73 | 0.67 | | 0.7 | | 0.66 | |  |
| 5 to 10 | 13196 | 1157(8.8%) | | 44.40% | 0.00% | | 0.75(0.0) | 0.68(0.0) | 0.65(0.02) | | 0.72(0.01) | | 0.74 | 0.67 | | 0.71 | | 0.68 | |  |
| 5 to 11 | 13058 | 1046(8.0%) | | 44.40% | 0.00% | | 0.74(0.0) | 0.68(0.0) | 0.65(0.02) | | 0.72(0.01) | | 0.75 | 0.67 | | 0.72 | | 0.68 | |  |
| 5 to 12 | 12867 | 893(6.9%) | | 44.40% | 0.00% | | 0.69(0.01) | 0.64(0.01) | 0.61(0.01) | | 0.66(0.01) | | 0.72 | 0.64 | | 0.7 | | 0.67 | |  |
| 5 to 13 | 12601 | 732(5.8%) | | 44.40% | 0.00% | | 0.72(0.01) | 0.67(0.01) | 0.61(0.01) | | 0.68(0.01) | | 0.73 | 0.64 | | 0.66 | | 0.65 | |  |
| 6 to 8 | 15473 | 1578(10.2%) | | 44.30% | 0.00% | | 0.74(0.0) | 0.69(0.0) | 0.68(0.01) | | 0.71(0.0) | | 0.73 | 0.65 | | 0.7 | | 0.67 | |  |
| 6 to 9 | 15341 | 1467(9.6%) | | 44.30% | 0.00% | | 0.75(0.0) | 0.68(0.0) | 0.66(0.0) | | 0.72(0.01) | | 0.74 | 0.66 | | 0.71 | | 0.68 | |  |
| 6 to 10 | 15190 | 1353(8.9%) | | 44.30% | 0.00% | | 0.72(0.0) | 0.65(0.0) | 0.65(0.01) | | 0.7(0.01) | | 0.75 | 0.65 | | 0.73 | | 0.68 | |  |
| 6 to 11 | 15032 | 1226(8.2%) | | 44.30% | 0.00% | | 0.75(0.0) | 0.66(0.0) | 0.65(0.01) | | 0.72(0.01) | | 0.74 | 0.67 | | 0.71 | | 0.68 | |  |
| 6 to 12 | 14816 | 1052(7.1%) | | 44.30% | 0.00% | | 0.73(0.0) | 0.68(0.0) | 0.64(0.01) | | 0.7(0.01) | | 0.72 | 0.65 | | 0.69 | | 0.67 | |  |
| 6 to 13 | 14516 | 871(6.0%) | | 44.30% | 0.00% | | 0.71(0.01) | 0.66(0.01) | 0.65(0.0) | | 0.66(0.01) | | 0.72 | 0.63 | | 0.68 | | 0.65 | |  |
| 7 to 9 | 17190 | 1670(9.7%) | | 44.30% | 0.00% | | 0.75(0.0) | 0.67(0.0) | 0.66(0.01) | | 0.72(0.01) | | 0.74 | 0.7 | | 0.7 | | 0.68 | |  |
| 7 to 10 | 17025 | 1543(9.1%) | | 44.30% | 0.00% | | 0.76(0.0) | 0.68(0.0) | 0.68(0.0) | | 0.72(0.01) | | 0.73 | 0.67 | | 0.7 | | 0.67 | |  |
| 7 to 11 | 16850 | 1403(8.3%) | | 44.30% | 0.00% | | 0.72(0.0) | 0.65(0.0) | 0.63(0.0) | | 0.69(0.01) | | 0.73 | 0.66 | | 0.71 | | 0.66 | |  |
| 7 to 12 | 16613 | 1213(7.3%) | | 44.30% | 0.00% | | 0.72(0.0) | 0.67(0.01) | 0.62(0.0) | | 0.69(0.01) | | 0.73 | 0.64 | | 0.68 | | 0.67 | |  |
| 7 to 13 | 16284 | 1013(6.2%) | | 44.30% | 0.00% | | 0.69(0.0) | 0.64(0.0) | 0.61(0.0) | | 0.68(0.01) | | 0.72 | 0.63 | | 0.69 | | 0.66 | |  |
| 8 to 10 | 18902 | 1733(9.2%) | | 44.40% | 0.00% | | 0.74(0.0) | 0.66(0.0) | 0.64(0.0) | | 0.69(0.01) | | 0.72 | 0.66 | | 0.69 | | 0.65 | |  |
| 8 to 11 | 18714 | 1581(8.4%) | | 44.40% | 0.00% | | 0.73(0.01) | 0.65(0.0) | 0.64(0.0) | | 0.69(0.01) | | 0.73 | 0.66 | | 0.71 | | 0.68 | |  |
| 8 to 12 | 18464 | 1379(7.5%) | | 44.40% | 0.00% | | 0.72(0.0) | 0.66(0.01) | 0.62(0.01) | | 0.69(0.01) | | 0.7 | 0.64 | | 0.66 | | 0.64 | |  |
| 8 to 13 | 18113 | 1164(6.4%) | | 44.40% | 0.00% | | 0.74(0.0) | 0.67(0.0) | 0.64(0.0) | | 0.71(0.0) | | 0.71 | 0.62 | | 0.67 | | 0.64 | |  |
| 9 to 11 | 20719 | 1747(8.4%) | | 44.60% | 0.00% | | 0.71(0.0) | 0.65(0.0) | 0.64(0.0) | | 0.68(0.01) | | 0.73 | 0.65 | | 0.71 | | 0.67 | |  |
| 9 to 12 | 20450 | 1526(7.5%) | | 44.60% | 0.00% | | 0.74(0.0) | 0.68(0.0) | 0.66(0.0) | | 0.72(0.01) | | 0.71 | 0.64 | | 0.69 | | 0.66 | |  |
| 9 to 13 | 20073 | 1295(6.5%) | | 44.60% | 0.00% | | 0.7(0.0) | 0.65(0.0) | 0.63(0.0) | | 0.68(0.01) | | 0.73 | 0.63 | | 0.69 | | 0.66 | |  |
| 10 to 12 | 22375 | 1653(7.4%) | | 44.70% | 0.00% | | 0.73(0.0) | 0.67(0.0) | 0.63(0.01) | | 0.69(0.01) | | 0.7 | 0.62 | | 0.66 | | 0.65 | |  |
| 10 to 13 | 21980 | 1404(6.4%) | | 44.70% | 0.00% | | 0.74(0.0) | 0.67(0.0) | 0.64(0.0) | | 0.68(0.01) | | 0.71 | 0.61 | | 0.65 | | 0.66 | |  |
| 11 to 13 | 23917 | 1554(6.5%) | | 45.00% | 75.10% | | 0.74(0.0) | 0.66(0.0) | 0.65(0.01) | | 0.69(0.01) | | 0.73 | 0.63 | | 0.66 | | 0.66 | |  |

**Supplementary Table 12.** Metabolic syndrome cohort statistics

| Ages (years) | Cohort size  (N) | | Cases | Sex %  Female | | Race %  White | Median Test AUC(SD) | | | | | Mean Cross Validation AUC | | | | | | | | |
| --- | --- | --- | --- | --- | --- | --- | --- | --- | --- | --- | --- | --- | --- | --- | --- | --- | --- | --- | --- | --- |
|  |  |  |  |  |  |  | DW-TSF-CNN | Random Forest | CRNN | Transformer | | DW-TSF-CNN | | | CRNN | | Transformer | | Random Forest | |
| 2 to 4 | 3877 | 646(16.7%) | | 43.20% | 83.50% | | 0.6(0.0) | 0.59(0.0) | 0.54(0.01) | | 0.6(0.04) | | 0.65 | 0.55 | | 0.6 | | 0.59 | |  |
| 2 to 5 | 3875 | 644(16.6%) | | 43.20% | 83.50% | | 0.66(0.01) | 0.64(0.01) | 0.56(0.02) | | 0.67(0.01) | | 0.67 | 0.58 | | 0.61 | | 0.62 | |  |
| 2 to 6 | 3871 | 640(16.5%) | | 43.20% | 83.50% | | 0.66(0.0) | 0.61(0.01) | 0.57(0.01) | | 0.62(0.01) | | 0.68 | 0.54 | | 0.62 | | 0.63 | |  |
| 2 to 7 | 3866 | 637(16.5%) | | 43.10% | 83.50% | | 0.72(0.01) | 0.64(0.01) | 0.66(0.05) | | 0.65(0.01) | | 0.73 | 0.66 | | 0.7 | | 0.65 | |  |
| 2 to 8 | 3856 | 633(16.4%) | | 43.10% | 83.50% | | 0.67(0.01) | 0.65(0.0) | 0.58(0.06) | | 0.69(0.01) | | 0.66 | 0.56 | | 0.69 | | 0.64 | |  |
| 2 to 9 | 3847 | 631(16.4%) | | 43.00% | 83.50% | | 0.71(0.01) | 0.67(0.0) | 0.66(0.04) | | 0.72(0.01) | | 0.73 | 0.69 | | 0.72 | | 0.67 | |  |
| 2 to 10 | 3831 | 629(16.4%) | | 43.00% | 83.50% | | 0.79(0.0) | 0.68(0.0) | 0.74(0.06) | | 0.76(0.01) | | 0.76 | 0.62 | | 0.72 | | 0.67 | |  |
| 2 to 11 | 3812 | 618(16.2%) | | 43.00% | 83.60% | | 0.78(0.0) | 0.68(0.01) | 0.67(0.05) | | 0.74(0.01) | | 0.76 | 0.7 | | 0.73 | | 0.67 | |  |
| 2 to 12 | 3463 | 279(8.1%) | | 43.20% | 83.80% | | 0.7(0.01) | 0.63(0.01) | 0.52(0.02) | | 0.62(0.02) | | 0.7 | 0.55 | | 0.63 | | 0.63 | |  |
| 2 to 13 | 3355 | 191(5.7%) | | 43.00% | 84.00% | | 0.64(0.01) | 0.61(0.02) | 0.49(0.06) | | 0.55(0.06) | | 0.64 | 0.56 | | 0.54 | | 0.55 | |  |
| 3 to 5 | 8152 | 1446(17.7%) | | 43.60% | 79.30% | | 0.65(0.0) | 0.61(0.0) | 0.56(0.03) | | 0.62(0.01) | | 0.68 | 0.57 | | 0.62 | | 0.62 | |  |
| 3 to 6 | 8146 | 1440(17.7%) | | 43.60% | 79.30% | | 0.72(0.0) | 0.65(0.0) | 0.57(0.0) | | 0.67(0.01) | | 0.7 | 0.58 | | 0.63 | | 0.63 | |  |
| 3 to 7 | 8138 | 1435(17.6%) | | 43.60% | 79.30% | | 0.71(0.0) | 0.65(0.0) | 0.68(0.05) | | 0.71(0.0) | | 0.72 | 0.63 | | 0.7 | | 0.65 | |  |
| 3 to 8 | 8116 | 1426(17.6%) | | 43.50% | 79.30% | | 0.76(0.0) | 0.69(0.0) | 0.7(0.07) | | 0.75(0.01) | | 0.74 | 0.7 | | 0.71 | | 0.67 | |  |
| 3 to 9 | 8095 | 1420(17.5%) | | 43.50% | 79.30% | | 0.72(0.01) | 0.69(0.0) | 0.68(0.02) | | 0.72(0.0) | | 0.72 | 0.65 | | 0.72 | | 0.67 | |  |
| 3 to 10 | 8055 | 1402(17.4%) | | 43.50% | 79.30% | | 0.74(0.0) | 0.67(0.01) | 0.68(0.04) | | 0.71(0.01) | | 0.75 | 0.7 | | 0.74 | | 0.68 | |  |
| 3 to 11 | 8010 | 1377(17.2%) | | 43.40% | 79.40% | | 0.78(0.0) | 0.71(0.0) | 0.74(0.03) | | 0.74(0.01) | | 0.76 | 0.71 | | 0.74 | | 0.69 | |  |
| 3 to 12 | 7258 | 649(8.9%) | | 43.70% | 80.10% | | 0.71(0.01) | 0.65(0.01) | 0.54(0.02) | | 0.67(0.01) | | 0.67 | 0.58 | | 0.64 | | 0.62 | |  |
| 3 to 13 | 6993 | 451(6.4%) | | 43.60% | 80.30% | | 0.68(0.01) | 0.62(0.01) | 0.51(0.02) | | 0.59(0.01) | | 0.65 | 0.52 | | 0.55 | | 0.61 | |  |
| 4 to 6 | 11041 | 1986(18.0%) | | 43.90% | 78.10% | | 0.69(0.0) | 0.63(0.0) | 0.56(0.03) | | 0.65(0.0) | | 0.7 | 0.59 | | 0.68 | | 0.64 | |  |
| 4 to 7 | 11032 | 1980(17.9%) | | 43.90% | 78.10% | | 0.71(0.0) | 0.65(0.0) | 0.66(0.02) | | 0.67(0.01) | | 0.74 | 0.7 | | 0.71 | | 0.66 | |  |
| 4 to 8 | 11004 | 1968(17.9%) | | 43.90% | 78.10% | | 0.74(0.0) | 0.69(0.0) | 0.7(0.01) | | 0.73(0.01) | | 0.73 | 0.67 | | 0.72 | | 0.67 | |  |
| 4 to 9 | 10973 | 1956(17.8%) | | 43.90% | 78.10% | | 0.75(0.0) | 0.68(0.0) | 0.69(0.02) | | 0.73(0.01) | | 0.74 | 0.72 | | 0.72 | | 0.68 | |  |
| 4 to 10 | 10918 | 1930(17.7%) | | 43.80% | 78.10% | | 0.73(0.0) | 0.69(0.0) | 0.7(0.02) | | 0.74(0.01) | | 0.74 | 0.72 | | 0.74 | | 0.7 | |  |
| 4 to 11 | 10864 | 1899(17.5%) | | 43.80% | 78.20% | | 0.76(0.0) | 0.69(0.0) | 0.72(0.05) | | 0.76(0.0) | | 0.77 | 0.69 | | 0.75 | | 0.69 | |  |
| 4 to 12 | 9845 | 910(9.2%) | | 43.90% | 78.80% | | 0.68(0.0) | 0.64(0.01) | 0.51(0.02) | | 0.64(0.01) | | 0.7 | 0.61 | | 0.63 | | 0.63 | |  |
| 4 to 13 | 9494 | 647(6.8%) | | 44.00% | 79.00% | | 0.64(0.0) | 0.61(0.0) | 0.52(0.01) | | 0.58(0.02) | | 0.68 | 0.56 | | 0.66 | | 0.63 | |  |
| 5 to 7 | 13589 | 2463(18.1%) | | 44.40% | 77.40% | | 0.71(0.0) | 0.64(0.0) | 0.65(0.03) | | 0.67(0.01) | | 0.71 | 0.61 | | 0.67 | | 0.65 | |  |
| 5 to 8 | 13559 | 2449(18.1%) | | 44.40% | 77.40% | | 0.73(0.0) | 0.66(0.0) | 0.67(0.05) | | 0.7(0.0) | | 0.74 | 0.68 | | 0.7 | | 0.67 | |  |
| 5 to 9 | 13524 | 2434(18.0%) | | 44.30% | 77.40% | | 0.73(0.0) | 0.67(0.0) | 0.69(0.04) | | 0.72(0.01) | | 0.73 | 0.67 | | 0.72 | | 0.68 | |  |
| 5 to 10 | 13457 | 2403(17.9%) | | 44.30% | 77.40% | | 0.74(0.0) | 0.67(0.0) | 0.7(0.01) | | 0.72(0.0) | | 0.76 | 0.67 | | 0.74 | | 0.69 | |  |
| 5 to 11 | 13396 | 2369(17.7%) | | 44.20% | 77.50% | | 0.73(0.0) | 0.68(0.0) | 0.72(0.01) | | 0.74(0.0) | | 0.75 | 0.73 | | 0.75 | | 0.69 | |  |
| 5 to 12 | 12157 | 1167(9.6%) | | 44.40% | 78.00% | | 0.66(0.01) | 0.61(0.01) | 0.52(0.01) | | 0.63(0.01) | | 0.69 | 0.58 | | 0.65 | | 0.64 | |  |
| 5 to 13 | 11734 | 850(7.2%) | | 44.40% | 78.20% | | 0.66(0.01) | 0.63(0.01) | 0.49(0.02) | | 0.61(0.02) | | 0.64 | 0.54 | | 0.57 | | 0.62 | |  |
| 6 to 8 | 15611 | 2857(18.3%) | | 44.20% | 76.80% | | 0.72(0.0) | 0.64(0.0) | 0.68(0.04) | | 0.69(0.0) | | 0.73 | 0.65 | | 0.71 | | 0.67 | |  |
| 6 to 9 | 15570 | 2838(18.2%) | | 44.20% | 76.90% | | 0.74(0.0) | 0.67(0.0) | 0.69(0.03) | | 0.71(0.0) | | 0.74 | 0.68 | | 0.72 | | 0.67 | |  |
| 6 to 10 | 15501 | 2806(18.1%) | | 44.20% | 76.80% | | 0.73(0.0) | 0.66(0.0) | 0.71(0.03) | | 0.72(0.0) | | 0.76 | 0.68 | | 0.74 | | 0.69 | |  |
| 6 to 11 | 15429 | 2765(17.9%) | | 44.10% | 76.90% | | 0.75(0.0) | 0.68(0.0) | 0.71(0.01) | | 0.73(0.0) | | 0.77 | 0.73 | | 0.75 | | 0.69 | |  |
| 6 to 12 | 14014 | 1391(9.9%) | | 44.40% | 77.50% | | 0.64(0.0) | 0.62(0.0) | 0.53(0.0) | | 0.63(0.01) | | 0.68 | 0.57 | | 0.66 | | 0.65 | |  |
| 6 to 13 | 13522 | 1020(7.5%) | | 44.50% | 77.70% | | 0.58(0.01) | 0.57(0.0) | 0.51(0.01) | | 0.56(0.01) | | 0.67 | 0.54 | | 0.64 | | 0.62 | |  |
| 7 to 9 | 17444 | 3213(18.4%) | | 44.20% | 76.10% | | 0.74(0.0) | 0.67(0.0) | 0.69(0.03) | | 0.7(0.0) | | 0.73 | 0.69 | | 0.71 | | 0.66 | |  |
| 7 to 10 | 17371 | 3178(18.3%) | | 44.20% | 76.10% | | 0.76(0.0) | 0.68(0.0) | 0.72(0.05) | | 0.74(0.0) | | 0.74 | 0.68 | | 0.72 | | 0.68 | |  |
| 7 to 11 | 17292 | 3134(18.1%) | | 44.20% | 76.20% | | 0.76(0.0) | 0.68(0.0) | 0.72(0.01) | | 0.74(0.0) | | 0.75 | 0.69 | | 0.74 | | 0.68 | |  |
| 7 to 12 | 15723 | 1611(10.2%) | | 44.40% | 76.70% | | 0.66(0.01) | 0.64(0.01) | 0.53(0.01) | | 0.63(0.01) | | 0.67 | 0.56 | | 0.62 | | 0.62 | |  |
| 7 to 13 | 15163 | 1182(7.8%) | | 44.40% | 76.90% | | 0.6(0.01) | 0.59(0.01) | 0.53(0.01) | | 0.6(0.02) | | 0.63 | 0.57 | | 0.62 | | 0.61 | |  |
| 8 to 10 | 19282 | 3556(18.4%) | | 44.30% | 75.50% | | 0.73(0.0) | 0.66(0.0) | 0.7(0.03) | | 0.71(0.0) | | 0.74 | 0.7 | | 0.73 | | 0.68 | |  |
| 8 to 11 | 19197 | 3507(18.3%) | | 44.30% | 75.50% | | 0.75(0.0) | 0.67(0.0) | 0.71(0.01) | | 0.73(0.0) | | 0.75 | 0.72 | | 0.73 | | 0.68 | |  |
| 8 to 12 | 17466 | 1823(10.4%) | | 44.50% | 76.10% | | 0.67(0.01) | 0.65(0.0) | 0.55(0.02) | | 0.63(0.01) | | 0.66 | 0.57 | | 0.65 | | 0.64 | |  |
| 8 to 13 | 16841 | 1336(7.9%) | | 44.50% | 76.30% | | 0.68(0.0) | 0.61(0.01) | 0.55(0.01) | | 0.64(0.01) | | 0.68 | 0.54 | | 0.62 | | 0.62 | |  |
| 9 to 11 | 21232 | 3866(18.2%) | | 44.50% | 75.20% | | 0.74(0.0) | 0.68(0.0) | 0.71(0.01) | | 0.72(0.0) | | 0.75 | 0.72 | | 0.73 | | 0.69 | |  |
| 9 to 12 | 19351 | 2033(10.5%) | | 44.70% | 75.70% | | 0.67(0.0) | 0.61(0.0) | 0.56(0.01) | | 0.64(0.01) | | 0.69 | 0.56 | | 0.63 | | 0.64 | |  |
| 9 to 13 | 18675 | 1505(8.1%) | | 44.80% | 75.80% | | 0.67(0.0) | 0.63(0.0) | 0.54(0.01) | | 0.62(0.0) | | 0.66 | 0.54 | | 0.61 | | 0.63 | |  |
| 10 to 12 | 21184 | 2263(10.7%) | | 44.90% | 75.30% | | 0.68(0.0) | 0.63(0.0) | 0.56(0.0) | | 0.63(0.0) | | 0.69 | 0.58 | | 0.63 | | 0.64 | |  |
| 10 to 13 | 20454 | 1681(8.2%) | | 44.90% | 75.50% | | 0.67(0.0) | 0.61(0.0) | 0.55(0.01) | | 0.62(0.01) | | 0.67 | 0.56 | | 0.61 | | 0.61 | |  |
| 11 to 13 | 22201 | 1840(8.3%) | | 45.20% | 75.10% | | 0.66(0.0) | 0.6(0.0) | 0.55(0.02) | | 0.59(0.01) | | 0.67 | 0.55 | | 0.63 | | 0.61 | |  |


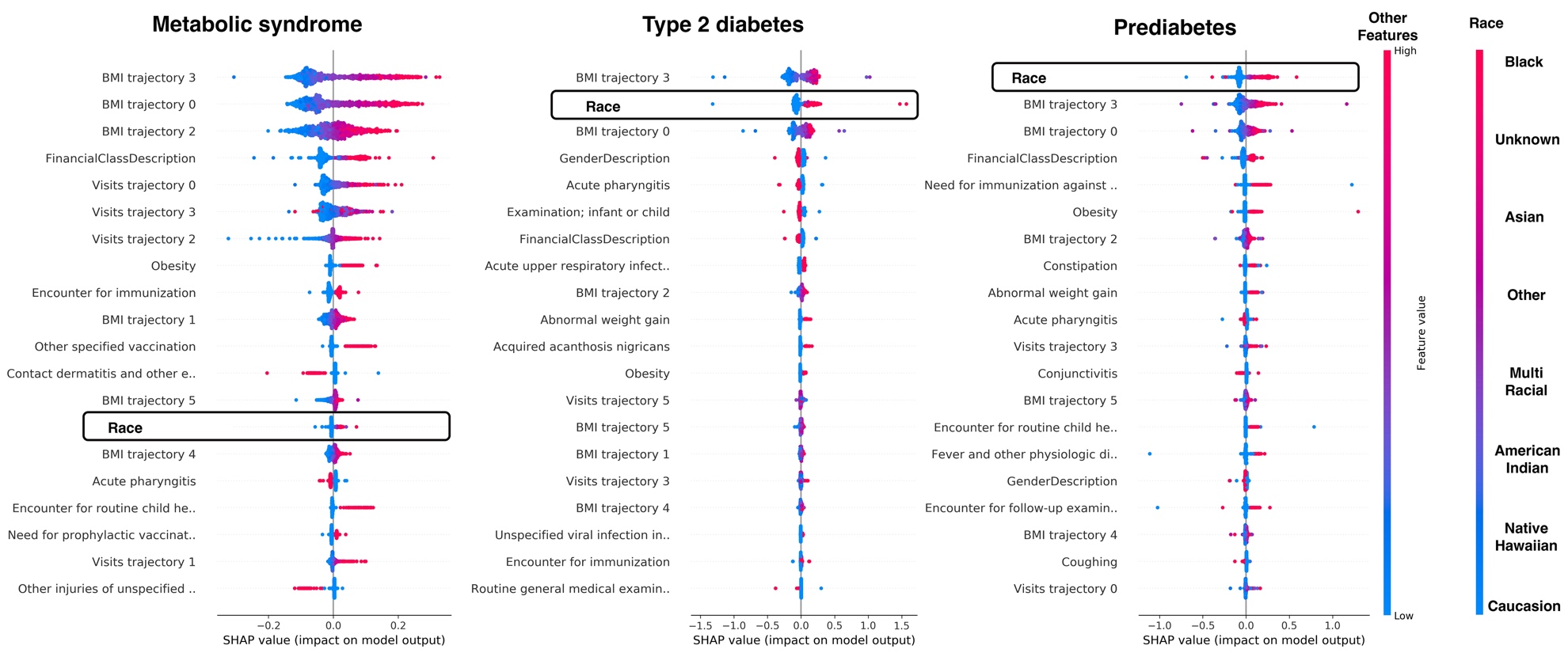


Supplementary Figure 6: Impact of specific racial categories on disease development in the DW-TSF-CNN model. While Figure 4 in the main paper provides an overall SHAP summary plot, this particular figure focuses on a narrower observation window of 6 to 9 years old to examine model decisions in more detail. To facilitate analysis, races are sorted based on their decreasing likelihood of a positive prediction, as indicated by the Race legend. In the plot, Caucasian individuals are represented by blue dots, while Black individuals are represented by red dots. Across all three subplots for metabolic syndrome, type 2 diabetes, and prediabetes, blue dots consistently exhibit negative SHAP values, indicating a decreased likelihood of a positive prediction. Conversely, red dots (representing Black individuals) generally display positive SHAP values, indicating an increased likelihood of a positive prediction, with a few exceptions. Multi racial and American Indian individuals (purple dots) can be seen around the middle in each disease with SHAP values around zero which indicates they had minimal impact on the model’s decision. The same pattern was seen in other observation windows.

Supplementary Figure 7: Mean Test AUC of DW-TSF-CNN across racial categories.

**Supplementary Table 13.** Embedding size of DW-TSF-CNNs.

| Cohort Name | BMI trajectory | | | | | | | | Visits trajectory | | | | | | | | | | | | | Concatenation | | | | |
| --- | --- | --- | --- | --- | --- | --- | --- | --- | --- | --- | --- | --- | --- | --- | --- | --- | --- | --- | --- | --- | --- | --- | --- | --- | --- | --- |
|  | Conv 1D | | Conv 1D | Conv 1D | |  | | | | Conv 1D | | | Conv 1D | | | Conv 1D | | |  | | | | Dense | Dense | Dense | |
| T2D_10_12 | (3, 1, 128) | | (3, 128, 512) | | (3, 512, 128) | | _ | | | | (1, 1, 512) | | | (1, 512, 8) | | | (3, 8, 128) | | | _ | | | (696, 100) | (100, 10) | | (10, 2) |
| T2D_10_13 | (3, 1, 32) | | (3, 32, 512) | | (1, 512, 8) | | _ | | | | (1, 1, 8) | | | (1, 8, 128) | | | (1, 128, 8) | | | _ | | | (4416, 100) | (100, 100) | | (100, 2) |
| T2D_10_14 | (3, 1, 128) | | (3, 128, 512) | | (3, 512, 128) | | _ | | | | (1, 1, 512) | | | (1, 512, 8) | | | (3, 8, 128) | | | _ | | | (696, 100) | (100, 10) | | (10, 2) |
| T2D_11_13 | (3, 1, 128) | | (3, 128, 64) | | (3, 64, 512) | | _ | | | | (3, 1, 8) | | | (1, 8, 512) | | | (3, 512, 512) | | | _ | | | (1068, 100) | (100, 100) | | (100, 2) |
| T2D_11_14 | (3, 1, 32) | | (3, 32, 512) | | (1, 512, 8) | | _ | | | | (1, 1, 8) | | | (1, 8, 128) | | | (1, 128, 8) | | | _ | | | (4416, 100) | (100, 100) | | (100, 2) |
| T2D_12_14 | (1, 1, 64) | | (3, 64, 512) | | (1, 512, 128) | | _ | | | | (1, 1, 32) | | | (3, 32, 512) | | | (1, 512, 128) | | | _ | | | (4656, 100) | (100, 100) | | (100, 2) |
| T2D_3_10 | (3, 1, 128) | | (3, 128, 512) | | (3, 512, 128) | | _ | | | | (1, 1, 512) | | | (1, 512, 8) | | | (3, 8, 128) | | | _ | | | (696, 100) | (100, 10) | | (10, 2) |
| T2D_3_11 | (3, 1, 128) | (3, 128, 512) | | | (3, 512, 128) | | | _ | | | | (1, 1, 512) | | | (1, 512, 8) | | | (3, 8, 128) | | | _ | | (696, 100) | (100, 10) | | (10, 2) |
| T2D_3_12 | (3, 1, 32) | (1, 32, 128) | | | (3, 128, 128) | | | _ | | | | (1, 1, 8) | | | (1, 8, 8) | | | (3, 8, 128) | | | _ | | (300, 100) | (100, 100) | | (100, 2) |
| T2D_3_13 | (3, 1, 32) | (3, 32, 32) | | | (1, 32, 512) | | | _ | | | | (1, 1, 8) | | | (3, 8, 128) | | | (1, 128, 512) | | | _ | | (5424, 100) | (100, 100) | | (100, 2) |
| T2D_3_14 | (3, 1, 128) | (3, 128, 64) | | | (1, 64, 8) | | | _ | | | | (1, 1, 64) | | | (3, 64, 128) | | | (1, 128, 8) | | | _ | | (456, 100) | (100, 10) | | (10, 2) |
| T2D_3_5 | (3, 1, 128) | (3, 128, 512) | | | (3, 512, 128) | | | _ | | | | (1, 1, 512) | | | (1, 512, 8) | | | (3, 8, 128) | | | _ | | (696, 100) | (100, 10) | | (10, 2) |
| T2D_3_6 | (3, 1, 32) | (3, 32, 512) | | | (1, 512, 8) | | | _ | | | | (1, 1, 8) | | | (1, 8, 128) | | | (1, 128, 8) | | | _ | | (4416, 100) | (100, 100) | | (100, 2) |
| T2D_3_7 | (1, 1, 512) | (3, 512, 512) | | | (3, 512, 128) | | | _ | | | | (1, 1, 8) | | | (3, 8, 512) | | | (3, 512, 128) | | | _ | | (300, 10) | (10, 10) | | (10, 2) |
| T2D_3_8 | (3, 1, 128) | (3, 128, 512) | | | (3, 512, 128) | | | _ | | | | (1, 1, 512) | | | (1, 512, 8) | | | (3, 8, 128) | | | _ | | (696, 100) | (100, 10) | | (10, 2) |
| T2D_3_9 | (3, 1, 128) | (3, 128, 64) | | | (3, 64, 512) | | | _ | | | | (3, 1, 8) | | | (1, 8, 512) | | | (3, 512, 512) | | | _ | | (1068, 100) | (100, 100) | | (100, 2) |
| T2D_4_10 | (3, 1, 128) | (3, 128, 512) | | | (3, 512, 128) | | | _ | | | | (1, 1, 512) | | | (1, 512, 8) | | | (3, 8, 128) | | | _ | | (696, 100) | (100, 10) | | (10, 2) |
| T2D_4_11 | (3, 1, 128) | (3, 128, 512) | | | (3, 512, 128) | | | _ | | | | (1, 1, 512) | | | (1, 512, 8) | | | (3, 8, 128) | | | _ | | (696, 100) | (100, 10) | | (10, 2) |
| T2D_4_12 | (1, 1, 8) | (3, 8, 64) | | | (3, 64, 8) | | | _ | | | | (3, 1, 32) | | | (3, 32, 8) | | | (3, 8, 8) | | | _ | | (4416, 100) | (100, 100) | | (100, 2) |
| T2D_4_13 | (1, 1, 64) | (3, 64, 32) | | | (1, 32, 512) | | | _ | | | | (3, 1, 32) | | | (3, 32, 512) | | | (1, 512, 512) | | | _ | | (5424, 100) | (100, 1) | | (1, 2) |
| T2D_4_14 | (1, 1, 128) | (3, 128, 8) | | | (3, 8, 128) | | | _ | | | | (3, 1, 8) | | | (3, 8, 32) | | | (3, 32, 128) | | | _ | | (4656, 100) | (100, 10) | | (10, 2) |
| T2D_4_6 | (3, 1, 32) | (3, 32, 512) | | | (1, 512, 8) | | | _ | | | | (1, 1, 8) | | | (1, 8, 128) | | | (1, 128, 8) | | | _ | | (4416, 100) | (100, 100) | | (100, 2) |
| T2D_4_7 | (3, 1, 32) | (3, 32, 512) | | | (3, 512, 512) | | | _ | | | | (3, 1, 8) | | | (3, 8, 8) | | | (3, 8, 512) | | | _ | | (5424, 10) | (10, 10) | | (10, 2) |
| T2D_4_8 | (1, 1, 64) | (3, 64, 512) | | | (1, 512, 128) | | | _ | | | | (1, 1, 32) | | | (3, 32, 512) | | | (1, 512, 128) | | | _ | | (4656, 100) | (100, 100) | | (100, 2) |
| T2D_4_9 | (3, 1, 128) | (3, 128, 512) | | | (3, 512, 512) | | | _ | | | | (3, 1, 512) | | | (1, 512, 32) | | | (3, 32, 512) | | | _ | | (1464, 10) | (10, 100) | | (100, 2) |
| T2D_5_10 | (3, 1, 64) | (1, 64, 64) | | | (1, 64, 512) | | | _ | | | | (3, 1, 32) | | | (3, 32, 32) | | | (1, 32, 512) | | | _ | | (5424, 100) | (100, 100) | | (100, 2) |
| T2D_5_11 | (3, 1, 128) | (3, 128, 512) | | | (3, 512, 128) | | | _ | | | | (1, 1, 512) | | | (1, 512, 8) | | | (3, 8, 128) | | | _ | | (696, 100) | (100, 10) | | (10, 2) |
| T2D_5_12 | (3, 1, 128) | (3, 128, 512) | | | (3, 512, 128) | | | _ | | | | (1, 1, 512) | | | (1, 512, 8) | | | (3, 8, 128) | | | _ | | (696, 100) | (100, 10) | | (10, 2) |
| T2D_5_13 | (3, 1, 128) | (3, 128, 512) | | | (3, 512, 128) | | | _ | | | | (1, 1, 512) | | | (1, 512, 8) | | | (3, 8, 128) | | | _ | | (696, 100) | (100, 10) | | (10, 2) |
| T2D_5_14 | (3, 1, 64) | (1, 64, 64) | | | (1, 64, 512) | | | _ | | | | (3, 1, 32) | | | (3, 32, 32) | | | (1, 32, 512) | | | _ | | (5424, 100) | (100, 100) | | (100, 2) |
| T2D_5_7 | (3, 1, 128) | (3, 128, 512) | | | (3, 512, 128) | | | _ | | | | (1, 1, 512) | | | (1, 512, 8) | | | (3, 8, 128) | | | _ | | (696, 100) | (100, 10) | | (10, 2) |
| T2D_5_8 | (3, 1, 128) | (3, 128, 64) | | | (3, 64, 512) | | | _ | | | | (3, 1, 8) | | | (1, 8, 512) | | | (3, 512, 512) | | | _ | | (1068, 100) | (100, 100) | | (100, 2) |
| T2D_5_9 | (3, 1, 128) | (3, 128, 64) | | | (3, 64, 512) | | | _ | | | | (3, 1, 8) | | | (1, 8, 512) | | | (3, 512, 512) | | | _ | | (1068, 100) | (100, 100) | | (100, 2) |
| T2D_6_10 | (3, 1, 128) | (3, 128, 512) | | | (3, 512, 128) | | | _ | | | | (1, 1, 512) | | | (1, 512, 8) | | | (3, 8, 128) | | | _ | | (696, 100) | (100, 10) | | (10, 2) |
| T2D_6_11 | (3, 1, 128) | (3, 128, 64) | | | (3, 64, 512) | | | _ | | | | (3, 1, 8) | | | (1, 8, 512) | | | (3, 512, 512) | | | _ | | (1068, 100) | (100, 100) | | (100, 2) |
| T2D_6_12 | (3, 1, 128) | (3, 128, 512) | | | (3, 512, 128) | | | _ | | | | (1, 1, 512) | | | (1, 512, 8) | | | (3, 8, 128) | | | _ | | (696, 100) | (100, 10) | | (10, 2) |
| T2D_6_13 | (3, 1, 128) | (3, 128, 512) | | | (3, 512, 128) | | | _ | | | | (1, 1, 512) | | | (1, 512, 8) | | | (3, 8, 128) | | | _ | | (696, 100) | (100, 10) | | (10, 2) |
| T2D_6_14 | (3, 1, 128) | (3, 128, 512) | | | (3, 512, 128) | | | _ | | | | (1, 1, 512) | | | (1, 512, 8) | | | (3, 8, 128) | | | _ | | (696, 100) | (100, 10) | | (10, 2) |
| T2D_6_8 | (3, 1, 128) | (3, 128, 512) | | | (3, 512, 128) | | | _ | | | | (1, 1, 512) | | | (1, 512, 8) | | | (3, 8, 128) | | | _ | | (696, 100) | (100, 10) | | (10, 2) |
| T2D_6_9 | (3, 1, 128) | (3, 128, 64) | | | (3, 64, 512) | | | _ | | | | (3, 1, 8) | | | (1, 8, 512) | | | (3, 512, 512) | | | _ | | (1068, 100) | (100, 100) | | (100, 2) |
| T2D_7_10 | (3, 1, 128) | (3, 128, 512) | | | (3, 512, 128) | | | _ | | | | (1, 1, 512) | | | (1, 512, 8) | | | (3, 8, 128) | | | _ | | (696, 100) | (100, 10) | | (10, 2) |
| T2D_7_11 | (3, 1, 128) | (3, 128, 512) | | | (3, 512, 512) | | | _ | | | | (3, 1, 512) | | | (1, 512, 32) | | | (3, 32, 512) | | | _ | | (1464, 10) | (10, 100) | | (100, 2) |
| T2D_7_12 | (3, 1, 128) | (3, 128, 512) | | | (3, 512, 128) | | | _ | | | | (1, 1, 512) | | | (1, 512, 8) | | | (3, 8, 128) | | | _ | | (696, 100) | (100, 10) | | (10, 2) |
| T2D_7_13 | (3, 1, 32) | (3, 32, 512) | | | (1, 512, 8) | | | _ | | | | (1, 1, 8) | | | (1, 8, 128) | | | (1, 128, 8) | | | _ | | (4416, 100) | (100, 100) | | (100, 2) |
| T2D_7_14 | (3, 1, 128) | (3, 128, 64) | | | (3, 64, 512) | | | _ | | | | (3, 1, 8) | | | (1, 8, 512) | | | (3, 512, 512) | | | _ | | (1068, 100) | (100, 100) | | (100, 2) |
| T2D_7_9 | (3, 1, 64) | (1, 64, 64) | | | (1, 64, 512) | | | _ | | | | (3, 1, 32) | | | (3, 32, 32) | | | (1, 32, 512) | | | _ | | (5424, 100) | (100, 100) | | (100, 2) |
| T2D_8_10 | (3, 1, 128) | (3, 128, 512) | | | (3, 512, 128) | | | _ | | | | (1, 1, 512) | | | (1, 512, 8) | | | (3, 8, 128) | | | _ | | (696, 100) | (100, 10) | | (10, 2) |
| T2D_8_11 | (3, 1, 32) | (3, 32, 512) | | | (1, 512, 8) | | | _ | | | | (1, 1, 8) | | | (1, 8, 128) | | | (1, 128, 8) | | | _ | | (4416, 100) | (100, 100) | | (100, 2) |
| T2D_8_12 | (1, 1, 8) | (3, 8, 64) | | | (3, 64, 8) | | | _ | | | | (3, 1, 32) | | | (3, 32, 8) | | | (3, 8, 8) | | | _ | | (4416, 100) | (100, 100) | | (100, 2) |
| T2D_8_13 | (3, 1, 128) | (3, 128, 512) | | | (3, 512, 128) | | | _ | | | | (1, 1, 512) | | | (1, 512, 8) | | | (3, 8, 128) | | | _ | | (696, 100) | (100, 10) | | (10, 2) |
| T2D_8_14 | (3, 1, 32) | (3, 32, 512) | | | (1, 512, 8) | | | _ | | | | (1, 1, 8) | | | (1, 8, 128) | | | (1, 128, 8) | | | _ | | (4416, 100) | (100, 100) | | (100, 2) |
| T2D_9_11 | (3, 1, 128) | (3, 128, 64) | | | (3, 64, 512) | | | _ | | | | (3, 1, 8) | | | (1, 8, 512) | | | (3, 512, 512) | | | _ | | (1068, 100) | (100, 100) | | (100, 2) |
| T2D_9_12 | (3, 1, 128) | (3, 128, 512) | | | (3, 512, 128) | | | _ | | | | (1, 1, 512) | | | (1, 512, 8) | | | (3, 8, 128) | | | _ | | (696, 100) | (100, 10) | | (10, 2) |
| T2D_9_13 | (3, 1, 64) | (1, 64, 64) | | | (1, 64, 512) | | | _ | | | | (3, 1, 32) | | | (3, 32, 32) | | | (1, 32, 512) | | | _ | | (5424, 100) | (100, 100) | | (100, 2) |
| T2D_9_14 | (3, 1, 128) | (3, 128, 64) | | | (3, 64, 512) | | | _ | | | | (3, 1, 8) | | | (1, 8, 512) | | | (3, 512, 512) | | | _ | | (1068, 100) | (100, 100) | | (100, 2) |
| Metabolic_10_12 | (3, 1, 128) | (3, 128, 64) | | | (3, 64, 512) | | | _ | | | | (3, 1, 8) | | | (1, 8, 512) | | | (3, 512, 512) | | | _ | | (1132, 100) | (100, 100) | | (100, 2) |
| Metabolic_10_13 | (3, 1, 128) | (3, 128, 64) | | | (3, 64, 512) | | | _ | | | | (3, 1, 8) | | | (1, 8, 512) | | | (3, 512, 512) | | | _ | | (1132, 100) | (100, 100) | | (100, 2) |
| Metabolic_11_13 | (3, 1, 8) | (1, 8, 64) | | | (1, 64, 64) | | | _ | | | | (1, 1, 8) | | | (3, 8, 32) | | | (1, 32, 64) | | | _ | | (236, 100) | (100, 100) | | (100, 2) |
| Metabolic_2_10 | (3, 1, 128) | (3, 128, 512) | | | (3, 512, 512) | | | _ | | | | (3, 1, 512) | | | (1, 512, 32) | | | (3, 32, 512) | | | _ | | (2104, 10) | (10, 100) | | (100, 2) |
| Metabolic_2_11 | (3, 1, 128) | (3, 128, 64) | | | (3, 64, 512) | | | _ | | | | (3, 1, 8) | | | (1, 8, 512) | | | (3, 512, 512) | | | _ | | (1132, 100) | (100, 100) | | (100, 2) |
| Metabolic_2_12 | (3, 1, 128) | (3, 128, 512) | | | (3, 512, 128) | | | _ | | | | (1, 1, 512) | | | (1, 512, 8) | | | (3, 8, 128) | | | _ | | (1336, 100) | (100, 10) | | (10, 2) |
| Metabolic_2_13 | (3, 1, 128) | (3, 128, 512) | | | (3, 512, 128) | | | _ | | | | (1, 1, 512) | | | (1, 512, 8) | | | (3, 8, 128) | | | _ | | (1336, 100) | (100, 10) | | (10, 2) |
| Metabolic_2_4 | (1, 1, 8) | (1, 8, 512) | | | (1, 512, 512) | | | _ | | | | (3, 1, 512) | | | (3, 512, 64) | | | (1, 64, 512) | | | _ | | (1132, 100) | (100, 100) | | (100, 2) |
| Metabolic_2_5 | (3, 1, 128) | (3, 128, 64) | | | (3, 64, 512) | | | _ | | | | (3, 1, 8) | | | (1, 8, 512) | | | (3, 512, 512) | | | _ | | (1132, 100) | (100, 100) | | (100, 2) |
| Metabolic_2_6 | (3, 1, 128) | (3, 128, 8) | | | (3, 8, 32) | | | _ | | | | (1, 1, 128) | | | (3, 128, 512) | | | (3, 512, 32) | | | _ | | (1144, 100) | (100, 100) | | (100, 2) |
| Metabolic_2_7 | (3, 1, 128) | (3, 128, 64) | | | (3, 64, 512) | | | _ | | | | (3, 1, 8) | | | (1, 8, 512) | | | (3, 512, 512) | | | _ | | (1132, 100) | (100, 100) | | (100, 2) |
| Metabolic_2_8 | (1, 1, 8) | (1, 8, 512) | | | (1, 512, 512) | | | _ | | | | (3, 1, 128) | | | (1, 128, 64) | | | (1, 64, 512) | | | _ | | (2104, 100) | (100, 100) | | (100, 2) |
| Metabolic_2_9 | (3, 1, 128) | (3, 128, 512) | | | (3, 512, 512) | | | _ | | | | (3, 1, 512) | | | (1, 512, 32) | | | (3, 32, 512) | | | _ | | (2104, 10) | (10, 100) | | (100, 2) |
| Metabolic_3_10 | (1, 1, 512) | (3, 512, 32) | | | (1, 32, 512) | | | _ | | | | (1, 1, 64) | | | (1, 64, 64) | | | (1, 64, 512) | | | _ | | (2104, 100) | (100, 10) | | (10, 2) |
| Metabolic_3_11 | (3, 1, 128) | (3, 128, 512) | | | (3, 512, 512) | | | _ | | | | (3, 1, 512) | | | (1, 512, 32) | | | (3, 32, 512) | | | _ | | (2104, 10) | (10, 100) | | (100, 2) |
| Metabolic_3_12 | (3, 1, 32) | (3, 32, 512) | | | (3, 512, 512) | | | _ | | | | (3, 1, 8) | | | (3, 8, 8) | | | (3, 8, 512) | | | _ | | (11824, 10) | (10, 10) | | (10, 2) |
| Metabolic_3_13 | (3, 1, 128) | (3, 128, 64) | | | (3, 64, 512) | | | _ | | | | (3, 1, 8) | | | (1, 8, 512) | | | (3, 512, 512) | | | _ | | (1132, 100) | (100, 100) | | (100, 2) |
| Metabolic_3_5 | (3, 1, 128) | (3, 128, 512) | | | (3, 512, 512) | | | _ | | | | (3, 1, 512) | | | (1, 512, 32) | | | (3, 32, 512) | | | _ | | (2104, 10) | (10, 100) | | (100, 2) |
| Metabolic_3_6 | (3, 1, 128) | (3, 128, 512) | | | (3, 512, 512) | | | _ | | | | (3, 1, 512) | | | (1, 512, 32) | | | (3, 32, 512) | | | _ | | (2104, 10) | (10, 100) | | (100, 2) |
| Metabolic_3_7 | (3, 1, 128) | (3, 128, 64) | | | (3, 64, 512) | | | _ | | | | (3, 1, 8) | | | (1, 8, 512) | | | (3, 512, 512) | | | _ | | (1132, 100) | (100, 100) | | (100, 2) |
| Metabolic_3_8 | (3, 1, 128) | (3, 128, 512) | | | (3, 512, 512) | | | _ | | | | (3, 1, 512) | | | (1, 512, 32) | | | (3, 32, 512) | | | _ | | (2104, 10) | (10, 100) | | (100, 2) |
| Metabolic_3_9 | (1, 1, 128) | (3, 128, 8) | | | (1, 8, 64) | | | _ | | | | (3, 1, 64) | | | (1, 64, 64) | | | (1, 64, 64) | | | _ | | (1208, 100) | (100, 100) | | (100, 2) |
| Metabolic_4_10 | (3, 1, 128) | (3, 128, 8) | | | (3, 8, 32) | | | _ | | | | (1, 1, 128) | | | (3, 128, 512) | | | (3, 512, 32) | | | _ | | (1144, 100) | (100, 100) | | (100, 2) |
| Metabolic_4_11 | (3, 1, 32) | (1, 32, 512) | | | (3, 512, 32) | | | _ | | | | (1, 1, 32) | | | (3, 32, 512) | | | (3, 512, 32) | | | _ | | (1144, 10) | (10, 100) | | (100, 2) |
| Metabolic_4_12 | (1, 1, 8) | (1, 8, 512) | | | (1, 512, 512) | | | _ | | | | (3, 1, 128) | | | (1, 128, 64) | | | (1, 64, 512) | | | _ | | (2104, 100) | (100, 100) | | (100, 2) |
| Metabolic_4_13 | (1, 1, 64) | (3, 64, 512) | | | (1, 512, 128) | | | _ | | | | (1, 1, 32) | | | (3, 32, 512) | | | (1, 512, 128) | | | _ | | (11056, 100) | (100, 100) | | (100, 2) |
| Metabolic_4_6 | (3, 1, 128) | (3, 128, 64) | | | (3, 64, 512) | | | _ | | | | (3, 1, 8) | | | (1, 8, 512) | | | (3, 512, 512) | | | _ | | (1132, 100) | (100, 100) | | (100, 2) |
| Metabolic_4_7 | (3, 1, 128) | (3, 128, 64) | | | (3, 64, 512) | | | _ | | | | (3, 1, 8) | | | (1, 8, 512) | | | (3, 512, 512) | | | _ | | (1132, 100) | (100, 100) | | (100, 2) |
| Metabolic_4_8 | (3, 1, 128) | (3, 128, 512) | | | (3, 512, 512) | | | _ | | | | (3, 1, 512) | | | (1, 512, 32) | | | (3, 32, 512) | | | _ | | (2104, 10) | (10, 100) | | (100, 2) |
| Metabolic_4_9 | (3, 1, 128) | (3, 128, 64) | | | (3, 64, 512) | | | _ | | | | (3, 1, 8) | | | (1, 8, 512) | | | (3, 512, 512) | | | _ | | (1132, 100) | (100, 100) | | (100, 2) |
| Metabolic_5_10 | (3, 1, 128) | (3, 128, 64) | | | (3, 64, 512) | | | _ | | | | (3, 1, 8) | | | (1, 8, 512) | | | (3, 512, 512) | | | _ | | (1132, 100) | (100, 100) | | (100, 2) |
| Metabolic_5_11 | (1, 1, 8) | (3, 8, 64) | | | (3, 64, 512) | | | _ | | | | (1, 1, 128) | | | (1, 128, 32) | | | (3, 32, 512) | | | _ | | (2104, 10) | (10, 10) | | (10, 2) |
| Metabolic_5_12 | (3, 1, 128) | (3, 128, 64) | | | (3, 64, 512) | | | _ | | | | (3, 1, 8) | | | (1, 8, 512) | | | (3, 512, 512) | | | _ | | (1132, 100) | (100, 100) | | (100, 2) |
| Metabolic_5_13 | (3, 1, 32) | (3, 32, 512) | | | (3, 512, 512) | | | _ | | | | (3, 1, 8) | | | (3, 8, 8) | | | (3, 8, 512) | | | _ | | (11824, 10) | (10, 10) | | (10, 2) |
| Metabolic_5_7 | (3, 1, 128) | (3, 128, 8) | | | (3, 8, 32) | | | _ | | | | (1, 1, 128) | | | (3, 128, 512) | | | (3, 512, 32) | | | _ | | (1144, 100) | (100, 100) | | (100, 2) |
| Metabolic_5_8 | (3, 1, 128) | (3, 128, 512) | | | (3, 512, 512) | | | _ | | | | (3, 1, 512) | | | (1, 512, 32) | | | (3, 32, 512) | | | _ | | (2104, 10) | (10, 100) | | (100, 2) |
| Metabolic_5_9 | (3, 1, 128) | (3, 128, 8) | | | (3, 8, 32) | | | _ | | | | (1, 1, 128) | | | (3, 128, 512) | | | (3, 512, 32) | | | _ | | (1144, 100) | (100, 100) | | (100, 2) |
| Metabolic_6_10 | (3, 1, 128) | (3, 128, 512) | | | (3, 512, 512) | | | _ | | | | (3, 1, 512) | | | (1, 512, 32) | | | (3, 32, 512) | | | _ | | (2104, 10) | (10, 100) | | (100, 2) |
| Metabolic_6_11 | (3, 1, 128) | (3, 128, 512) | | | (3, 512, 512) | | | _ | | | | (3, 1, 512) | | | (1, 512, 32) | | | (3, 32, 512) | | | _ | | (2104, 10) | (10, 100) | | (100, 2) |
| Metabolic_6_12 | (3, 1, 128) | (3, 128, 8) | | | (3, 8, 32) | | | _ | | | | (1, 1, 128) | | | (3, 128, 512) | | | (3, 512, 32) | | | _ | | (1144, 100) | (100, 100) | | (100, 2) |
| Metabolic_6_13 | (3, 1, 128) | (3, 128, 8) | | | (3, 8, 32) | | | _ | | | | (1, 1, 128) | | | (3, 128, 512) | | | (3, 512, 32) | | | _ | | (1144, 100) | (100, 100) | | (100, 2) |
| Metabolic_6_8 | (3, 1, 128) | (3, 128, 64) | | | (3, 64, 512) | | | _ | | | | (3, 1, 8) | | | (1, 8, 512) | | | (3, 512, 512) | | | _ | | (1132, 100) | (100, 100) | | (100, 2) |
| Metabolic_6_9 | (3, 1, 128) | (3, 128, 8) | | | (3, 8, 32) | | | _ | | | | (1, 1, 128) | | | (3, 128, 512) | | | (3, 512, 32) | | | _ | | (1144, 100) | (100, 100) | | (100, 2) |
| Metabolic_7_10 | (3, 1, 128) | (3, 128, 64) | | | (3, 64, 512) | | | _ | | | | (3, 1, 8) | | | (1, 8, 512) | | | (3, 512, 512) | | | _ | | (1132, 100) | (100, 100) | | (100, 2) |
| Metabolic_7_11 | (3, 1, 128) | (3, 128, 64) | | | (3, 64, 512) | | | _ | | | | (3, 1, 8) | | | (1, 8, 512) | | | (3, 512, 512) | | | _ | | (1132, 100) | (100, 100) | | (100, 2) |
| Metabolic_7_12 | (3, 1, 128) | (3, 128, 8) | | | (3, 8, 32) | | | _ | | | | (1, 1, 128) | | | (3, 128, 512) | | | (3, 512, 32) | | | _ | | (1144, 100) | (100, 100) | | (100, 2) |
| Metabolic_7_13 | (1, 1, 8) | (1, 8, 512) | | | (1, 512, 512) | | | _ | | | | (3, 1, 128) | | | (1, 128, 64) | | | (1, 64, 512) | | | _ | | (2104, 100) | (100, 100) | | (100, 2) |
| Metabolic_7_9 | (3, 1, 128) | (3, 128, 64) | | | (3, 64, 512) | | | _ | | | | (3, 1, 8) | | | (1, 8, 512) | | | (3, 512, 512) | | | _ | | (1132, 100) | (100, 100) | | (100, 2) |
| Metabolic_8_10 | (3, 1, 128) | (3, 128, 64) | | | (3, 64, 512) | | | _ | | | | (3, 1, 8) | | | (1, 8, 512) | | | (3, 512, 512) | | | _ | | (1132, 100) | (100, 100) | | (100, 2) |
| Metabolic_8_11 | (3, 1, 128) | (3, 128, 64) | | | (3, 64, 512) | | | _ | | | | (3, 1, 8) | | | (1, 8, 512) | | | (3, 512, 512) | | | _ | | (1132, 100) | (100, 100) | | (100, 2) |
| Metabolic_8_12 | (3, 1, 128) | (3, 128, 512) | | | (3, 512, 128) | | | _ | | | | (1, 1, 512) | | | (1, 512, 8) | | | (3, 8, 128) | | | _ | | (1336, 100) | (100, 10) | | (10, 2) |
| Metabolic_8_13 | (3, 1, 128) | (3, 128, 64) | | | (3, 64, 512) | | | _ | | | | (3, 1, 8) | | | (1, 8, 512) | | | (3, 512, 512) | | | _ | | (1132, 100) | (100, 100) | | (100, 2) |
| Metabolic_9_11 | (3, 1, 128) | (3, 128, 512) | | | (3, 512, 128) | | | _ | | | | (1, 1, 512) | | | (1, 512, 8) | | | (3, 8, 128) | | | _ | | (1336, 100) | (100, 10) | | (10, 2) |
| Metabolic_9_12 | (3, 1, 128) | (3, 128, 64) | | | (3, 64, 512) | | | _ | | | | (3, 1, 8) | | | (1, 8, 512) | | | (3, 512, 512) | | | _ | | (1132, 100) | (100, 100) | | (100, 2) |
| Metabolic_9_13 | (3, 1, 128) | (3, 128, 8) | | | (3, 8, 32) | | | _ | | | | (1, 1, 128) | | | (3, 128, 512) | | | (3, 512, 32) | | | _ | | (1144, 100) | (100, 100) | | (100, 2) |
| Prediabetes_10_12 | (3, 1, 128) | (3, 128, 512) | | | (3, 512, 128) | | | _ | | | | (1, 1, 512) | | | (1, 512, 8) | | | (3, 8, 128) | | | _ | | (2826, 100) | (100, 10) | | (10, 2) |
| Prediabetes_10_13 | (3, 1, 128) | (3, 128, 64) | | | (3, 64, 512) | | | _ | | | | (3, 1, 8) | | | (1, 8, 512) | | | (3, 512, 512) | | | _ | | (1281, 100) | (100, 100) | | (100, 2) |
| Prediabetes_11_13 | (3, 1, 128) | (3, 128, 64) | | | (3, 64, 512) | | | _ | | | | (3, 1, 8) | | | (1, 8, 512) | | | (3, 512, 512) | | | _ | | (1281, 100) | (100, 100) | | (100, 2) |
| Prediabetes_2_10 | (3, 1, 32) | (3, 32, 32) | | | (1, 32, 512) | | | _ | | | | (1, 1, 8) | | | (3, 8, 128) | | | (1, 128, 512) | | | _ | | (26724, 100) | (100, 100) | | (100, 2) |
| Prediabetes_2_11 | (3, 1, 128) | (3, 128, 64) | | | (3, 64, 512) | | | _ | | | | (3, 1, 8) | | | (1, 8, 512) | | | (3, 512, 512) | | | _ | | (1281, 100) | (100, 100) | | (100, 2) |
| Prediabetes_2_12 | (3, 1, 128) | (3, 128, 512) | | | (3, 512, 128) | | | _ | | | | (1, 1, 512) | | | (1, 512, 8) | | | (3, 8, 128) | | | _ | | (2826, 100) | (100, 10) | | (10, 2) |
| Prediabetes_2_13 | (1, 1, 8) | (3, 8, 64) | | | (3, 64, 8) | | | _ | | | | (3, 1, 32) | | | (3, 32, 8) | | | (3, 8, 8) | | | _ | | (25716, 100) | (100, 100) | | (100, 2) |
| Prediabetes_2_4 | (3, 1, 128) | (3, 128, 512) | | | (3, 512, 128) | | | _ | | | | (1, 1, 512) | | | (1, 512, 8) | | | (3, 8, 128) | | | _ | | (2826, 100) | (100, 10) | | (10, 2) |
| Prediabetes_2_5 | (1, 1, 128) | (3, 128, 8) | | | (1, 8, 64) | | | _ | | | | (3, 1, 64) | | | (1, 64, 64) | | | (1, 64, 64) | | | _ | | (2698, 100) | (100, 100) | | (100, 2) |
| Prediabetes_2_6 | (3, 1, 128) | (3, 128, 64) | | | (3, 64, 512) | | | _ | | | | (3, 1, 8) | | | (1, 8, 512) | | | (3, 512, 512) | | | _ | | (1281, 100) | (100, 100) | | (100, 2) |
| Prediabetes_2_7 | (3, 1, 128) | (3, 128, 64) | | | (3, 64, 512) | | | _ | | | | (3, 1, 8) | | | (1, 8, 512) | | | (3, 512, 512) | | | _ | | (1281, 100) | (100, 100) | | (100, 2) |
| Prediabetes_2_8 | (1, 1, 8) | (3, 8, 64) | | | (3, 64, 8) | | | _ | | | | (3, 1, 32) | | | (3, 32, 8) | | | (3, 8, 8) | | | _ | | (25716, 100) | (100, 100) | | (100, 2) |
| Prediabetes_2_9 | (1, 1, 8) | (3, 8, 64) | | | (3, 64, 8) | | | _ | | | | (3, 1, 32) | | | (3, 32, 8) | | | (3, 8, 8) | | | _ | | (25716, 100) | (100, 100) | | (100, 2) |
| Prediabetes_3_10 | (3, 1, 128) | (3, 128, 64) | | | (3, 64, 512) | | | _ | | | | (3, 1, 8) | | | (1, 8, 512) | | | (3, 512, 512) | | | _ | | (1281, 100) | (100, 100) | | (100, 2) |
| Prediabetes_3_11 | (3, 1, 128) | (3, 128, 64) | | | (3, 64, 512) | | | _ | | | | (3, 1, 8) | | | (1, 8, 512) | | | (3, 512, 512) | | | _ | | (1281, 100) | (100, 100) | | (100, 2) |
| Prediabetes_3_12 | (3, 1, 128) | (3, 128, 512) | | | (3, 512, 512) | | | _ | | | | (3, 1, 512) | | | (1, 512, 32) | | | (3, 32, 512) | | | _ | | (3594, 10) | (10, 100) | | (100, 2) |
| Prediabetes_3_13 | (3, 1, 128) | (3, 128, 64) | | | (3, 64, 512) | | | _ | | | | (3, 1, 8) | | | (1, 8, 512) | | | (3, 512, 512) | | | _ | | (1281, 100) | (100, 100) | | (100, 2) |
| Prediabetes_3_5 | (3, 1, 64) | (3, 64, 512) | | | (3, 512, 64) | | | _ | | | | (3, 1, 512) | | | (3, 512, 512) | | | (3, 512, 64) | | | _ | | (2698, 10) | (10, 10) | | (10, 2) |
| Prediabetes_3_6 | (1, 1, 64) | (3, 64, 512) | | | (1, 512, 128) | | | _ | | | | (1, 1, 32) | | | (3, 32, 512) | | | (1, 512, 128) | | | _ | | (25956, 100) | (100, 100) | | (100, 2) |
| Prediabetes_3_7 | (3, 1, 32) | (3, 32, 512) | | | (3, 512, 64) | | | _ | | | | (1, 1, 32) | | | (1, 32, 512) | | | (3, 512, 64) | | | _ | | (25828, 10) | (10, 10) | | (10, 2) |
| Prediabetes_3_8 | (3, 1, 128) | (3, 128, 64) | | | (3, 64, 512) | | | _ | | | | (3, 1, 8) | | | (1, 8, 512) | | | (3, 512, 512) | | | _ | | (1281, 100) | (100, 100) | | (100, 2) |
| Prediabetes_3_9 | (3, 1, 128) | (3, 128, 64) | | | (3, 64, 512) | | | _ | | | | (3, 1, 8) | | | (1, 8, 512) | | | (3, 512, 512) | | | _ | | (1281, 100) | (100, 100) | | (100, 2) |
| Prediabetes_4_10 | (3, 1, 32) | (3, 32, 512) | | | (3, 512, 64) | | | _ | | | | (1, 1, 32) | | | (1, 32, 512) | | | (3, 512, 64) | | | _ | | (25828, 10) | (10, 10) | | (10, 2) |
| Prediabetes_4_11 | (3, 1, 128) | (3, 128, 64) | | | (3, 64, 512) | | | _ | | | | (3, 1, 8) | | | (1, 8, 512) | | | (3, 512, 512) | | | _ | | (1281, 100) | (100, 100) | | (100, 2) |
| Prediabetes_4_12 | (3, 1, 128) | (3, 128, 64) | | | (3, 64, 512) | | | _ | | | | (3, 1, 8) | | | (1, 8, 512) | | | (3, 512, 512) | | | _ | | (1281, 100) | (100, 100) | | (100, 2) |
| Prediabetes_4_13 | (3, 1, 32) | (3, 32, 32) | | | (1, 32, 512) | | | _ | | | | (1, 1, 8) | | | (3, 8, 128) | | | (1, 128, 512) | | | _ | | (26724, 100) | (100, 100) | | (100, 2) |
| Prediabetes_4_6 | (3, 1, 128) | (3, 128, 512) | | | (3, 512, 128) | | | _ | | | | (1, 1, 512) | | | (1, 512, 8) | | | (3, 8, 128) | | | _ | | (2826, 100) | (100, 10) | | (10, 2) |
| Prediabetes_4_7 | (3, 1, 128) | (3, 128, 512) | | | (3, 512, 128) | | | _ | | | | (1, 1, 512) | | | (1, 512, 8) | | | (3, 8, 128) | | | _ | | (2826, 100) | (100, 10) | | (10, 2) |
| Prediabetes_4_8 | (3, 1, 128) | (3, 128, 64) | | | (3, 64, 512) | | | _ | | | | (3, 1, 8) | | | (1, 8, 512) | | | (3, 512, 512) | | | _ | | (1281, 100) | (100, 100) | | (100, 2) |
| Prediabetes_4_9 | (3, 1, 128) | (3, 128, 64) | | | (3, 64, 512) | | | _ | | | | (3, 1, 8) | | | (1, 8, 512) | | | (3, 512, 512) | | | _ | | (1281, 100) | (100, 100) | | (100, 2) |
| Prediabetes_5_10 | (3, 1, 128) | (3, 128, 64) | | | (3, 64, 512) | | | _ | | | | (3, 1, 8) | | | (1, 8, 512) | | | (3, 512, 512) | | | _ | | (1281, 100) | (100, 100) | | (100, 2) |
| Prediabetes_5_11 | (3, 1, 128) | (3, 128, 512) | | | (3, 512, 128) | | | _ | | | | (1, 1, 512) | | | (1, 512, 8) | | | (3, 8, 128) | | | _ | | (2826, 100) | (100, 10) | | (10, 2) |
| Prediabetes_5_12 | (1, 1, 128) | (3, 128, 8) | | | (1, 8, 64) | | | _ | | | | (3, 1, 64) | | | (1, 64, 64) | | | (1, 64, 64) | | | _ | | (2698, 100) | (100, 100) | | (100, 2) |
| Prediabetes_5_13 | (3, 1, 128) | (3, 128, 64) | | | (3, 64, 512) | | | _ | | | | (3, 1, 8) | | | (1, 8, 512) | | | (3, 512, 512) | | | _ | | (1281, 100) | (100, 100) | | (100, 2) |
| Prediabetes_5_7 | (3, 1, 128) | (3, 128, 512) | | | (3, 512, 512) | | | _ | | | | (3, 1, 512) | | | (1, 512, 32) | | | (3, 32, 512) | | | _ | | (3594, 10) | (10, 100) | | (100, 2) |
| Prediabetes_5_8 | (3, 1, 128) | (3, 128, 64) | | | (1, 64, 8) | | | _ | | | | (1, 1, 64) | | | (3, 64, 128) | | | (1, 128, 8) | | | _ | | (2586, 100) | (100, 10) | | (10, 2) |
| Prediabetes_5_9 | (3, 1, 128) | (3, 128, 8) | | | (3, 8, 32) | | | _ | | | | (1, 1, 128) | | | (3, 128, 512) | | | (3, 512, 32) | | | _ | | (2634, 100) | (100, 100) | | (100, 2) |
| Prediabetes_6_10 | (3, 1, 8) | (1, 8, 64) | | | (1, 64, 64) | | | _ | | | | (1, 1, 8) | | | (3, 8, 32) | | | (1, 32, 64) | | | _ | | (385, 100) | (100, 100) | | (100, 2) |
| Prediabetes_6_11 | (1, 1, 8) | (1, 8, 512) | | | (1, 512, 64) | | | _ | | | | (3, 1, 512) | | | (3, 512, 128) | | | (1, 128, 64) | | | _ | | (2698, 10) | (10, 100) | | (100, 2) |
| Prediabetes_6_12 | (3, 1, 128) | (3, 128, 512) | | | (3, 512, 128) | | | _ | | | | (1, 1, 512) | | | (1, 512, 8) | | | (3, 8, 128) | | | _ | | (2826, 100) | (100, 10) | | (10, 2) |
| Prediabetes_6_13 | (3, 1, 128) | (3, 128, 512) | | | (3, 512, 512) | | | _ | | | | (3, 1, 512) | | | (1, 512, 32) | | | (3, 32, 512) | | | _ | | (3594, 10) | (10, 100) | | (100, 2) |
| Prediabetes_6_8 | (3, 1, 128) | (3, 128, 64) | | | (3, 64, 512) | | | _ | | | | (3, 1, 8) | | | (1, 8, 512) | | | (3, 512, 512) | | | _ | | (1281, 100) | (100, 100) | | (100, 2) |
| Prediabetes_6_9 | (3, 1, 128) | (3, 128, 64) | | | (3, 64, 512) | | | _ | | | | (3, 1, 8) | | | (1, 8, 512) | | | (3, 512, 512) | | | _ | | (1281, 100) | (100, 100) | | (100, 2) |
| Prediabetes_7_10 | (3, 1, 128) | (3, 128, 512) | | | (3, 512, 512) | | | _ | | | | (3, 1, 512) | | | (1, 512, 32) | | | (3, 32, 512) | | | _ | | (3594, 10) | (10, 100) | | (100, 2) |
| Prediabetes_7_11 | (3, 1, 128) | (3, 128, 512) | | | (3, 512, 512) | | | _ | | | | (3, 1, 512) | | | (1, 512, 32) | | | (3, 32, 512) | | | _ | | (3594, 10) | (10, 100) | | (100, 2) |
| Prediabetes_7_12 | (3, 1, 64) | (3, 64, 8) | | | (3, 8, 64) | | | _ | | | | (3, 1, 64) | | | (3, 64, 64) | | | (3, 64, 64) | | | _ | | (385, 100) | (100, 10) | | (10, 2) |
| Prediabetes_7_13 | (3, 1, 128) | (3, 128, 8) | | | (3, 8, 32) | | | _ | | | | (1, 1, 128) | | | (3, 128, 512) | | | (3, 512, 32) | | | _ | | (2634, 100) | (100, 100) | | (100, 2) |
| Prediabetes_7_9 | (3, 1, 64) | (3, 64, 512) | | | (3, 512, 64) | | | _ | | | | (3, 1, 512) | | | (3, 512, 512) | | | (3, 512, 64) | | | _ | | (2698, 10) | (10, 10) | | (10, 2) |
| Prediabetes_8_10 | (1, 1, 8) | (1, 8, 512) | | | (1, 512, 512) | | | _ | | | | (3, 1, 128) | | | (1, 128, 64) | | | (1, 64, 512) | | | _ | | (3594, 100) | (100, 100) | | (100, 2) |
| Prediabetes_8_11 | (3, 1, 128) | (3, 128, 64) | | | (1, 64, 8) | | | _ | | | | (1, 1, 64) | | | (3, 64, 128) | | | (1, 128, 8) | | | _ | | (2586, 100) | (100, 10) | | (10, 2) |
| Prediabetes_8_12 | (1, 1, 128) | (3, 128, 128) | | | (3, 128, 64) | | | _ | | | | (3, 1, 32) | | | (1, 32, 512) | | | (3, 512, 64) | | | _ | | (25828, 10) | (10, 10) | | (10, 2) |
| Prediabetes_8_13 | (3, 1, 128) | (3, 128, 64) | | | (3, 64, 512) | | | _ | | | | (3, 1, 8) | | | (1, 8, 512) | | | (3, 512, 512) | | | _ | | (1281, 100) | (100, 100) | | (100, 2) |
| Prediabetes_9_11 | (3, 1, 128) | (3, 128, 64) | | | (1, 64, 8) | | | _ | | | | (1, 1, 64) | | | (3, 64, 128) | | | (1, 128, 8) | | | _ | | (2586, 100) | (100, 10) | | (10, 2) |
| Prediabetes_9_12 | (3, 1, 128) | (3, 128, 8) | | | (3, 8, 32) | | | _ | | | | (1, 1, 128) | | | (3, 128, 512) | | | (3, 512, 32) | | | _ | | (2634, 100) | (100, 100) | | (100, 2) |
| Prediabetes_9_13 | (3, 1, 128) | (3, 128, 512) | | | (3, 512, 512) | | | _ | | | | (3, 1, 512) | | | (1, 512, 32) | | | (3, 32, 512) | | | _ | | (3594, 10) | (10, 100) | | (100, 2) |

The embedding size of each Conv 1D consists of kernel width, input size, and number of convolution kernels. Embedding size of each Dense layer includes input size an output size.

**Supplementary Table 14.** Embedding size of Transformers

| Cohort Name | Encoder B1 | | | | | | Encoder B2 | | | | | | Encoder B3 | | | | | | Encoder B4 | | | | | Concatenation | | |
| --- | --- | --- | --- | --- | --- | --- | --- | --- | --- | --- | --- | --- | --- | --- | --- | --- | --- | --- | --- | --- | --- | --- | --- | --- | --- | --- |
|  | MHA | Conv 1D | | | Conv 1D | | MHA | | Conv 1D | | Conv 1D | | MHA | | Conv 1D | | Conv 1D | | MHA | | Conv 1D | Conv 1D | | Dense | Dense | |
| T2D_10_12 | (1, 4, 2) | | (1, 1, 4) | (1, 4, 1) | | (1, 1, 1) | | (1, 1, 1) | | (1, 1, 1) | | (1, 2, 8) | | (1, 1, 1) | | (1, 1, 1) | | (1, 1, 8) | | (1, 1, 8) | | (1, 8, 1) | (48, 128) | | | (128, 2) |
| T2D_10_13 | (1, 4, 2) | | (1, 1, 4) | (1, 4, 1) | | (1, 1, 1) | | (1, 1, 1) | | (1, 1, 1) | | (1, 2, 8) | | (1, 1, 1) | | (1, 1, 1) | | (1, 1, 8) | | (1, 1, 8) | | (1, 8, 1) | (50, 128) | | | (128, 2) |
| T2D_10_14 | (1, 4, 2) | | (1, 1, 4) | (1, 4, 1) | | (1, 1, 1) | | (1, 1, 1) | | (1, 1, 1) | | (1, 2, 8) | | (1, 1, 1) | | (1, 1, 1) | | (1, 1, 8) | | (1, 1, 8) | | (1, 8, 1) | (52, 128) | | | (128, 2) |
| T2D_11_13 | (1, 4, 2) | | (1, 1, 4) | (1, 4, 1) | | (1, 1, 1) | | (1, 1, 1) | | (1, 1, 1) | | (1, 2, 8) | | (1, 1, 1) | | (1, 1, 1) | | (1, 1, 8) | | (1, 1, 8) | | (1, 8, 1) | (48, 128) | | | (128, 2) |
| T2D_11_14 | (1, 4, 2) | | (1, 1, 4) | (1, 4, 1) | | (1, 1, 1) | | (1, 1, 1) | | (1, 1, 1) | | (1, 2, 8) | | (1, 1, 1) | | (1, 1, 1) | | (1, 1, 8) | | (1, 1, 8) | | (1, 8, 1) | (50, 128) | | | (128, 2) |
| T2D_12_14 | (1, 4, 2) | | (1, 1, 4) | (1, 4, 1) | | (1, 1, 1) | | (1, 1, 1) | | (1, 1, 1) | | (1, 2, 8) | | (1, 1, 1) | | (1, 1, 1) | | (1, 1, 8) | | (1, 1, 8) | | (1, 8, 1) | (48, 128) | | | (128, 2) |
| T2D_3_10 | (1, 4, 2) | | (1, 1, 4) | (1, 4, 1) | | (1, 1, 1) | | (1, 1, 1) | | (1, 1, 1) | | (1, 2, 8) | | (1, 1, 1) | | (1, 1, 1) | | (1, 1, 8) | | (1, 1, 8) | | (1, 8, 1) | (58, 128) | | | (128, 2) |
| T2D_3_11 | (1, 4, 2) | | (1, 1, 4) | (1, 4, 1) | | (1, 1, 1) | | (1, 1, 1) | | (1, 1, 1) | | (1, 2, 8) | | (1, 1, 1) | | (1, 1, 1) | | (1, 1, 8) | | (1, 1, 8) | | (1, 8, 1) | (60, 128) | | | (128, 2) |
| T2D_3_12 | (1, 4, 2) | | (1, 1, 4) | (1, 4, 1) | | (1, 1, 1) | | (1, 1, 1) | | (1, 1, 1) | | (1, 2, 8) | | (1, 1, 1) | | (1, 1, 1) | | (1, 1, 8) | | (1, 1, 8) | | (1, 8, 1) | (62, 128) | | | (128, 2) |
| T2D_3_13 | (1, 4, 2) | | (1, 1, 4) | (1, 4, 1) | | (1, 1, 1) | | (1, 1, 1) | | (1, 1, 1) | | (1, 2, 8) | | (1, 1, 1) | | (1, 1, 1) | | (1, 1, 8) | | (1, 1, 8) | | (1, 8, 1) | (64, 128) | | | (128, 2) |
| T2D_3_14 | (1, 4, 2) | | (1, 1, 4) | (1, 4, 1) | | (1, 1, 1) | | (1, 1, 1) | | (1, 1, 1) | | (1, 2, 8) | | (1, 1, 1) | | (1, 1, 1) | | (1, 1, 8) | | (1, 1, 8) | | (1, 8, 1) | (66, 128) | | | (128, 2) |
| T2D_3_5 | (1, 4, 2) | | (1, 1, 4) | (1, 4, 1) | | (1, 1, 1) | | (1, 1, 1) | | (1, 1, 1) | | (1, 2, 8) | | (1, 1, 1) | | (1, 1, 1) | | (1, 1, 8) | | (1, 1, 8) | | (1, 8, 1) | (48, 128) | | | (128, 2) |
| T2D_3_6 | (1, 4, 2) | | (1, 1, 4) | (1, 4, 1) | | (1, 1, 1) | | (1, 1, 1) | | (1, 1, 1) | | (1, 2, 8) | | (1, 1, 1) | | (1, 1, 1) | | (1, 1, 8) | | (1, 1, 8) | | (1, 8, 1) | (50, 128) | | | (128, 2) |
| T2D_3_7 | (1, 4, 2) | | (1, 1, 4) | (1, 4, 1) | | (1, 1, 1) | | (1, 1, 1) | | (1, 1, 1) | | (1, 2, 8) | | (1, 1, 1) | | (1, 1, 1) | | (1, 1, 8) | | (1, 1, 8) | | (1, 8, 1) | (52, 128) | | | (128, 2) |
| T2D_3_8 | (1, 4, 2) | | (1, 1, 4) | (1, 4, 1) | | (1, 1, 1) | | (1, 1, 1) | | (1, 1, 1) | | (1, 2, 8) | | (1, 1, 1) | | (1, 1, 1) | | (1, 1, 8) | | (1, 1, 8) | | (1, 8, 1) | (54, 128) | | | (128, 2) |
| T2D_3_9 | (1, 4, 2) | | (1, 1, 4) | (1, 4, 1) | | (1, 1, 1) | | (1, 1, 1) | | (1, 1, 1) | | (1, 2, 8) | | (1, 1, 1) | | (1, 1, 1) | | (1, 1, 8) | | (1, 1, 8) | | (1, 8, 1) | (56, 128) | | | (128, 2) |
| T2D_4_10 | (1, 4, 2) | | (1, 1, 4) | (1, 4, 1) | | (1, 1, 1) | | (1, 1, 1) | | (1, 1, 1) | | (1, 2, 8) | | (1, 1, 1) | | (1, 1, 1) | | (1, 1, 8) | | (1, 1, 8) | | (1, 8, 1) | (56, 128) | | | (128, 2) |
| T2D_4_11 | (1, 4, 2) | | (1, 1, 4) | (1, 4, 1) | | (1, 1, 1) | | (1, 1, 1) | | (1, 1, 1) | | (1, 2, 8) | | (1, 1, 1) | | (1, 1, 1) | | (1, 1, 8) | | (1, 1, 8) | | (1, 8, 1) | (58, 128) | | | (128, 2) |
| T2D_4_12 | (1, 4, 2) | | (1, 1, 4) | (1, 4, 1) | | (1, 1, 1) | | (1, 1, 1) | | (1, 1, 1) | | (1, 2, 8) | | (1, 1, 1) | | (1, 1, 1) | | (1, 1, 8) | | (1, 1, 8) | | (1, 8, 1) | (60, 128) | | | (128, 2) |
| T2D_4_13 | (1, 4, 2) | | (1, 1, 4) | (1, 4, 1) | | (1, 1, 1) | | (1, 1, 1) | | (1, 1, 1) | | (1, 2, 8) | | (1, 1, 1) | | (1, 1, 1) | | (1, 1, 8) | | (1, 1, 8) | | (1, 8, 1) | (62, 128) | | | (128, 2) |
| T2D_4_14 | (1, 4, 2) | | (1, 1, 4) | (1, 4, 1) | | (1, 1, 1) | | (1, 1, 1) | | (1, 1, 1) | | (1, 2, 8) | | (1, 1, 1) | | (1, 1, 1) | | (1, 1, 8) | | (1, 1, 8) | | (1, 8, 1) | (64, 128) | | | (128, 2) |
| T2D_4_6 | (1, 4, 2) | | (1, 1, 4) | (1, 4, 1) | | (1, 1, 1) | | (1, 1, 1) | | (1, 1, 1) | | (1, 2, 8) | | (1, 1, 1) | | (1, 1, 1) | | (1, 1, 8) | | (1, 1, 8) | | (1, 8, 1) | (48, 128) | | | (128, 2) |
| T2D_4_7 | (1, 4, 2) | | (1, 1, 4) | (1, 4, 1) | | (1, 1, 1) | | (1, 1, 1) | | (1, 1, 1) | | (1, 2, 8) | | (1, 1, 1) | | (1, 1, 1) | | (1, 1, 8) | | (1, 1, 8) | | (1, 8, 1) | (50, 128) | | | (128, 2) |
| T2D_4_8 | (1, 4, 2) | | (1, 1, 4) | (1, 4, 1) | | (1, 1, 1) | | (1, 1, 1) | | (1, 1, 1) | | (1, 2, 8) | | (1, 1, 1) | | (1, 1, 1) | | (1, 1, 8) | | (1, 1, 8) | | (1, 8, 1) | (52, 128) | | | (128, 2) |
| T2D_4_9 | (1, 4, 2) | | (1, 1, 4) | (1, 4, 1) | | (1, 1, 1) | | (1, 1, 1) | | (1, 1, 1) | | (1, 2, 8) | | (1, 1, 1) | | (1, 1, 1) | | (1, 1, 8) | | (1, 1, 8) | | (1, 8, 1) | (54, 128) | | | (128, 2) |
| T2D_5_10 | (1, 4, 2) | | (1, 1, 4) | (1, 4, 1) | | (1, 1, 1) | | (1, 1, 1) | | (1, 1, 1) | | (1, 2, 8) | | (1, 1, 1) | | (1, 1, 1) | | (1, 1, 8) | | (1, 1, 8) | | (1, 8, 1) | (54, 128) | | | (128, 2) |
| T2D_5_11 | (1, 4, 2) | | (1, 1, 4) | (1, 4, 1) | | (1, 1, 1) | | (1, 1, 1) | | (1, 1, 1) | | (1, 2, 8) | | (1, 1, 1) | | (1, 1, 1) | | (1, 1, 8) | | (1, 1, 8) | | (1, 8, 1) | (56, 128) | | | (128, 2) |
| T2D_5_12 | (1, 4, 2) | | (1, 1, 4) | (1, 4, 1) | | (1, 1, 1) | | (1, 1, 1) | | (1, 1, 1) | | (1, 2, 8) | | (1, 1, 1) | | (1, 1, 1) | | (1, 1, 8) | | (1, 1, 8) | | (1, 8, 1) | (58, 128) | | | (128, 2) |
| T2D_5_13 | (1, 4, 2) | | (1, 1, 4) | (1, 4, 1) | | (1, 1, 1) | | (1, 1, 1) | | (1, 1, 1) | | (1, 2, 8) | | (1, 1, 1) | | (1, 1, 1) | | (1, 1, 8) | | (1, 1, 8) | | (1, 8, 1) | (60, 128) | | | (128, 2) |
| T2D_5_14 | (1, 4, 2) | | (1, 1, 4) | (1, 4, 1) | | (1, 1, 1) | | (1, 1, 1) | | (1, 1, 1) | | (1, 2, 8) | | (1, 1, 1) | | (1, 1, 1) | | (1, 1, 8) | | (1, 1, 8) | | (1, 8, 1) | (62, 128) | | | (128, 2) |
| T2D_5_7 | (1, 4, 2) | | (1, 1, 4) | (1, 4, 1) | | (1, 1, 1) | | (1, 1, 1) | | (1, 1, 1) | | (1, 2, 8) | | (1, 1, 1) | | (1, 1, 1) | | (1, 1, 8) | | (1, 1, 8) | | (1, 8, 1) | (48, 128) | | | (128, 2) |
| T2D_5_8 | (1, 4, 2) | | (1, 1, 4) | (1, 4, 1) | | (1, 1, 1) | | (1, 1, 1) | | (1, 1, 1) | | (1, 2, 8) | | (1, 1, 1) | | (1, 1, 1) | | (1, 1, 8) | | (1, 1, 8) | | (1, 8, 1) | (50, 128) | | | (128, 2) |
| T2D_5_9 | (1, 4, 2) | | (1, 1, 4) | (1, 4, 1) | | (1, 1, 1) | | (1, 1, 1) | | (1, 1, 1) | | (1, 2, 8) | | (1, 1, 1) | | (1, 1, 1) | | (1, 1, 8) | | (1, 1, 8) | | (1, 8, 1) | (52, 128) | | | (128, 2) |
| T2D_6_10 | (1, 4, 2) | | (1, 1, 4) | (1, 4, 1) | | (1, 1, 1) | | (1, 1, 1) | | (1, 1, 1) | | (1, 2, 8) | | (1, 1, 1) | | (1, 1, 1) | | (1, 1, 8) | | (1, 1, 8) | | (1, 8, 1) | (52, 128) | | | (128, 2) |
| T2D_6_11 | (1, 4, 2) | | (1, 1, 4) | (1, 4, 1) | | (1, 1, 1) | | (1, 1, 1) | | (1, 1, 1) | | (1, 2, 8) | | (1, 1, 1) | | (1, 1, 1) | | (1, 1, 8) | | (1, 1, 8) | | (1, 8, 1) | (54, 128) | | | (128, 2) |
| T2D_6_12 | (1, 4, 2) | | (1, 1, 4) | (1, 4, 1) | | (1, 1, 1) | | (1, 1, 1) | | (1, 1, 1) | | (1, 2, 8) | | (1, 1, 1) | | (1, 1, 1) | | (1, 1, 8) | | (1, 1, 8) | | (1, 8, 1) | (56, 128) | | | (128, 2) |
| T2D_6_13 | (1, 4, 2) | | (1, 1, 4) | (1, 4, 1) | | (1, 1, 1) | | (1, 1, 1) | | (1, 1, 1) | | (1, 2, 8) | | (1, 1, 1) | | (1, 1, 1) | | (1, 1, 8) | | (1, 1, 8) | | (1, 8, 1) | (58, 128) | | | (128, 2) |
| T2D_6_14 | (1, 4, 2) | | (1, 1, 4) | (1, 4, 1) | | (1, 1, 1) | | (1, 1, 1) | | (1, 1, 1) | | (1, 2, 8) | | (1, 1, 1) | | (1, 1, 1) | | (1, 1, 8) | | (1, 1, 8) | | (1, 8, 1) | (60, 128) | | | (128, 2) |
| T2D_6_8 | (1, 4, 2) | | (1, 1, 4) | (1, 4, 1) | | (1, 1, 1) | | (1, 1, 1) | | (1, 1, 1) | | (1, 2, 8) | | (1, 1, 1) | | (1, 1, 1) | | (1, 1, 8) | | (1, 1, 8) | | (1, 8, 1) | (48, 128) | | | (128, 2) |
| T2D_6_9 | (1, 4, 2) | | (1, 1, 4) | (1, 4, 1) | | (1, 1, 1) | | (1, 1, 1) | | (1, 1, 1) | | (1, 2, 8) | | (1, 1, 1) | | (1, 1, 1) | | (1, 1, 8) | | (1, 1, 8) | | (1, 8, 1) | (50, 128) | | | (128, 2) |
| T2D_7_10 | (1, 4, 2) | | (1, 1, 4) | (1, 4, 1) | | (1, 1, 1) | | (1, 1, 1) | | (1, 1, 1) | | (1, 2, 8) | | (1, 1, 1) | | (1, 1, 1) | | (1, 1, 8) | | (1, 1, 8) | | (1, 8, 1) | (50, 128) | | | (128, 2) |
| T2D_7_11 | (1, 4, 2) | | (1, 1, 4) | (1, 4, 1) | | (1, 1, 1) | | (1, 1, 1) | | (1, 1, 1) | | (1, 2, 8) | | (1, 1, 1) | | (1, 1, 1) | | (1, 1, 8) | | (1, 1, 8) | | (1, 8, 1) | (52, 128) | | | (128, 2) |
| T2D_7_12 | (1, 4, 2) | | (1, 1, 4) | (1, 4, 1) | | (1, 1, 1) | | (1, 1, 1) | | (1, 1, 1) | | (1, 2, 8) | | (1, 1, 1) | | (1, 1, 1) | | (1, 1, 8) | | (1, 1, 8) | | (1, 8, 1) | (54, 128) | | | (128, 2) |
| T2D_7_13 | (1, 4, 2) | | (1, 1, 4) | (1, 4, 1) | | (1, 1, 1) | | (1, 1, 1) | | (1, 1, 1) | | (1, 2, 8) | | (1, 1, 1) | | (1, 1, 1) | | (1, 1, 8) | | (1, 1, 8) | | (1, 8, 1) | (56, 128) | | | (128, 2) |
| T2D_7_14 | (1, 4, 2) | | (1, 1, 4) | (1, 4, 1) | | (1, 1, 1) | | (1, 1, 1) | | (1, 1, 1) | | (1, 2, 8) | | (1, 1, 1) | | (1, 1, 1) | | (1, 1, 8) | | (1, 1, 8) | | (1, 8, 1) | (58, 128) | | | (128, 2) |
| T2D_7_9 | (1, 4, 2) | | (1, 1, 4) | (1, 4, 1) | | (1, 1, 1) | | (1, 1, 1) | | (1, 1, 1) | | (1, 2, 8) | | (1, 1, 1) | | (1, 1, 1) | | (1, 1, 8) | | (1, 1, 8) | | (1, 8, 1) | (48, 128) | | | (128, 2) |
| T2D_8_10 | (1, 4, 2) | | (1, 1, 4) | (1, 4, 1) | | (1, 1, 1) | | (1, 1, 1) | | (1, 1, 1) | | (1, 2, 8) | | (1, 1, 1) | | (1, 1, 1) | | (1, 1, 8) | | (1, 1, 8) | | (1, 8, 1) | (48, 128) | | | (128, 2) |
| T2D_8_11 | (1, 4, 2) | | (1, 1, 4) | (1, 4, 1) | | (1, 1, 1) | | (1, 1, 1) | | (1, 1, 1) | | (1, 2, 8) | | (1, 1, 1) | | (1, 1, 1) | | (1, 1, 8) | | (1, 1, 8) | | (1, 8, 1) | (50, 128) | | | (128, 2) |
| T2D_8_12 | (1, 4, 2) | | (1, 1, 4) | (1, 4, 1) | | (1, 1, 1) | | (1, 1, 1) | | (1, 1, 1) | | (1, 2, 8) | | (1, 1, 1) | | (1, 1, 1) | | (1, 1, 8) | | (1, 1, 8) | | (1, 8, 1) | (52, 128) | | | (128, 2) |
| T2D_8_13 | (1, 4, 2) | | (1, 1, 4) | (1, 4, 1) | | (1, 1, 1) | | (1, 1, 1) | | (1, 1, 1) | | (1, 2, 8) | | (1, 1, 1) | | (1, 1, 1) | | (1, 1, 8) | | (1, 1, 8) | | (1, 8, 1) | (54, 128) | | | (128, 2) |
| T2D_8_14 | (1, 4, 2) | | (1, 1, 4) | (1, 4, 1) | | (1, 1, 1) | | (1, 1, 1) | | (1, 1, 1) | | (1, 2, 8) | | (1, 1, 1) | | (1, 1, 1) | | (1, 1, 8) | | (1, 1, 8) | | (1, 8, 1) | (56, 128) | | | (128, 2) |
| T2D_9_11 | (1, 4, 2) | | (1, 1, 4) | (1, 4, 1) | | (1, 1, 1) | | (1, 1, 1) | | (1, 1, 1) | | (1, 2, 8) | | (1, 1, 1) | | (1, 1, 1) | | (1, 1, 8) | | (1, 1, 8) | | (1, 8, 1) | (48, 128) | | | (128, 2) |
| T2D_9_12 | (1, 4, 2) | | (1, 1, 4) | (1, 4, 1) | | (1, 1, 1) | | (1, 1, 1) | | (1, 1, 1) | | (1, 2, 8) | | (1, 1, 1) | | (1, 1, 1) | | (1, 1, 8) | | (1, 1, 8) | | (1, 8, 1) | (50, 128) | | | (128, 2) |
| T2D_9_13 | (1, 4, 2) | | (1, 1, 4) | (1, 4, 1) | | (1, 1, 1) | | (1, 1, 1) | | (1, 1, 1) | | (1, 2, 8) | | (1, 1, 1) | | (1, 1, 1) | | (1, 1, 8) | | (1, 1, 8) | | (1, 8, 1) | (52, 128) | | | (128, 2) |
| T2D_9_14 | (1, 4, 2) | | (1, 1, 4) | (1, 4, 1) | | (1, 1, 1) | | (1, 1, 1) | | (1, 1, 1) | | (1, 2, 8) | | (1, 1, 1) | | (1, 1, 1) | | (1, 1, 8) | | (1, 1, 8) | | (1, 8, 1) | (54, 128) | | | (128, 2) |
| Metabolic_10_12 | (1, 4, 2) | | (1, 1, 4) | (1, 4, 1) | | (1, 1, 1) | | (1, 1, 1) | | (1, 1, 1) | | (1, 2, 8) | | (1, 1, 1) | | (1, 1, 1) | | (1, 1, 8) | | (1, 1, 8) | | (1, 8, 1) | (112, 128) | | | (128, 2) |
| Metabolic_10_13 | (1, 4, 2) | | (1, 1, 4) | (1, 4, 1) | | (1, 1, 1) | | (1, 1, 1) | | (1, 1, 1) | | (1, 2, 8) | | (1, 1, 1) | | (1, 1, 1) | | (1, 1, 8) | | (1, 1, 8) | | (1, 8, 1) | (114, 128) | | | (128, 2) |
| Metabolic_11_13 | (1, 4, 2) | | (1, 1, 4) | (1, 4, 1) | | (1, 1, 1) | | (1, 1, 1) | | (1, 1, 1) | | (1, 2, 8) | | (1, 1, 1) | | (1, 1, 1) | | (1, 1, 8) | | (1, 1, 8) | | (1, 8, 1) | (112, 128) | | | (128, 2) |
| Metabolic_2_10 | (1, 4, 2) | | (1, 1, 4) | (1, 4, 1) | | (1, 1, 1) | | (1, 1, 1) | | (1, 1, 1) | | (1, 2, 8) | | (1, 1, 1) | | (1, 1, 1) | | (1, 1, 8) | | (1, 1, 8) | | (1, 8, 1) | (124, 128) | | | (128, 2) |
| Metabolic_2_11 | (1, 4, 2) | | (1, 1, 4) | (1, 4, 1) | | (1, 1, 1) | | (1, 1, 1) | | (1, 1, 1) | | (1, 2, 8) | | (1, 1, 1) | | (1, 1, 1) | | (1, 1, 8) | | (1, 1, 8) | | (1, 8, 1) | (126, 128) | | | (128, 2) |
| Metabolic_2_12 | (1, 4, 2) | | (1, 1, 4) | (1, 4, 1) | | (1, 1, 1) | | (1, 1, 1) | | (1, 1, 1) | | (1, 2, 8) | | (1, 1, 1) | | (1, 1, 1) | | (1, 1, 8) | | (1, 1, 8) | | (1, 8, 1) | (128, 128) | | | (128, 2) |
| Metabolic_2_13 | (1, 4, 2) | | (1, 1, 4) | (1, 4, 1) | | (1, 1, 1) | | (1, 1, 1) | | (1, 1, 1) | | (1, 2, 8) | | (1, 1, 1) | | (1, 1, 1) | | (1, 1, 8) | | (1, 1, 8) | | (1, 8, 1) | (130, 128) | | | (128, 2) |
| Metabolic_2_4 | (1, 4, 2) | | (1, 1, 4) | (1, 4, 1) | | (1, 1, 1) | | (1, 1, 1) | | (1, 1, 1) | | (1, 2, 8) | | (1, 1, 1) | | (1, 1, 1) | | (1, 1, 8) | | (1, 1, 8) | | (1, 8, 1) | (112, 128) | | | (128, 2) |
| Metabolic_2_5 | (1, 4, 2) | | (1, 1, 4) | (1, 4, 1) | | (1, 1, 1) | | (1, 1, 1) | | (1, 1, 1) | | (1, 2, 8) | | (1, 1, 1) | | (1, 1, 1) | | (1, 1, 8) | | (1, 1, 8) | | (1, 8, 1) | (114, 128) | | | (128, 2) |
| Metabolic_2_6 | (1, 4, 2) | | (1, 1, 4) | (1, 4, 1) | | (1, 1, 1) | | (1, 1, 1) | | (1, 1, 1) | | (1, 2, 8) | | (1, 1, 1) | | (1, 1, 1) | | (1, 1, 8) | | (1, 1, 8) | | (1, 8, 1) | (116, 128) | | | (128, 2) |
| Metabolic_2_7 | (1, 4, 2) | | (1, 1, 4) | (1, 4, 1) | | (1, 1, 1) | | (1, 1, 1) | | (1, 1, 1) | | (1, 2, 8) | | (1, 1, 1) | | (1, 1, 1) | | (1, 1, 8) | | (1, 1, 8) | | (1, 8, 1) | (118, 128) | | | (128, 2) |
| Metabolic_2_8 | (1, 4, 2) | | (1, 1, 4) | (1, 4, 1) | | (1, 1, 1) | | (1, 1, 1) | | (1, 1, 1) | | (1, 2, 8) | | (1, 1, 1) | | (1, 1, 1) | | (1, 1, 8) | | (1, 1, 8) | | (1, 8, 1) | (120, 128) | | | (128, 2) |
| Metabolic_2_9 | (1, 4, 2) | | (1, 1, 4) | (1, 4, 1) | | (1, 1, 1) | | (1, 1, 1) | | (1, 1, 1) | | (1, 2, 8) | | (1, 1, 1) | | (1, 1, 1) | | (1, 1, 8) | | (1, 1, 8) | | (1, 8, 1) | (122, 128) | | | (128, 2) |
| Metabolic_3_10 | (1, 4, 2) | | (1, 1, 4) | (1, 4, 1) | | (1, 1, 1) | | (1, 1, 1) | | (1, 1, 1) | | (1, 2, 8) | | (1, 1, 1) | | (1, 1, 1) | | (1, 1, 8) | | (1, 1, 8) | | (1, 8, 1) | (122, 128) | | | (128, 2) |
| Metabolic_3_11 | (1, 4, 2) | | (1, 1, 4) | (1, 4, 1) | | (1, 1, 1) | | (1, 1, 1) | | (1, 1, 1) | | (1, 2, 8) | | (1, 1, 1) | | (1, 1, 1) | | (1, 1, 8) | | (1, 1, 8) | | (1, 8, 1) | (124, 128) | | | (128, 2) |
| Metabolic_3_12 | (1, 4, 2) | | (1, 1, 4) | (1, 4, 1) | | (1, 1, 1) | | (1, 1, 1) | | (1, 1, 1) | | (1, 2, 8) | | (1, 1, 1) | | (1, 1, 1) | | (1, 1, 8) | | (1, 1, 8) | | (1, 8, 1) | (126, 128) | | | (128, 2) |
| Metabolic_3_13 | (1, 4, 2) | | (1, 1, 4) | (1, 4, 1) | | (1, 1, 1) | | (1, 1, 1) | | (1, 1, 1) | | (1, 2, 8) | | (1, 1, 1) | | (1, 1, 1) | | (1, 1, 8) | | (1, 1, 8) | | (1, 8, 1) | (128, 128) | | | (128, 2) |
| Metabolic_3_5 | (1, 4, 2) | | (1, 1, 4) | (1, 4, 1) | | (1, 1, 1) | | (1, 1, 1) | | (1, 1, 1) | | (1, 2, 8) | | (1, 1, 1) | | (1, 1, 1) | | (1, 1, 8) | | (1, 1, 8) | | (1, 8, 1) | (112, 128) | | | (128, 2) |
| Metabolic_3_6 | (1, 4, 2) | | (1, 1, 4) | (1, 4, 1) | | (1, 1, 1) | | (1, 1, 1) | | (1, 1, 1) | | (1, 2, 8) | | (1, 1, 1) | | (1, 1, 1) | | (1, 1, 8) | | (1, 1, 8) | | (1, 8, 1) | (114, 128) | | | (128, 2) |
| Metabolic_3_7 | (1, 4, 2) | | (1, 1, 4) | (1, 4, 1) | | (1, 1, 1) | | (1, 1, 1) | | (1, 1, 1) | | (1, 2, 8) | | (1, 1, 1) | | (1, 1, 1) | | (1, 1, 8) | | (1, 1, 8) | | (1, 8, 1) | (116, 128) | | | (128, 2) |
| Metabolic_3_8 | (1, 4, 2) | | (1, 1, 4) | (1, 4, 1) | | (1, 1, 1) | | (1, 1, 1) | | (1, 1, 1) | | (1, 2, 8) | | (1, 1, 1) | | (1, 1, 1) | | (1, 1, 8) | | (1, 1, 8) | | (1, 8, 1) | (118, 128) | | | (128, 2) |
| Metabolic_3_9 | (1, 4, 2) | | (1, 1, 4) | (1, 4, 1) | | (1, 1, 1) | | (1, 1, 1) | | (1, 1, 1) | | (1, 2, 8) | | (1, 1, 1) | | (1, 1, 1) | | (1, 1, 8) | | (1, 1, 8) | | (1, 8, 1) | (120, 128) | | | (128, 2) |
| Metabolic_4_10 | (1, 4, 2) | | (1, 1, 4) | (1, 4, 1) | | (1, 1, 1) | | (1, 1, 1) | | (1, 1, 1) | | (1, 2, 8) | | (1, 1, 1) | | (1, 1, 1) | | (1, 1, 8) | | (1, 1, 8) | | (1, 8, 1) | (120, 128) | | | (128, 2) |
| Metabolic_4_11 | (1, 4, 2) | | (1, 1, 4) | (1, 4, 1) | | (1, 1, 1) | | (1, 1, 1) | | (1, 1, 1) | | (1, 2, 8) | | (1, 1, 1) | | (1, 1, 1) | | (1, 1, 8) | | (1, 1, 8) | | (1, 8, 1) | (122, 128) | | | (128, 2) |
| Metabolic_4_12 | (1, 4, 2) | | (1, 1, 4) | (1, 4, 1) | | (1, 1, 1) | | (1, 1, 1) | | (1, 1, 1) | | (1, 2, 8) | | (1, 1, 1) | | (1, 1, 1) | | (1, 1, 8) | | (1, 1, 8) | | (1, 8, 1) | (124, 128) | | | (128, 2) |
| Metabolic_4_13 | (1, 4, 2) | | (1, 1, 4) | (1, 4, 1) | | (1, 1, 1) | | (1, 1, 1) | | (1, 1, 1) | | (1, 2, 8) | | (1, 1, 1) | | (1, 1, 1) | | (1, 1, 8) | | (1, 1, 8) | | (1, 8, 1) | (126, 128) | | | (128, 2) |
| Metabolic_4_6 | (1, 4, 2) | | (1, 1, 4) | (1, 4, 1) | | (1, 1, 1) | | (1, 1, 1) | | (1, 1, 1) | | (1, 2, 8) | | (1, 1, 1) | | (1, 1, 1) | | (1, 1, 8) | | (1, 1, 8) | | (1, 8, 1) | (112, 128) | | | (128, 2) |
| Metabolic_4_7 | (1, 4, 2) | | (1, 1, 4) | (1, 4, 1) | | (1, 1, 1) | | (1, 1, 1) | | (1, 1, 1) | | (1, 2, 8) | | (1, 1, 1) | | (1, 1, 1) | | (1, 1, 8) | | (1, 1, 8) | | (1, 8, 1) | (114, 128) | | | (128, 2) |
| Metabolic_4_8 | (1, 4, 2) | | (1, 1, 4) | (1, 4, 1) | | (1, 1, 1) | | (1, 1, 1) | | (1, 1, 1) | | (1, 2, 8) | | (1, 1, 1) | | (1, 1, 1) | | (1, 1, 8) | | (1, 1, 8) | | (1, 8, 1) | (116, 128) | | | (128, 2) |
| Metabolic_4_9 | (1, 4, 2) | | (1, 1, 4) | (1, 4, 1) | | (1, 1, 1) | | (1, 1, 1) | | (1, 1, 1) | | (1, 2, 8) | | (1, 1, 1) | | (1, 1, 1) | | (1, 1, 8) | | (1, 1, 8) | | (1, 8, 1) | (118, 128) | | | (128, 2) |
| Metabolic_5_10 | (1, 4, 2) | | (1, 1, 4) | (1, 4, 1) | | (1, 1, 1) | | (1, 1, 1) | | (1, 1, 1) | | (1, 2, 8) | | (1, 1, 1) | | (1, 1, 1) | | (1, 1, 8) | | (1, 1, 8) | | (1, 8, 1) | (118, 128) | | | (128, 2) |
| Metabolic_5_11 | (1, 4, 2) | | (1, 1, 4) | (1, 4, 1) | | (1, 1, 1) | | (1, 1, 1) | | (1, 1, 1) | | (1, 2, 8) | | (1, 1, 1) | | (1, 1, 1) | | (1, 1, 8) | | (1, 1, 8) | | (1, 8, 1) | (120, 128) | | | (128, 2) |
| Metabolic_5_13 | (1, 4, 2) | | (1, 1, 4) | (1, 4, 1) | | (1, 1, 1) | | (1, 1, 1) | | (1, 1, 1) | | (1, 2, 8) | | (1, 1, 1) | | (1, 1, 1) | | (1, 1, 8) | | (1, 1, 8) | | (1, 8, 1) | (124, 128) | | | (128, 2) |
| Metabolic_5_7 | (1, 4, 2) | | (1, 1, 4) | (1, 4, 1) | | (1, 1, 1) | | (1, 1, 1) | | (1, 1, 1) | | (1, 2, 8) | | (1, 1, 1) | | (1, 1, 1) | | (1, 1, 8) | | (1, 1, 8) | | (1, 8, 1) | (112, 128) | | | (128, 2) |
| Metabolic_5_8 | (1, 4, 2) | | (1, 1, 4) | (1, 4, 1) | | (1, 1, 1) | | (1, 1, 1) | | (1, 1, 1) | | (1, 2, 8) | | (1, 1, 1) | | (1, 1, 1) | | (1, 1, 8) | | (1, 1, 8) | | (1, 8, 1) | (114, 128) | | | (128, 2) |
| Metabolic_5_9 | (1, 4, 2) | | (1, 1, 4) | (1, 4, 1) | | (1, 1, 1) | | (1, 1, 1) | | (1, 1, 1) | | (1, 2, 8) | | (1, 1, 1) | | (1, 1, 1) | | (1, 1, 8) | | (1, 1, 8) | | (1, 8, 1) | (116, 128) | | | (128, 2) |
| Metabolic_6_10 | (1, 4, 2) | | (1, 1, 4) | (1, 4, 1) | | (1, 1, 1) | | (1, 1, 1) | | (1, 1, 1) | | (1, 2, 8) | | (1, 1, 1) | | (1, 1, 1) | | (1, 1, 8) | | (1, 1, 8) | | (1, 8, 1) | (116, 128) | | | (128, 2) |
| Metabolic_6_11 | (1, 4, 2) | | (1, 1, 4) | (1, 4, 1) | | (1, 1, 1) | | (1, 1, 1) | | (1, 1, 1) | | (1, 2, 8) | | (1, 1, 1) | | (1, 1, 1) | | (1, 1, 8) | | (1, 1, 8) | | (1, 8, 1) | (118, 128) | | | (128, 2) |
| Metabolic_6_12 | (1, 4, 2) | | (1, 1, 4) | (1, 4, 1) | | (1, 1, 1) | | (1, 1, 1) | | (1, 1, 1) | | (1, 2, 8) | | (1, 1, 1) | | (1, 1, 1) | | (1, 1, 8) | | (1, 1, 8) | | (1, 8, 1) | (120, 128) | | | (128, 2) |
| Metabolic_6_13 | (1, 4, 2) | | (1, 1, 4) | (1, 4, 1) | | (1, 1, 1) | | (1, 1, 1) | | (1, 1, 1) | | (1, 2, 8) | | (1, 1, 1) | | (1, 1, 1) | | (1, 1, 8) | | (1, 1, 8) | | (1, 8, 1) | (122, 128) | | | (128, 2) |
| Metabolic_6_8 | (1, 4, 2) | | (1, 1, 4) | (1, 4, 1) | | (1, 1, 1) | | (1, 1, 1) | | (1, 1, 1) | | (1, 2, 8) | | (1, 1, 1) | | (1, 1, 1) | | (1, 1, 8) | | (1, 1, 8) | | (1, 8, 1) | (112, 128) | | | (128, 2) |
| Metabolic_6_9 | (1, 4, 2) | | (1, 1, 4) | (1, 4, 1) | | (1, 1, 1) | | (1, 1, 1) | | (1, 1, 1) | | (1, 2, 8) | | (1, 1, 1) | | (1, 1, 1) | | (1, 1, 8) | | (1, 1, 8) | | (1, 8, 1) | (114, 128) | | | (128, 2) |
| Metabolic_7_10 | (1, 4, 2) | | (1, 1, 4) | (1, 4, 1) | | (1, 1, 1) | | (1, 1, 1) | | (1, 1, 1) | | (1, 2, 8) | | (1, 1, 1) | | (1, 1, 1) | | (1, 1, 8) | | (1, 1, 8) | | (1, 8, 1) | (114, 128) | | | (128, 2) |
| Metabolic_7_11 | (1, 4, 2) | | (1, 1, 4) | (1, 4, 1) | | (1, 1, 1) | | (1, 1, 1) | | (1, 1, 1) | | (1, 2, 8) | | (1, 1, 1) | | (1, 1, 1) | | (1, 1, 8) | | (1, 1, 8) | | (1, 8, 1) | (116, 128) | | | (128, 2) |
| Metabolic_7_12 | (1, 4, 2) | | (1, 1, 4) | (1, 4, 1) | | (1, 1, 1) | | (1, 1, 1) | | (1, 1, 1) | | (1, 2, 8) | | (1, 1, 1) | | (1, 1, 1) | | (1, 1, 8) | | (1, 1, 8) | | (1, 8, 1) | (118, 128) | | | (128, 2) |
| Metabolic_7_13 | (1, 4, 2) | | (1, 1, 4) | (1, 4, 1) | | (1, 1, 1) | | (1, 1, 1) | | (1, 1, 1) | | (1, 2, 8) | | (1, 1, 1) | | (1, 1, 1) | | (1, 1, 8) | | (1, 1, 8) | | (1, 8, 1) | (120, 128) | | | (128, 2) |
| Metabolic_7_9 | (1, 4, 2) | | (1, 1, 4) | (1, 4, 1) | | (1, 1, 1) | | (1, 1, 1) | | (1, 1, 1) | | (1, 2, 8) | | (1, 1, 1) | | (1, 1, 1) | | (1, 1, 8) | | (1, 1, 8) | | (1, 8, 1) | (112, 128) | | | (128, 2) |
| Metabolic_8_10 | (1, 4, 2) | | (1, 1, 4) | (1, 4, 1) | | (1, 1, 1) | | (1, 1, 1) | | (1, 1, 1) | | (1, 2, 8) | | (1, 1, 1) | | (1, 1, 1) | | (1, 1, 8) | | (1, 1, 8) | | (1, 8, 1) | (112, 128) | | | (128, 2) |
| Metabolic_8_11 | (1, 4, 2) | | (1, 1, 4) | (1, 4, 1) | | (1, 1, 1) | | (1, 1, 1) | | (1, 1, 1) | | (1, 2, 8) | | (1, 1, 1) | | (1, 1, 1) | | (1, 1, 8) | | (1, 1, 8) | | (1, 8, 1) | (114, 128) | | | (128, 2) |
| Metabolic_8_12 | (1, 4, 2) | | (1, 1, 4) | (1, 4, 1) | | (1, 1, 1) | | (1, 1, 1) | | (1, 1, 1) | | (1, 2, 8) | | (1, 1, 1) | | (1, 1, 1) | | (1, 1, 8) | | (1, 1, 8) | | (1, 8, 1) | (116, 128) | | | (128, 2) |
| Metabolic_8_13 | (1, 4, 2) | | (1, 1, 4) | (1, 4, 1) | | (1, 1, 1) | | (1, 1, 1) | | (1, 1, 1) | | (1, 2, 8) | | (1, 1, 1) | | (1, 1, 1) | | (1, 1, 8) | | (1, 1, 8) | | (1, 8, 1) | (118, 128) | | | (128, 2) |
| Metabolic_9_11 | (1, 4, 2) | | (1, 1, 4) | (1, 4, 1) | | (1, 1, 1) | | (1, 1, 1) | | (1, 1, 1) | | (1, 2, 8) | | (1, 1, 1) | | (1, 1, 1) | | (1, 1, 8) | | (1, 1, 8) | | (1, 8, 1) | (112, 128) | | | (128, 2) |
| Metabolic_9_12 | (1, 4, 2) | | (1, 1, 4) | (1, 4, 1) | | (1, 1, 1) | | (1, 1, 1) | | (1, 1, 1) | | (1, 2, 8) | | (1, 1, 1) | | (1, 1, 1) | | (1, 1, 8) | | (1, 1, 8) | | (1, 8, 1) | (114, 128) | | | (128, 2) |
| Metabolic_9_13 | (1, 4, 2) | | (1, 1, 4) | (1, 4, 1) | | (1, 1, 1) | | (1, 1, 1) | | (1, 1, 1) | | (1, 2, 8) | | (1, 1, 1) | | (1, 1, 1) | | (1, 1, 8) | | (1, 1, 8) | | (1, 8, 1) | (116, 128) | | | (128, 2) |
| Prediabetes_10_12 | (1, 4, 2) | | (1, 1, 4) | (1, 4, 1) | | (1, 1, 1) | | (1, 1, 1) | | (1, 1, 1) | | (1, 2, 8) | | (1, 1, 1) | | (1, 1, 1) | | (1, 1, 8) | | (1, 1, 8) | | (1, 8, 1) | (261, 128) | | | (128, 2) |
| Prediabetes_10_13 | (1, 4, 2) | | (1, 1, 4) | (1, 4, 1) | | (1, 1, 1) | | (1, 1, 1) | | (1, 1, 1) | | (1, 2, 8) | | (1, 1, 1) | | (1, 1, 1) | | (1, 1, 8) | | (1, 1, 8) | | (1, 8, 1) | (263, 128) | | | (128, 2) |
| Prediabetes_11_13 | (1, 4, 2) | | (1, 1, 4) | (1, 4, 1) | | (1, 1, 1) | | (1, 1, 1) | | (1, 1, 1) | | (1, 2, 8) | | (1, 1, 1) | | (1, 1, 1) | | (1, 1, 8) | | (1, 1, 8) | | (1, 8, 1) | (261, 128) | | | (128, 2) |
| Prediabetes_2_10 | (1, 4, 2) | | (1, 1, 4) | (1, 4, 1) | | (1, 1, 1) | | (1, 1, 1) | | (1, 1, 1) | | (1, 2, 8) | | (1, 1, 1) | | (1, 1, 1) | | (1, 1, 8) | | (1, 1, 8) | | (1, 8, 1) | (273, 128) | | | (128, 2) |
| Prediabetes_2_12 | (1, 4, 2) | | (1, 1, 4) | (1, 4, 1) | | (1, 1, 1) | | (1, 1, 1) | | (1, 1, 1) | | (1, 2, 8) | | (1, 1, 1) | | (1, 1, 1) | | (1, 1, 8) | | (1, 1, 8) | | (1, 8, 1) | (277, 128) | | | (128, 2) |
| Prediabetes_2_13 | (1, 4, 2) | | (1, 1, 4) | (1, 4, 1) | | (1, 1, 1) | | (1, 1, 1) | | (1, 1, 1) | | (1, 2, 8) | | (1, 1, 1) | | (1, 1, 1) | | (1, 1, 8) | | (1, 1, 8) | | (1, 8, 1) | (279, 128) | | | (128, 2) |
| Prediabetes_2_4 | (1, 4, 2) | | (1, 1, 4) | (1, 4, 1) | | (1, 1, 1) | | (1, 1, 1) | | (1, 1, 1) | | (1, 2, 8) | | (1, 1, 1) | | (1, 1, 1) | | (1, 1, 8) | | (1, 1, 8) | | (1, 8, 1) | (261, 128) | | | (128, 2) |
| Prediabetes_2_5 | (1, 4, 2) | | (1, 1, 4) | (1, 4, 1) | | (1, 1, 1) | | (1, 1, 1) | | (1, 1, 1) | | (1, 2, 8) | | (1, 1, 1) | | (1, 1, 1) | | (1, 1, 8) | | (1, 1, 8) | | (1, 8, 1) | (263, 128) | | | (128, 2) |
| Prediabetes_2_6 | (1, 4, 2) | | (1, 1, 4) | (1, 4, 1) | | (1, 1, 1) | | (1, 1, 1) | | (1, 1, 1) | | (1, 2, 8) | | (1, 1, 1) | | (1, 1, 1) | | (1, 1, 8) | | (1, 1, 8) | | (1, 8, 1) | (265, 128) | | | (128, 2) |
| Prediabetes_2_7 | (1, 4, 2) | | (1, 1, 4) | (1, 4, 1) | | (1, 1, 1) | | (1, 1, 1) | | (1, 1, 1) | | (1, 2, 8) | | (1, 1, 1) | | (1, 1, 1) | | (1, 1, 8) | | (1, 1, 8) | | (1, 8, 1) | (267, 128) | | | (128, 2) |
| Prediabetes_2_8 | (1, 4, 2) | | (1, 1, 4) | (1, 4, 1) | | (1, 1, 1) | | (1, 1, 1) | | (1, 1, 1) | | (1, 2, 8) | | (1, 1, 1) | | (1, 1, 1) | | (1, 1, 8) | | (1, 1, 8) | | (1, 8, 1) | (269, 128) | | | (128, 2) |
| Prediabetes_2_9 | (1, 4, 2) | | (1, 1, 4) | (1, 4, 1) | | (1, 1, 1) | | (1, 1, 1) | | (1, 1, 1) | | (1, 2, 8) | | (1, 1, 1) | | (1, 1, 1) | | (1, 1, 8) | | (1, 1, 8) | | (1, 8, 1) | (271, 128) | | | (128, 2) |
| Prediabetes_3_10 | (1, 4, 2) | | (1, 1, 4) | (1, 4, 1) | | (1, 1, 1) | | (1, 1, 1) | | (1, 1, 1) | | (1, 2, 8) | | (1, 1, 1) | | (1, 1, 1) | | (1, 1, 8) | | (1, 1, 8) | | (1, 8, 1) | (271, 128) | | | (128, 2) |
| Prediabetes_3_12 | (1, 4, 2) | | (1, 1, 4) | (1, 4, 1) | | (1, 1, 1) | | (1, 1, 1) | | (1, 1, 1) | | (1, 2, 8) | | (1, 1, 1) | | (1, 1, 1) | | (1, 1, 8) | | (1, 1, 8) | | (1, 8, 1) | (275, 128) | | | (128, 2) |
| Prediabetes_3_13 | (1, 4, 2) | | (1, 1, 4) | (1, 4, 1) | | (1, 1, 1) | | (1, 1, 1) | | (1, 1, 1) | | (1, 2, 8) | | (1, 1, 1) | | (1, 1, 1) | | (1, 1, 8) | | (1, 1, 8) | | (1, 8, 1) | (277, 128) | | | (128, 2) |
| Prediabetes_3_5 | (1, 4, 2) | | (1, 1, 4) | (1, 4, 1) | | (1, 1, 1) | | (1, 1, 1) | | (1, 1, 1) | | (1, 2, 8) | | (1, 1, 1) | | (1, 1, 1) | | (1, 1, 8) | | (1, 1, 8) | | (1, 8, 1) | (261, 128) | | | (128, 2) |
| Prediabetes_3_6 | (1, 4, 2) | | (1, 1, 4) | (1, 4, 1) | | (1, 1, 1) | | (1, 1, 1) | | (1, 1, 1) | | (1, 2, 8) | | (1, 1, 1) | | (1, 1, 1) | | (1, 1, 8) | | (1, 1, 8) | | (1, 8, 1) | (263, 128) | | | (128, 2) |
| Prediabetes_3_7 | (1, 4, 2) | | (1, 1, 4) | (1, 4, 1) | | (1, 1, 1) | | (1, 1, 1) | | (1, 1, 1) | | (1, 2, 8) | | (1, 1, 1) | | (1, 1, 1) | | (1, 1, 8) | | (1, 1, 8) | | (1, 8, 1) | (265, 128) | | | (128, 2) |
| Prediabetes_3_8 | (1, 4, 2) | | (1, 1, 4) | (1, 4, 1) | | (1, 1, 1) | | (1, 1, 1) | | (1, 1, 1) | | (1, 2, 8) | | (1, 1, 1) | | (1, 1, 1) | | (1, 1, 8) | | (1, 1, 8) | | (1, 8, 1) | (267, 128) | | | (128, 2) |
| Prediabetes_3_9 | (1, 4, 2) | | (1, 1, 4) | (1, 4, 1) | | (1, 1, 1) | | (1, 1, 1) | | (1, 1, 1) | | (1, 2, 8) | | (1, 1, 1) | | (1, 1, 1) | | (1, 1, 8) | | (1, 1, 8) | | (1, 8, 1) | (269, 128) | | | (128, 2) |
| Prediabetes_4_10 | (1, 4, 2) | | (1, 1, 4) | (1, 4, 1) | | (1, 1, 1) | | (1, 1, 1) | | (1, 1, 1) | | (1, 2, 8) | | (1, 1, 1) | | (1, 1, 1) | | (1, 1, 8) | | (1, 1, 8) | | (1, 8, 1) | (269, 128) | | | (128, 2) |
| Prediabetes_4_11 | (1, 4, 2) | | (1, 1, 4) | (1, 4, 1) | | (1, 1, 1) | | (1, 1, 1) | | (1, 1, 1) | | (1, 2, 8) | | (1, 1, 1) | | (1, 1, 1) | | (1, 1, 8) | | (1, 1, 8) | | (1, 8, 1) | (271, 128) | | | (128, 2) |
| Prediabetes_4_12 | (1, 4, 2) | | (1, 1, 4) | (1, 4, 1) | | (1, 1, 1) | | (1, 1, 1) | | (1, 1, 1) | | (1, 2, 8) | | (1, 1, 1) | | (1, 1, 1) | | (1, 1, 8) | | (1, 1, 8) | | (1, 8, 1) | (273, 128) | | | (128, 2) |
| Prediabetes_4_13 | (1, 4, 2) | | (1, 1, 4) | (1, 4, 1) | | (1, 1, 1) | | (1, 1, 1) | | (1, 1, 1) | | (1, 2, 8) | | (1, 1, 1) | | (1, 1, 1) | | (1, 1, 8) | | (1, 1, 8) | | (1, 8, 1) | (275, 128) | | | (128, 2) |
| Prediabetes_4_7 | (1, 4, 2) | | (1, 1, 4) | (1, 4, 1) | | (1, 1, 1) | | (1, 1, 1) | | (1, 1, 1) | | (1, 2, 8) | | (1, 1, 1) | | (1, 1, 1) | | (1, 1, 8) | | (1, 1, 8) | | (1, 8, 1) | (263, 128) | | | (128, 2) |
| Prediabetes_4_8 | (1, 4, 2) | | (1, 1, 4) | (1, 4, 1) | | (1, 1, 1) | | (1, 1, 1) | | (1, 1, 1) | | (1, 2, 8) | | (1, 1, 1) | | (1, 1, 1) | | (1, 1, 8) | | (1, 1, 8) | | (1, 8, 1) | (265, 128) | | | (128, 2) |
| Prediabetes_4_9 | (1, 4, 2) | | (1, 1, 4) | (1, 4, 1) | | (1, 1, 1) | | (1, 1, 1) | | (1, 1, 1) | | (1, 2, 8) | | (1, 1, 1) | | (1, 1, 1) | | (1, 1, 8) | | (1, 1, 8) | | (1, 8, 1) | (267, 128) | | | (128, 2) |
| Prediabetes_5_10 | (1, 4, 2) | | (1, 1, 4) | (1, 4, 1) | | (1, 1, 1) | | (1, 1, 1) | | (1, 1, 1) | | (1, 2, 8) | | (1, 1, 1) | | (1, 1, 1) | | (1, 1, 8) | | (1, 1, 8) | | (1, 8, 1) | (267, 128) | | | (128, 2) |
| Prediabetes_5_11 | (1, 4, 2) | | (1, 1, 4) | (1, 4, 1) | | (1, 1, 1) | | (1, 1, 1) | | (1, 1, 1) | | (1, 2, 8) | | (1, 1, 1) | | (1, 1, 1) | | (1, 1, 8) | | (1, 1, 8) | | (1, 8, 1) | (269, 128) | | | (128, 2) |
| Prediabetes_5_12 | (1, 4, 2) | | (1, 1, 4) | (1, 4, 1) | | (1, 1, 1) | | (1, 1, 1) | | (1, 1, 1) | | (1, 2, 8) | | (1, 1, 1) | | (1, 1, 1) | | (1, 1, 8) | | (1, 1, 8) | | (1, 8, 1) | (271, 128) | | | (128, 2) |
| Prediabetes_5_13 | (1, 4, 2) | | (1, 1, 4) | (1, 4, 1) | | (1, 1, 1) | | (1, 1, 1) | | (1, 1, 1) | | (1, 2, 8) | | (1, 1, 1) | | (1, 1, 1) | | (1, 1, 8) | | (1, 1, 8) | | (1, 8, 1) | (273, 128) | | | (128, 2) |
| Prediabetes_5_7 | (1, 4, 2) | | (1, 1, 4) | (1, 4, 1) | | (1, 1, 1) | | (1, 1, 1) | | (1, 1, 1) | | (1, 2, 8) | | (1, 1, 1) | | (1, 1, 1) | | (1, 1, 8) | | (1, 1, 8) | | (1, 8, 1) | (261, 128) | | | (128, 2) |
| Prediabetes_5_9 | (1, 4, 2) | | (1, 1, 4) | (1, 4, 1) | | (1, 1, 1) | | (1, 1, 1) | | (1, 1, 1) | | (1, 2, 8) | | (1, 1, 1) | | (1, 1, 1) | | (1, 1, 8) | | (1, 1, 8) | | (1, 8, 1) | (265, 128) | | | (128, 2) |
| Prediabetes_6_10 | (1, 4, 2) | | (1, 1, 4) | (1, 4, 1) | | (1, 1, 1) | | (1, 1, 1) | | (1, 1, 1) | | (1, 2, 8) | | (1, 1, 1) | | (1, 1, 1) | | (1, 1, 8) | | (1, 1, 8) | | (1, 8, 1) | (265, 128) | | | (128, 2) |
| Prediabetes_6_11 | (1, 4, 2) | | (1, 1, 4) | (1, 4, 1) | | (1, 1, 1) | | (1, 1, 1) | | (1, 1, 1) | | (1, 2, 8) | | (1, 1, 1) | | (1, 1, 1) | | (1, 1, 8) | | (1, 1, 8) | | (1, 8, 1) | (267, 128) | | | (128, 2) |
| Prediabetes_6_12 | (1, 4, 2) | | (1, 1, 4) | (1, 4, 1) | | (1, 1, 1) | | (1, 1, 1) | | (1, 1, 1) | | (1, 2, 8) | | (1, 1, 1) | | (1, 1, 1) | | (1, 1, 8) | | (1, 1, 8) | | (1, 8, 1) | (269, 128) | | | (128, 2) |
| Prediabetes_6_13 | (1, 4, 2) | | (1, 1, 4) | (1, 4, 1) | | (1, 1, 1) | | (1, 1, 1) | | (1, 1, 1) | | (1, 2, 8) | | (1, 1, 1) | | (1, 1, 1) | | (1, 1, 8) | | (1, 1, 8) | | (1, 8, 1) | (271, 128) | | | (128, 2) |
| Prediabetes_6_8 | (1, 4, 2) | | (1, 1, 4) | (1, 4, 1) | | (1, 1, 1) | | (1, 1, 1) | | (1, 1, 1) | | (1, 2, 8) | | (1, 1, 1) | | (1, 1, 1) | | (1, 1, 8) | | (1, 1, 8) | | (1, 8, 1) | (261, 128) | | | (128, 2) |
| Prediabetes_7_10 | (1, 4, 2) | | (1, 1, 4) | (1, 4, 1) | | (1, 1, 1) | | (1, 1, 1) | | (1, 1, 1) | | (1, 2, 8) | | (1, 1, 1) | | (1, 1, 1) | | (1, 1, 8) | | (1, 1, 8) | | (1, 8, 1) | (263, 128) | | | (128, 2) |
| Prediabetes_7_11 | (1, 4, 2) | | (1, 1, 4) | (1, 4, 1) | | (1, 1, 1) | | (1, 1, 1) | | (1, 1, 1) | | (1, 2, 8) | | (1, 1, 1) | | (1, 1, 1) | | (1, 1, 8) | | (1, 1, 8) | | (1, 8, 1) | (265, 128) | | | (128, 2) |
| Prediabetes_7_12 | (1, 4, 2) | | (1, 1, 4) | (1, 4, 1) | | (1, 1, 1) | | (1, 1, 1) | | (1, 1, 1) | | (1, 2, 8) | | (1, 1, 1) | | (1, 1, 1) | | (1, 1, 8) | | (1, 1, 8) | | (1, 8, 1) | (267, 128) | | | (128, 2) |
| Prediabetes_7_13 | (1, 4, 2) | | (1, 1, 4) | (1, 4, 1) | | (1, 1, 1) | | (1, 1, 1) | | (1, 1, 1) | | (1, 2, 8) | | (1, 1, 1) | | (1, 1, 1) | | (1, 1, 8) | | (1, 1, 8) | | (1, 8, 1) | (269, 128) | | | (128, 2) |
| Prediabetes_7_9 | (1, 4, 2) | | (1, 1, 4) | (1, 4, 1) | | (1, 1, 1) | | (1, 1, 1) | | (1, 1, 1) | | (1, 2, 8) | | (1, 1, 1) | | (1, 1, 1) | | (1, 1, 8) | | (1, 1, 8) | | (1, 8, 1) | (261, 128) | | | (128, 2) |
| Prediabetes_8_10 | (1, 4, 2) | | (1, 1, 4) | (1, 4, 1) | | (1, 1, 1) | | (1, 1, 1) | | (1, 1, 1) | | (1, 2, 8) | | (1, 1, 1) | | (1, 1, 1) | | (1, 1, 8) | | (1, 1, 8) | | (1, 8, 1) | (261, 128) | | | (128, 2) |
| Prediabetes_8_11 | (1, 4, 2) | | (1, 1, 4) | (1, 4, 1) | | (1, 1, 1) | | (1, 1, 1) | | (1, 1, 1) | | (1, 2, 8) | | (1, 1, 1) | | (1, 1, 1) | | (1, 1, 8) | | (1, 1, 8) | | (1, 8, 1) | (263, 128) | | | (128, 2) |
| Prediabetes_8_12 | (1, 4, 2) | | (1, 1, 4) | (1, 4, 1) | | (1, 1, 1) | | (1, 1, 1) | | (1, 1, 1) | | (1, 2, 8) | | (1, 1, 1) | | (1, 1, 1) | | (1, 1, 8) | | (1, 1, 8) | | (1, 8, 1) | (265, 128) | | | (128, 2) |
| Prediabetes_8_13 | (1, 4, 2) | | (1, 1, 4) | (1, 4, 1) | | (1, 1, 1) | | (1, 1, 1) | | (1, 1, 1) | | (1, 2, 8) | | (1, 1, 1) | | (1, 1, 1) | | (1, 1, 8) | | (1, 1, 8) | | (1, 8, 1) | (267, 128) | | | (128, 2) |
| Prediabetes_9_11 | (1, 4, 2) | | (1, 1, 4) | (1, 4, 1) | | (1, 1, 1) | | (1, 1, 1) | | (1, 1, 1) | | (1, 2, 8) | | (1, 1, 1) | | (1, 1, 1) | | (1, 1, 8) | | (1, 1, 8) | | (1, 8, 1) | (261, 128) | | | (128, 2) |
| Prediabetes_9_12 | (1, 4, 2) | | (1, 1, 4) | (1, 4, 1) | | (1, 1, 1) | | (1, 1, 1) | | (1, 1, 1) | | (1, 2, 8) | | (1, 1, 1) | | (1, 1, 1) | | (1, 1, 8) | | (1, 1, 8) | | (1, 8, 1) | (263, 128) | | | (128, 2) |
| Prediabetes_9_13 | (1, 4, 2) | | (1, 1, 4) | (1, 4, 1) | | (1, 1, 1) | | (1, 1, 1) | | (1, 1, 1) | | (1, 2, 8) | | (1, 1, 1) | | (1, 1, 1) | | (1, 1, 8) | | (1, 1, 8) | | (1, 8, 1) | (265, 128) | | | (128, 2) |

Each encoder block contain an MHA and a feed-forward block. MHA stands for Multi-Head Attention layer. Each feed-forward unit consists of two Conv 1D that its embedding size includes kernel width, input size, and number of convolution kernels. Embedding size of each Dense layer includes input size an output size.

**Supplementary Table 15.** Embedding size of CRNNs

| Cohort Name | Conv 1D | | Conv 1D | | LSTM | | Dense |  |
| --- | --- | --- | --- | --- | --- | --- | --- | --- |
| T2D_10_12 | (8, 1, 64) | | (8, 64, 128) | (128, 16) | | | (4, 2) | |
| T2D_10_13 | (8, 1, 64) | | (8, 64, 128) | (128, 16) | | | (4, 2) | |
| T2D_10_14 | (8, 1, 64) | | (8, 64, 128) | (128, 16) | | | (4, 2) | |
| T2D_11_13 | (8, 1, 64) | | (8, 64, 128) | (128, 16) | | | (4, 2) | |
| T2D_12_14 | (8, 1, 64) | | (8, 64, 128) | (128, 16) | | | (4, 2) | |
| T2D_3_10 | (8, 1, 64) | | (8, 64, 128) | (128, 16) | | | (4, 2) | |
| T2D_3_11 | (8, 1, 64) | | (8, 64, 128) | (128, 16) | | | (4, 2) | |
| T2D_3_12 | (8, 1, 64) | (8, 64, 128) | | (128, 16) | | (4, 2) | | |
| T2D_3_13 | (8, 1, 64) | (8, 64, 128) | | (128, 16) | | (4, 2) | | |
| T2D_3_14 | (8, 1, 64) | (8, 64, 128) | | (128, 16) | | (4, 2) | | |
| T2D_3_5 | (8, 1, 64) | (8, 64, 128) | | (128, 16) | | (4, 2) | | |
| T2D_3_6 | (8, 1, 64) | (8, 64, 128) | | (128, 16) | | (4, 2) | | |
| T2D_3_7 | (8, 1, 64) | (8, 64, 128) | | (128, 16) | | (4, 2) | | |
| T2D_3_8 | (8, 1, 64) | (8, 64, 128) | | (128, 16) | | (4, 2) | | |
| T2D_3_9 | (8, 1, 64) | (8, 64, 128) | | (128, 16) | | (4, 2) | | |
| T2D_4_10 | (8, 1, 64) | (8, 64, 128) | | (128, 16) | | (4, 2) | | |
| T2D_4_11 | (8, 1, 64) | (8, 64, 128) | | (128, 16) | | (4, 2) | | |
| T2D_4_12 | (8, 1, 64) | (8, 64, 128) | | (128, 16) | | (4, 2) | | |
| T2D_4_14 | (8, 1, 64) | (8, 64, 128) | | (128, 16) | | (4, 2) | | |
| T2D_4_6 | (8, 1, 64) | (8, 64, 128) | | (128, 16) | | (4, 2) | | |
| T2D_4_7 | (8, 1, 64) | (8, 64, 128) | | (128, 16) | | (4, 2) | | |
| T2D_4_8 | (8, 1, 64) | (8, 64, 128) | | (128, 16) | | (4, 2) | | |
| T2D_4_9 | (8, 1, 64) | (8, 64, 128) | | (128, 16) | | (4, 2) | | |
| T2D_5_10 | (8, 1, 64) | (8, 64, 128) | | (128, 16) | | (4, 2) | | |
| T2D_5_11 | (8, 1, 64) | (8, 64, 128) | | (128, 16) | | (4, 2) | | |
| T2D_5_12 | (8, 1, 64) | (8, 64, 128) | | (128, 16) | | (4, 2) | | |
| T2D_5_13 | (8, 1, 64) | (8, 64, 128) | | (128, 16) | | (4, 2) | | |
| T2D_5_14 | (8, 1, 64) | (8, 64, 128) | | (128, 16) | | (4, 2) | | |
| T2D_5_7 | (8, 1, 64) | (8, 64, 128) | | (128, 16) | | (4, 2) | | |
| T2D_5_8 | (8, 1, 64) | (8, 64, 128) | | (128, 16) | | (4, 2) | | |
| T2D_5_9 | (8, 1, 64) | (8, 64, 128) | | (128, 16) | | (4, 2) | | |
| T2D_6_10 | (8, 1, 64) | (8, 64, 128) | | (128, 16) | | (4, 2) | | |
| T2D_6_11 | (8, 1, 64) | (8, 64, 128) | | (128, 16) | | (4, 2) | | |
| T2D_6_12 | (8, 1, 64) | (8, 64, 128) | | (128, 16) | | (4, 2) | | |
| T2D_6_13 | (8, 1, 64) | (8, 64, 128) | | (128, 16) | | (4, 2) | | |
| T2D_6_14 | (8, 1, 64) | (8, 64, 128) | | (128, 16) | | (4, 2) | | |
| T2D_6_8 | (8, 1, 64) | (8, 64, 128) | | (128, 16) | | (4, 2) | | |
| T2D_6_9 | (8, 1, 64) | (8, 64, 128) | | (128, 16) | | (4, 2) | | |
| T2D_7_10 | (8, 1, 64) | (8, 64, 128) | | (128, 16) | | (4, 2) | | |
| T2D_7_11 | (8, 1, 64) | (8, 64, 128) | | (128, 16) | | (4, 2) | | |
| T2D_7_12 | (8, 1, 64) | (8, 64, 128) | | (128, 16) | | (4, 2) | | |
| T2D_7_13 | (8, 1, 64) | (8, 64, 128) | | (128, 16) | | (4, 2) | | |
| T2D_7_14 | (8, 1, 64) | (8, 64, 128) | | (128, 16) | | (4, 2) | | |
| T2D_7_9 | (8, 1, 64) | (8, 64, 128) | | (128, 16) | | (4, 2) | | |
| T2D_8_11 | (8, 1, 64) | (8, 64, 128) | | (128, 16) | | (4, 2) | | |
| T2D_8_12 | (8, 1, 64) | (8, 64, 128) | | (128, 16) | | (4, 2) | | |
| T2D_8_13 | (8, 1, 64) | (8, 64, 128) | | (128, 16) | | (4, 2) | | |
| T2D_8_14 | (8, 1, 64) | (8, 64, 128) | | (128, 16) | | (4, 2) | | |
| T2D_9_11 | (8, 1, 64) | (8, 64, 128) | | (128, 16) | | (4, 2) | | |
| T2D_9_12 | (8, 1, 64) | (8, 64, 128) | | (128, 16) | | (4, 2) | | |
| T2D_9_13 | (8, 1, 64) | (8, 64, 128) | | (128, 16) | | (4, 2) | | |
| T2D_9_14 | (8, 1, 64) | (8, 64, 128) | | (128, 16) | | (4, 2) | | |
| Metabolic_10_12 | (8, 1, 64) | (8, 64, 128) | | (128, 16) | | (4, 2) | | |
| Metabolic_10_13 | (8, 1, 64) | (8, 64, 128) | | (128, 16) | | (4, 2) | | |
| Metabolic_2_10 | (8, 1, 64) | (8, 64, 128) | | (128, 16) | | (4, 2) | | |
| Metabolic_2_11 | (8, 1, 64) | (8, 64, 128) | | (128, 16) | | (4, 2) | | |
| Metabolic_2_12 | (8, 1, 64) | (8, 64, 128) | | (128, 16) | | (4, 2) | | |
| Metabolic_2_13 | (8, 1, 64) | (8, 64, 128) | | (128, 16) | | (4, 2) | | |
| Metabolic_2_4 | (8, 1, 64) | (8, 64, 128) | | (128, 16) | | (4, 2) | | |
| Metabolic_2_5 | (8, 1, 64) | (8, 64, 128) | | (128, 16) | | (4, 2) | | |
| Metabolic_2_6 | (8, 1, 64) | (8, 64, 128) | | (128, 16) | | (4, 2) | | |
| Metabolic_2_7 | (8, 1, 64) | (8, 64, 128) | | (128, 16) | | (4, 2) | | |
| Metabolic_2_8 | (8, 1, 64) | (8, 64, 128) | | (128, 16) | | (4, 2) | | |
| Metabolic_2_9 | (8, 1, 64) | (8, 64, 128) | | (128, 16) | | (4, 2) | | |
| Metabolic_3_10 | (8, 1, 64) | (8, 64, 128) | | (128, 16) | | (4, 2) | | |
| Metabolic_3_11 | (8, 1, 64) | (8, 64, 128) | | (128, 16) | | (4, 2) | | |
| Metabolic_3_12 | (8, 1, 64) | (8, 64, 128) | | (128, 16) | | (4, 2) | | |
| Metabolic_3_13 | (8, 1, 64) | (8, 64, 128) | | (128, 16) | | (4, 2) | | |
| Metabolic_3_5 | (8, 1, 64) | (8, 64, 128) | | (128, 16) | | (4, 2) | | |
| Metabolic_3_6 | (8, 1, 64) | (8, 64, 128) | | (128, 16) | | (4, 2) | | |
| Metabolic_3_7 | (8, 1, 64) | (8, 64, 128) | | (128, 16) | | (4, 2) | | |
| Metabolic_3_8 | (8, 1, 64) | (8, 64, 128) | | (128, 16) | | (4, 2) | | |
| Metabolic_4_10 | (8, 1, 64) | (8, 64, 128) | | (128, 16) | | (4, 2) | | |
| Metabolic_4_13 | (8, 1, 64) | (8, 64, 128) | | (128, 16) | | (4, 2) | | |
| Metabolic_4_6 | (8, 1, 64) | (8, 64, 128) | | (128, 16) | | (4, 2) | | |
| Metabolic_4_7 | (8, 1, 64) | (8, 64, 128) | | (128, 16) | | (4, 2) | | |
| Metabolic_4_8 | (8, 1, 64) | (8, 64, 128) | | (128, 16) | | (4, 2) | | |
| Metabolic_4_9 | (8, 1, 64) | (8, 64, 128) | | (128, 16) | | (4, 2) | | |
| Metabolic_5_10 | (8, 1, 64) | (8, 64, 128) | | (128, 16) | | (4, 2) | | |
| Metabolic_5_11 | (8, 1, 64) | (8, 64, 128) | | (128, 16) | | (4, 2) | | |
| Metabolic_5_12 | (8, 1, 64) | (8, 64, 128) | | (128, 16) | | (4, 2) | | |
| Metabolic_5_7 | (8, 1, 64) | (8, 64, 128) | | (128, 16) | | (4, 2) | | |
| Metabolic_5_8 | (8, 1, 64) | (8, 64, 128) | | (128, 16) | | (4, 2) | | |
| Metabolic_5_9 | (8, 1, 64) | (8, 64, 128) | | (128, 16) | | (4, 2) | | |
| Metabolic_6_10 | (8, 1, 64) | (8, 64, 128) | | (128, 16) | | (4, 2) | | |
| Metabolic_6_11 | (8, 1, 64) | (8, 64, 128) | | (128, 16) | | (4, 2) | | |
| Metabolic_6_12 | (8, 1, 64) | (8, 64, 128) | | (128, 16) | | (4, 2) | | |
| Metabolic_6_13 | (8, 1, 64) | (8, 64, 128) | | (128, 16) | | (4, 2) | | |
| Metabolic_6_8 | (8, 1, 64) | (8, 64, 128) | | (128, 16) | | (4, 2) | | |
| Metabolic_6_9 | (8, 1, 64) | (8, 64, 128) | | (128, 16) | | (4, 2) | | |
| Metabolic_7_11 | (8, 1, 64) | (8, 64, 128) | | (128, 16) | | (4, 2) | | |
| Metabolic_7_12 | (8, 1, 64) | (8, 64, 128) | | (128, 16) | | (4, 2) | | |
| Metabolic_7_13 | (8, 1, 64) | (8, 64, 128) | | (128, 16) | | (4, 2) | | |
| Metabolic_7_9 | (8, 1, 64) | (8, 64, 128) | | (128, 16) | | (4, 2) | | |
| Metabolic_8_10 | (8, 1, 64) | (8, 64, 128) | | (128, 16) | | (4, 2) | | |
| Metabolic_8_11 | (8, 1, 64) | (8, 64, 128) | | (128, 16) | | (4, 2) | | |
| Metabolic_8_12 | (8, 1, 64) | (8, 64, 128) | | (128, 16) | | (4, 2) | | |
| Metabolic_8_13 | (8, 1, 64) | (8, 64, 128) | | (128, 16) | | (4, 2) | | |
| Metabolic_9_11 | (8, 1, 64) | (8, 64, 128) | | (128, 16) | | (4, 2) | | |
| Metabolic_9_12 | (8, 1, 64) | (8, 64, 128) | | (128, 16) | | (4, 2) | | |
| Metabolic_9_13 | (8, 1, 64) | (8, 64, 128) | | (128, 16) | | (4, 2) | | |
| Prediabetes_10_12 | (8, 1, 64) | (8, 64, 128) | | (128, 16) | | (4, 2) | | |
| Prediabetes_10_13 | (8, 1, 64) | (8, 64, 128) | | (128, 16) | | (4, 2) | | |
| Prediabetes_11_13 | (8, 1, 64) | (8, 64, 128) | | (128, 16) | | (4, 2) | | |
| Prediabetes_2_10 | (8, 1, 64) | (8, 64, 128) | | (128, 16) | | (4, 2) | | |
| Prediabetes_2_11 | (8, 1, 64) | (8, 64, 128) | | (128, 16) | | (4, 2) | | |
| Prediabetes_2_12 | (8, 1, 64) | (8, 64, 128) | | (128, 16) | | (4, 2) | | |
| Prediabetes_2_13 | (8, 1, 64) | (8, 64, 128) | | (128, 16) | | (4, 2) | | |
| Prediabetes_2_4 | (8, 1, 64) | (8, 64, 128) | | (128, 16) | | (4, 2) | | |
| Prediabetes_2_5 | (8, 1, 64) | (8, 64, 128) | | (128, 16) | | (4, 2) | | |
| Prediabetes_2_6 | (8, 1, 64) | (8, 64, 128) | | (128, 16) | | (4, 2) | | |
| Prediabetes_2_8 | (8, 1, 64) | (8, 64, 128) | | (128, 16) | | (4, 2) | | |
| Prediabetes_2_9 | (8, 1, 64) | (8, 64, 128) | | (128, 16) | | (4, 2) | | |
| Prediabetes_3_10 | (8, 1, 64) | (8, 64, 128) | | (128, 16) | | (4, 2) | | |
| Prediabetes_3_11 | (8, 1, 64) | (8, 64, 128) | | (128, 16) | | (4, 2) | | |
| Prediabetes_3_12 | (8, 1, 64) | (8, 64, 128) | | (128, 16) | | (4, 2) | | |
| Prediabetes_3_13 | (8, 1, 64) | (8, 64, 128) | | (128, 16) | | (4, 2) | | |
| Prediabetes_3_5 | (8, 1, 64) | (8, 64, 128) | | (128, 16) | | (4, 2) | | |
| Prediabetes_3_6 | (8, 1, 64) | (8, 64, 128) | | (128, 16) | | (4, 2) | | |
| Prediabetes_3_7 | (8, 1, 64) | (8, 64, 128) | | (128, 16) | | (4, 2) | | |
| Prediabetes_3_8 | (8, 1, 64) | (8, 64, 128) | | (128, 16) | | (4, 2) | | |
| Prediabetes_3_9 | (8, 1, 64) | (8, 64, 128) | | (128, 16) | | (4, 2) | | |
| Prediabetes_4_10 | (8, 1, 64) | (8, 64, 128) | | (128, 16) | | (4, 2) | | |
| Prediabetes_4_11 | (8, 1, 64) | (8, 64, 128) | | (128, 16) | | (4, 2) | | |
| Prediabetes_4_12 | (8, 1, 64) | (8, 64, 128) | | (128, 16) | | (4, 2) | | |
| Prediabetes_4_13 | (8, 1, 64) | (8, 64, 128) | | (128, 16) | | (4, 2) | | |
| Prediabetes_4_6 | (8, 1, 64) | (8, 64, 128) | | (128, 16) | | (4, 2) | | |
| Prediabetes_4_7 | (8, 1, 64) | (8, 64, 128) | | (128, 16) | | (4, 2) | | |
| Prediabetes_4_8 | (8, 1, 64) | (8, 64, 128) | | (128, 16) | | (4, 2) | | |
| Prediabetes_4_9 | (8, 1, 64) | (8, 64, 128) | | (128, 16) | | (4, 2) | | |
| Prediabetes_5_10 | (8, 1, 64) | (8, 64, 128) | | (128, 16) | | (4, 2) | | |
| Prediabetes_5_11 | (8, 1, 64) | (8, 64, 128) | | (128, 16) | | (4, 2) | | |
| Prediabetes_5_12 | (8, 1, 64) | (8, 64, 128) | | (128, 16) | | (4, 2) | | |
| Prediabetes_5_13 | (8, 1, 64) | (8, 64, 128) | | (128, 16) | | (4, 2) | | |
| Prediabetes_5_7 | (8, 1, 64) | (8, 64, 128) | | (128, 16) | | (4, 2) | | |
| Prediabetes_5_8 | (8, 1, 64) | (8, 64, 128) | | (128, 16) | | (4, 2) | | |
| Prediabetes_6_10 | (8, 1, 64) | (8, 64, 128) | | (128, 16) | | (4, 2) | | |
| Prediabetes_6_11 | (8, 1, 64) | (8, 64, 128) | | (128, 16) | | (4, 2) | | |
| Prediabetes_6_12 | (8, 1, 64) | (8, 64, 128) | | (128, 16) | | (4, 2) | | |
| Prediabetes_6_8 | (8, 1, 64) | (8, 64, 128) | | (128, 16) | | (4, 2) | | |
| Prediabetes_6_9 | (8, 1, 64) | (8, 64, 128) | | (128, 16) | | (4, 2) | | |
| Prediabetes_7_10 | (8, 1, 64) | (8, 64, 128) | | (128, 16) | | (4, 2) | | |
| Prediabetes_7_11 | (8, 1, 64) | (8, 64, 128) | | (128, 16) | | (4, 2) | | |
| Prediabetes_7_12 | (8, 1, 64) | (8, 64, 128) | | (128, 16) | | (4, 2) | | |
| Prediabetes_7_13 | (8, 1, 64) | (8, 64, 128) | | (128, 16) | | (4, 2) | | |
| Prediabetes_7_9 | (8, 1, 64) | (8, 64, 128) | | (128, 16) | | (4, 2) | | |
| Prediabetes_8_10 | (8, 1, 64) | (8, 64, 128) | | (128, 16) | | (4, 2) | | |
| Prediabetes_8_11 | (8, 1, 64) | (8, 64, 128) | | (128, 16) | | (4, 2) | | |
| Prediabetes_8_12 | (8, 1, 64) | (8, 64, 128) | | (128, 16) | | (4, 2) | | |
| Prediabetes_8_13 | (8, 1, 64) | (8, 64, 128) | | (128, 16) | | (4, 2) | | |
| Prediabetes_9_11 | (8, 1, 64) | (8, 64, 128) | | (128, 16) | | (4, 2) | | |
| Prediabetes_9_12 | (8, 1, 64) | (8, 64, 128) | | (128, 16) | | (4, 2) | | |

Conv 1D has three features of kernel width, input size, and number of kernels. LSTM stands for Long Short-Term Memory which has two features of input size and number of units can be calculated from the second feature divide by 4. Dense layer has two features of input size an output size.

# Supplementary References:

1 Cheng HT, Koc L, Harmsen J, *et al.* Wide & deep learning for recommender systems. *ACM Int Conf Proceeding Ser* 2016;**15**-**September**-**2016**:7–10. doi:10.1145/2988450.2988454

2 Javidi H, Mariam A, Khademi G, *et al.* Identification of robust deep neural network models of longitudinal clinical measurements. *npj Digit Med* 2022;**5**:1–11. doi:10.1038/s41746-022-00651-4

3 Kuczmarski RJ. 2000 CDC growth charts for the United States : methods and development. 2002;**11**:1–203.https://stacks.cdc.gov/view/cdc/6451

4 Skinner AC, Ravanbakht SN, Skelton JA, *et al.* Prevalence of Obesity and Severe Obesity in US Children, 1999–2016. *Pediatrics* 2018;**141**:1999–2016. doi:10.1542/PEDS.2017-3459

5 Zimmet P, Alberti GKMM, Kaufman F, *et al.* The metabolic syndrome in children and adolescents - an IDF consensus report. *Pediatr Diabetes* 2007;**8**:299–306. doi:10.1111/J.1399-5448.2007.00271.X

6 Li L, Jamieson K, DeSalvo G, *et al.* Hyperband: A Novel Bandit-Based Approach to Hyperparameter Optimization. *J Mach Learn Res* 2016;**18**:1–52. doi:10.48550/arxiv.1603.06560

7 Bergstra J, Ca JB, Ca YB. Random Search for Hyper-Parameter Optimization Yoshua Bengio. *J Mach Learn Res* 2012;**13**:281–305.http://scikit-learn.sourceforge.net. (accessed 15 Mar 2022).

8 R Core Team (2013). R: A language and environment for statistical computing. 2021.https://www.r-project.org/

9 Robin X, Turck N, Hainard A, *et al.* pROC: An open-source package for R and S+ to analyze and compare ROC curves. *BMC Bioinformatics* 2011;**12**:1–8. doi:10.1186/1471-2105-12-77/TABLES/3

10 Kuhn M. Building Predictive Models in R Using the caret Package. *J Stat Softw* 2008;**28**:1–26. doi:10.18637/JSS.V028.I05

11 Van Rossum G, Drake Jr FL. *Python 3 Reference Manual*. Scotts Valley, CA: : CreateSpace 2009.

12 Lundberg SM, Allen PG, Lee S-I. A Unified Approach to Interpreting Model Predictions. In: *Conference on Neural Information Processing Systems (NIPS)*. 2017. https://github.com/slundberg/shap (accessed 18 Feb 2022).

13 Pedregosa FABIANPEDREGOSA F, Michel V, Grisel OLIVIERGRISEL O, *et al.* Scikit-learn: Machine Learning in Python. *J Mach Learn Res* 2011;**12**:2825–30.http://jmlr.org/papers/v12/pedregosa11a.html (accessed 7 Mar 2023).

14 O’Malley, Tom and Bursztein, Elie and Long, James and Chollet, Fran\c{c}ois and Jin, Haifeng and Invernizzi L and others. KerasTuner. 2019.https://github.com/keras-team/keras-tuner
